# Supplementary figures and images for: Analysis of national and subnational prevalence of adolescent pregnancy and changes in the associated sexual behaviours and sociodemographic determinants across three decades in Ghana, 1988–2019
Source: BMJ Open. 2023 Mar 17;13(3):e068117. doi: 10.1136/bmjopen-2022-068117 (PMC10030779; doi:10.1136/bmjopen-2022-068117)

**Supplementary Figure 1. Conceptual model of factors associated with adolescent pregnancy**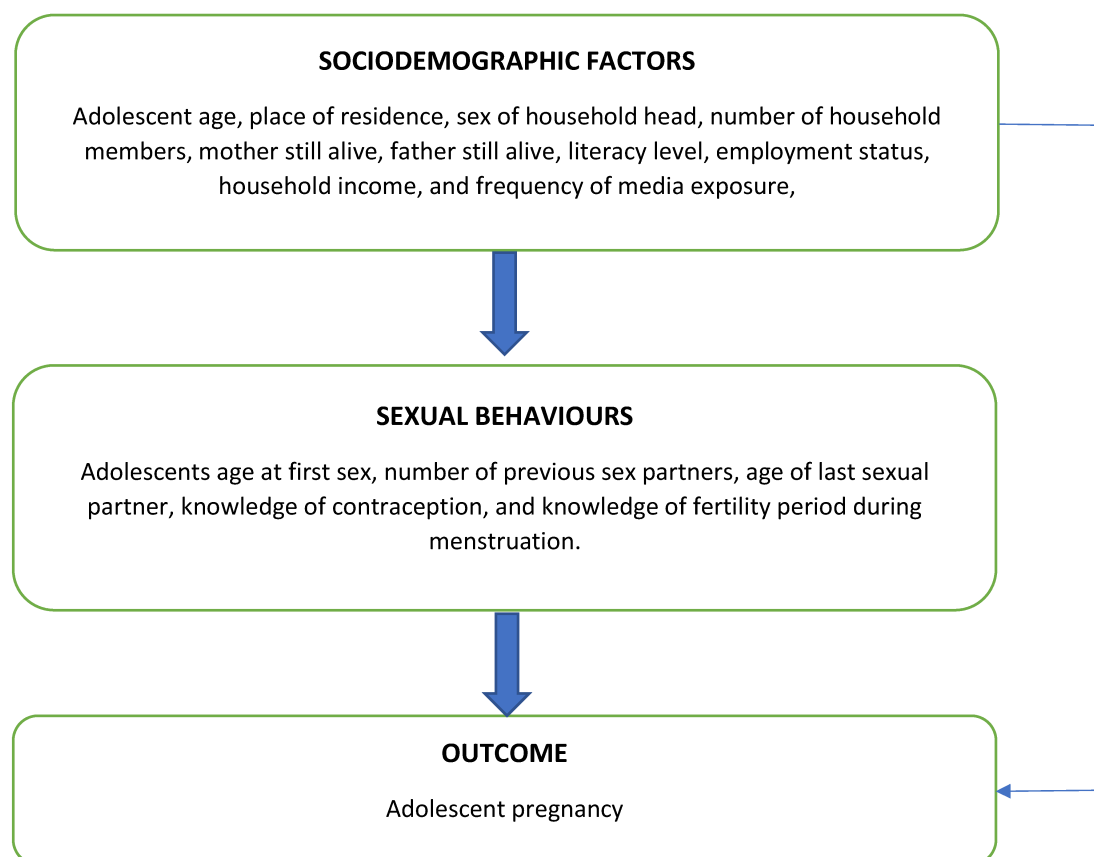

Supplement: Supplementary data [file bmjopen-2022-068117supp001.pdf]

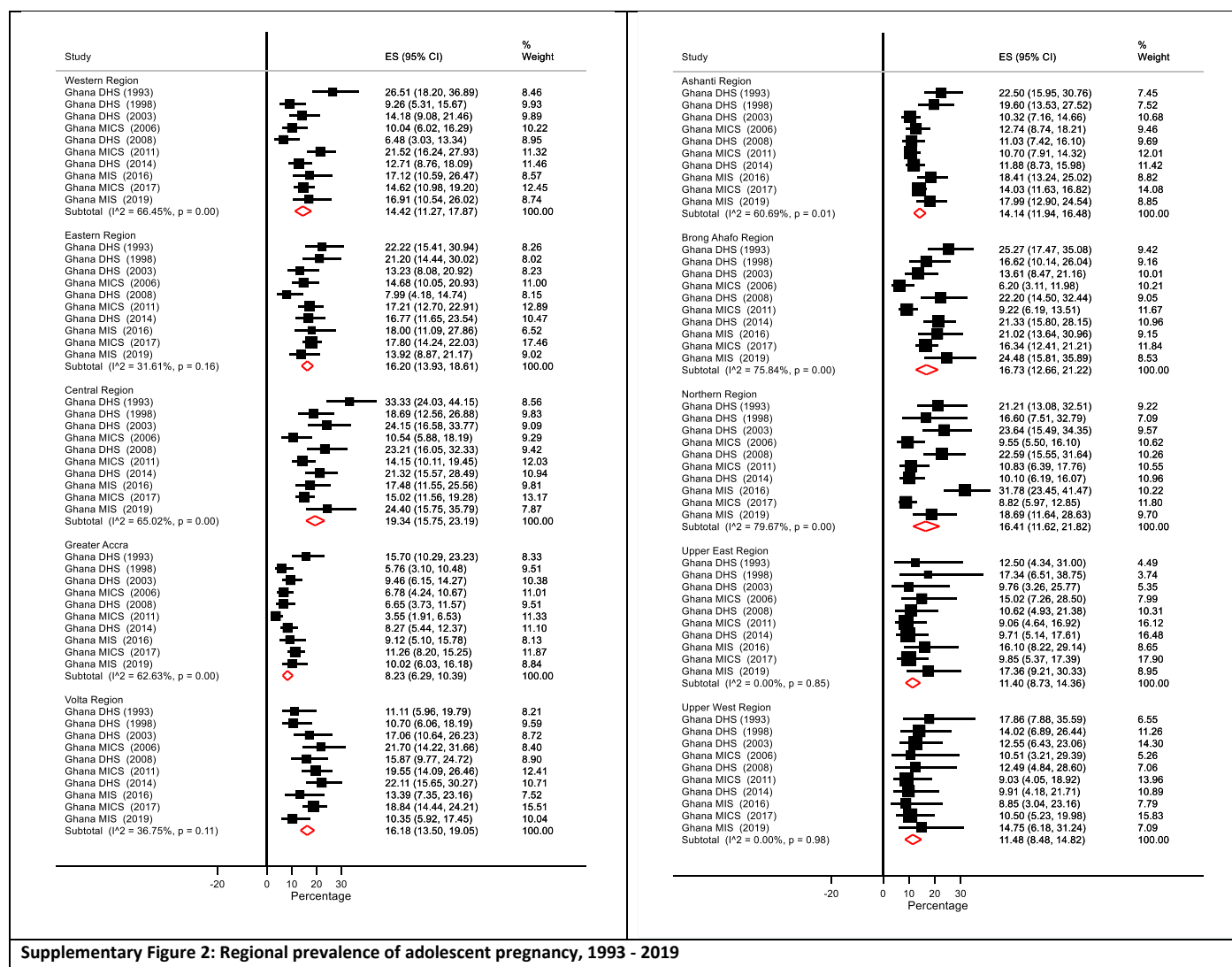

Supplementary Figure 2: Regional prevalence of adolescent pregnancy, 1993 - 2019

Supplement: Supplementary data [file bmjopen-2022-068117supp002.pdf]

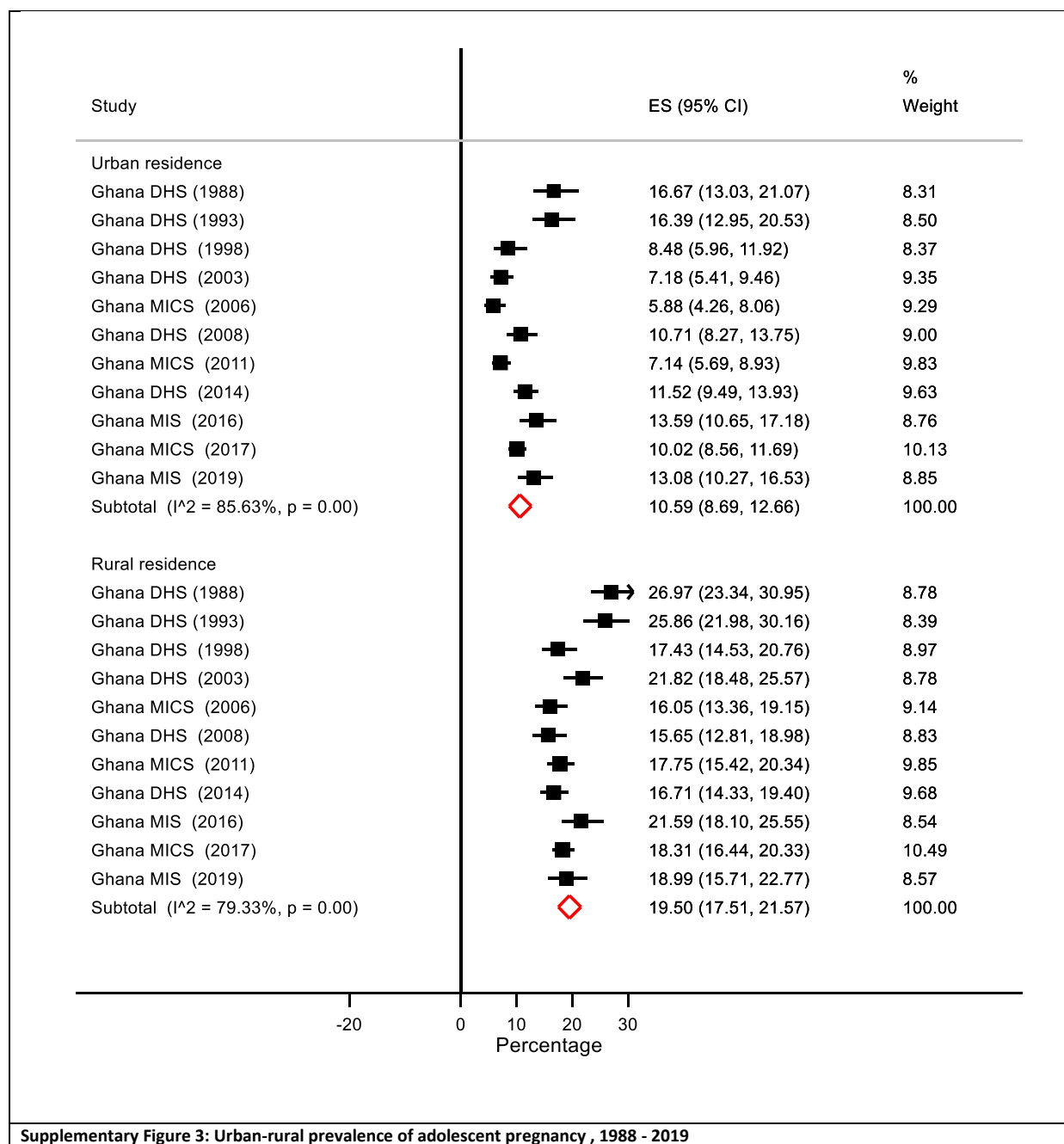

Supplement: Supplementary data [file bmjopen-2022-068117supp003.pdf]
