# Supplementary material for: Analysis of national and subnational prevalence of adolescent pregnancy and changes in the associated sexual behaviours and sociodemographic determinants across three decades in Ghana, 1988–2019
Source: BMJ Open. 2023 Mar 17;13(3):e068117. doi: 10.1136/bmjopen-2022-068117 (PMC10030779; doi:10.1136/bmjopen-2022-068117)
Supplement: Supplementary data [file bmjopen-2022-068117supp004.pdf]

Supplementary Table 1 Characteristics of the study population according to number and percent currently pregnant, 1988 to 2019.

Survey years	1988 - 1998		2003 - 2008		2011 - 2019	
Population analysed (unweighted)	2541		8665		8383	
Number (unweighted) currently pregnant	96		117		282	
Percent (weighted) currently pregnant	3.4%		3.7%		3.7%	
Mean age (SD), years	16.9 (0.03)		16.9 (0.03)		16.9 (0.03)	
	n	%	n	%	n	%
**Age**						
15 - 17	27	1.9	40	2.2	87	1.8
18 - 19	69	5.6	77	6.2	195	7.1
**Place of residence**						
Urban	27	2.6	30	2.2	85	2.6
Rural	69	3.9	87	5.3	197	4.8
**Sex of household head**						
Male	35	3.0	66	3.2	191	3.9
Female	26	2.6	51	4.6	91	3.4
**Number of household members**						
≤4 members	54	5.5	61	5.7	102	4.8
>4 members	42	2.5	56	2.8	180	3.2
**Mother still alive**						
Yes	25	12.9	23	2.2	59	1.8
No	4	33.3	0	0.0	3	1.7
**Father still alive**						
Yes	24	14.0	18	2.0	49	1.7
No	5	15.2	4	2.4	13	2.8
**Literacy level**						
Illiterate	62	6.1	81	8.6	119	8.7
Semi-literate	7	2.8	14	3.2	38	4.7
Literate	27	2.0	21	1.7	68	2.1
**Employment**						
Yes	48	5.9	49	7.3	27	5.9
No	48	2.4	37	2.7	33	2.8
**Household income**						
Low income	26	3.9	71	6.6	178	5.1
Middle income	13	2.2	28	4.4	56	3.8
High income	22	2.3	18	1.5	48	2.2
**Frequency of media exposure**						
Not at all	-	-	15	7.0	70	3.6
Less than once a week	-	-	11	7.7	22	2.6
At least once a week	-	-	58	3.4	114	3.6
**Age at first sex**						
≤14 years	19	6.6	17	9.7	48	7.7

≥15 years	64	7.2	81	9.4	140	8.6
**Number of previous sex partners**						
1	-	-	23	8.2	36	8.2
2	-	-	11	14.8	16	9.8
Three or more	-	-	6	24.0	8	11.3
**Age of last sexual partner**						
Below 20 years	-	-	5	9.3	14	4.7
More than 20 years	-	-	2	1.1	54	7.2
**Knowledge of contraception**						
Knows no method	14	2.7	4	2.5	-	-
Knows modern or traditional method	55	4.9	82	4.2	-	-
**knowledge of fertility period during menstruation**						
Just before or during menstrual period	4	8.1	12	5.4	4	1.3
Right after menstrual period or at any time	18	5.9	42	5.1	27	3.9
Halfway between two menstrual periods	13	6.1	12	3	23	6.8
