# Supplementary material for: Analysis of national and subnational prevalence of adolescent pregnancy and changes in the associated sexual behaviours and sociodemographic determinants across three decades in Ghana, 1988–2019
Source: BMJ Open. 2023 Mar 17;13(3):e068117. doi: 10.1136/bmjopen-2022-068117 (PMC10030779; doi:10.1136/bmjopen-2022-068117)
Supplement: Supplementary data [file bmjopen-2022-068117supp005.pdf]

Supplementary Table 2 Results of unadjusted logistic regression analysis of the association between sociodemographic characteristics and adolescent pregnancy, 1988-2019

Survey years	1988 - 1998	2003 - 2008	2011 - 2019	Overall sample (1988-2019)
Population analysed (n)	2541	3350	8665	14556
Number currently pregnant	96	117	282	495
	**Unadjusted odds ratio (95% CI)**	**Unadjusted odds ratio (95% CI)**	**Unadjusted odds ratio (95% CI)**	**Unadjusted odds ratio (95% CI)**
**Age**	P < 0.001	P < 0.001	P < 0.001	P < 0.001
15 - 17	1.00	1.00	1.00	1.00
18 - 19	3.08 (1.87 - 5.08)	2.95 (1.86 - 4.68)	4.23 (2.98 - 6.00)	3.62 (2.83 - 4.64)
**Place of residence**	P = 0.08	P < 0.001	P < 0.001	P < 0.001
Urban	0.64 (0.39 - 1.06)	0.40 (0.24 - 0.66)	0.53 (0.38 - 0.74)	0.52 (0.41 - 0.66)
Rural	1.00	1.00	1.00	1.00
**Sex of household head**	P = 0.68	P = 0.09	P = 0.37	P = 0.89
Male	1.00	1.00	1.00	1.00
Female	0.87 (0.46 - 1.67)	1.45 (0.94 - 2.25)	0.86 (0.61 - 1.20)	0.98 (0.76 - 1.27)
**Number of household members**	P < 0.001	P < 0.001	P = 0.01	P < 0.001
≤4 members	1.00	1.00	1.00	1.00
>4 members	0.43 (0.28 - 0.67)	0.48 (0.32 - 0.71)	0.65 (0.47 - 0.90)	0.56 (0.45 - 0.69)
**Mother still alive**	P = 0.07		P = 0.95	P = 0.53
Yes	0.30 (0.08 - 1.10)	--	1.04 (0.25 - 4.37)	0.76 (0.33 - 1.77)
No	1.00	--	1.00	1.00
**Father still alive**	P = 0.86	P = 0.74	P = 0.15	P = 0.16
Yes	1.00	1.00	1.00	1.00
No	1.10 (0.37 - 3.24)	1.23 (0.36 - 4.19)	1.69 (0.83 - 3.41)	1.47 (0.87 - 2.48)
**Literacy level**	P < 0.001	P < 0.001	P < 0.001	P < 0.001
Illiterate	1.00	1.00	1.00	1.00
Semi-literate	0.44 (0.18 - 1.07)	0.36 (0.19 - 0.68)	0.52 (0.30 - 0.89)	0.48 (0.33 - 0.70)
Literate	0.31 (0.19 - 0.51)	0.18 (0.11 - 0.29)	0.22 (0.15 - 0.34)	0.24 (0.18 - 0.31)
**Employment**	P < 0.001	P < 0.001	P = 0.02	P < 0.001
Yes	1.00	1.00	1.00	1.00
No	0.40 (0.25 - 0.62)	0.35 (0.21 - 0.58)	0.46 (0.24 - 0.88)	0.39 (0.29 - 0.52)
**Household income**	P = 0.13	P < 0.001	P < 0.001	P < 0.001
Low income	1.00	1.00	1.00	1.00
Middle income	0.56 (0.27 - 1.15)	0.65 (0.41 - 1.03)	0.74 (0.50 - 1.09)	0.70 (0.53 - 0.93)
High income	0.57 (0.31 - 1.05)	0.22 (0.13 - 0.38)	0.42 (0.27 - 0.63)	0.37 (0.27 - 0.50)

**Frequency of media exposure**		P = 0.02	P = 0.50	P = 0.73
Not at all	--	2.10 (1.10 - 4.01)	0.99 (0.65 - 1.52)	1.13 (0.79 - 1.61)
Less than once a week	--	2.33 (0.94 - 5.74)	0.72 (0.41 - 1.26)	0.93 (0.58 - 1.49)
At least once a week	--	1.00	1.00	1.00
