# Supplementary material for: Analysis of national and subnational prevalence of adolescent pregnancy and changes in the associated sexual behaviours and sociodemographic determinants across three decades in Ghana, 1988–2019
Source: BMJ Open. 2023 Mar 17;13(3):e068117. doi: 10.1136/bmjopen-2022-068117 (PMC10030779; doi:10.1136/bmjopen-2022-068117)
Supplement: Supplementary data [file bmjopen-2022-068117supp006.pdf]

Supplementary Table 3 Results of unadjusted logistic regression analysis of the association between sexual behaviours and adolescent pregnancy, 1988-2019.

Survey years	1988 - 1998	2003 - 2008	2011 - 2019	Overall sample (1988-2019)
Population analysed (n)	2541	3350	8665	14556
Number currently pregnant	96	117	282	495
	**Unadjusted odds ratio (95% CI)**	**Unadjusted odds ratio (95% CI)**	**Unadjusted odds ratio (95% CI)**	**Unadjusted odds ratio (95% CI)**
**Age at first sex**	P = 0.79	P = 0.0.91	P = 0.56	P = 0.61
≤14 years	0.92 (0.49 - 1.71)	1.04 (0.53 - 2.03)	0.88 (0.57 - 1.36)	0.92 (0.68 - 1.26)
≥15 years	1.00	1.00	1.00	1.00
**Number of previous sex partners**		P = 0.06	P = 0.72	P = 0.08
1	-	0.28 (0.10 - 0.83)	0.70 (0.28 - 1.75)	0.46 (0.23 - 0.93)
2	-	0.55 (0.20 - 1.53)	0.85 (0.31 - 2.31)	0.69 (0.34 - 1.41)
Three or more	-	1.00	1.00	1.00
**Age of last sexual partner**		P = 0.02	P = 0.26	P = 0.53
Below 20 years	-	1.00	1.00	1.00
More than 20 years	-	0.11 (0.02 - 0.65)	1.56 (0.72 - 3.37)	1.24 (0.63 - 2.45)
**Knowledge of contraception**	P = 0.09	P = 0.34		P = 0.06
Knows no method	0.55 (0.27 - 1.10)	0.57 (0.18 - 1.79)	-	0.57 (0.32 - 1.03)
Knows modern or traditional method	1.00	1.00	-	1.00
**knowledge of fertility period during menstruation**	P = 0.88	P = 0.23		P = 0.78
Just before or during menstrual period	1.35 (0.36 - 5.13)	1.85 (0.74 - 4.61)	-	0.81 (0.44 - 1.52)
Right after menstrual period or at any time	0.97 (0.44 - 2.16)	1.75 (0.90 - 3.39)	-	1.00 (0.67 - 1.49)
Halfway between two menstrual periods	1.00	1.00	-	1.00
