# Supplementary material for: Analysis of national and subnational prevalence of adolescent pregnancy and changes in the associated sexual behaviours and sociodemographic determinants across three decades in Ghana, 1988–2019
Source: BMJ Open. 2023 Mar 17;13(3):e068117. doi: 10.1136/bmjopen-2022-068117 (PMC10030779; doi:10.1136/bmjopen-2022-068117)
Supplement: Author's manuscript [file bmjopen-2022-068117.draft_revisions.pdf]

BMJ Open

BMJ Open is committed to open peer review. As part of this commitment we make the peer review history of every article we publish publicly available.

When an article is published we post the peer reviewers' comments and the authors' responses online. We also post the versions of the paper that were used during peer review. These are the versions that the peer review comments apply to.

The versions of the paper that follow are the versions that were submitted during the peer review process. They are not the versions of record or the final published versions. They should not be cited or distributed as the published version of this manuscript.

BMJ Open is an open access journal and the full, final, typeset and author-corrected version of record of the manuscript is available on our site with no access controls, subscription charges or pay-per-view fees (http://bmjopen.bmj.com).

If you have any questions on BMJ Open's open peer review process please email info.bmjopen@bmj.com

National and subnational prevalence of adolescent pregnancy and changes in the associated sexual behaviours and sociodemographic determinants across three decades in Ghana, 1988 – 2019: an analysis of nationally representative surveys

Journal:	*BMJ Open*
Manuscript ID	bmjopen-2022-068117
Article Type:	Original research
Date Submitted by the Author:	07-Sep-2022
Complete List of Authors:	Mohammed, Shamsudeen; London School of Hygiene & Tropical Medicine, Department of Non-communicable Disease Epidemiology
Keywords:	Epidemiology < INFECTIOUS DISEASES, Public health < INFECTIOUS DISEASES, REPRODUCTIVE MEDICINE

SCHOLARONE™
Manuscripts

I, the Submitting Author has the right to grant and does grant on behalf of all authors of the Work (as defined in the below author licence), an exclusive licence and/or a non-exclusive licence for contributions from authors who are: i) UK Crown employees; ii) where BMJ has agreed a CC-BY licence shall apply, and/or iii) in accordance with the terms applicable for US Federal Government officers or employees acting as part of their official duties; on a worldwide, perpetual, irrevocable, royalty-free basis to BMJ Publishing Group Ltd ("BMJ") its licensees and where the relevant Journal is co-owned by BMJ to the co-owners of the Journal, to publish the Work in this journal and any other BMJ products and to exploit all rights, as set out in our licence.

The Submitting Author accepts and understands that any supply made under these terms is made by BMJ to the Submitting Author unless you are acting as an employee on behalf of your employer or a postgraduate student of an affiliated institution which is paying any applicable article publishing charge ("APC") for Open Access articles. Where the Submitting Author wishes to make the Work available on an Open Access basis (and intends to pay the relevant APC), the terms of reuse of such Open Access shall be governed by a Creative Commons licence – details of these licences and which Creative Commons licence will apply to this Work are set out in our licence referred to above.

Other than as permitted in any relevant BMJ Author's Self Archiving Policies, I confirm this Work has not been accepted for publication elsewhere, is not being considered for publication elsewhere and does not duplicate material already published. I confirm all authors consent to publication of this Work and authorise the granting of this licence.

For peer review only

National and subnational prevalence of adolescent pregnancy and changes in the associated sexual behaviours and sociodemographic determinants across three decades in Ghana, 1988 – 2019: an analysis of nationally representative surveys

Shamsudeen Mohammed [1]

[1]Department of Non-communicable Disease Epidemiology, Faculty of Epidemiology and Population Health, London School of Hygiene & Tropical Medicine, London, UK,

Correspondence: Shamsudeen Mohammed, Department of Non-communicable Disease Epidemiology, London School of Hygiene Tropical Medicine, Keppel Street, London WC1E 7HT Shamsudeen.Mohammed1@lshtm.ac.uk

For peer review only - http://bmjopen.bmj.com/site/about/guidelines.xhtml

Abstract

Objective

The study aimed to examine changes over time in the prevalence of adolescent pregnancy and its associated sexual behaviours and sociodemographic determinants in Ghana.

Methods

Eleven nationally representative cross-sectional surveys from the Ghana Demographic and Health Survey (1988, 1993, 1998, 2003, 2008, 2014), Multiple Indicator Cluster Survey (2006, 2011, 20117-18), and Malaria Indicator Survey (2016 and 2019) provided data on 14556 adolescents aged 15-19 for this analysis. A random-effect meta-analysis, time trends, and multivariable logistic regression models were used to track the prevalence and determinants of adolescent pregnancy across three decades, 1988-1998, 2003-2008, and 2011-2019.

Results

The prevalence of adolescent pregnancy was 15.4% (95% CI=13.49%–17.30%), with substantial regional variation. The pregnancy rates among adolescents in Ghana steadily increased over the past decade after falling between 1988 and 1998. Rural areas (19.5%) had a higher prevalence of adolescent pregnancy than urban areas (10.6%). Few factors associated with adolescent pregnancy remained constant over time. Adolescents had a lower odds of becoming pregnant if they were middle adolescents, literate or semiliterate, lived with five or more household members, came from a high-income household, attained beyond primary level education, had never worked, resided in an urban area, and if they had a sexual relationship with an older person.

Conclusion

The prevalence of adolescent pregnancy increased over the last decade, and girls from disadvantaged social backgrounds were more likely to become pregnant.

Strengths and limitations of this study

The main strength of this study is the use of large nationally representative surveys that captured adolescents from various sociodemographic backgrounds and settings.

The majority of Ghanaian women admit to pregnancy after the first trimester, so it is possible that the prevalence reported in this study was understated.

Due to the study's reliance on secondary data, cultural and traditional practices that may impact adolescent pregnancy were not analysed.

For peer review only - http://bmjopen.bmj.com/site/about/guidelines.xhtml

Background

Globally, fifteen percent of adolescents give birth before their eighteenth birthday(1). In low- and middle-income countries, about 36 million teenagers gave birth in 2010, and if current trends continue, that number is expected to rise to 86 million by 2030 (2). In Africa, a meta-analysis of 52 studies revealed that the prevalence of adolescent pregnancy in the region was higher than that reported in other low- and middle-income countries, with substantial regional variation ranging from 9.2% in North Africa to 21.5% in East Africa (3). The implications of adolescent pregnancy on young women can be devastating. For instance, a girl's educational prospects may suffer if she needs to quit school because she is pregnant. Further, teenage girls who become pregnant risk many negative social repercussions, including isolation, stigma, rejection from peers and family, and even forced marriage(1). In many low- and middle-income settings, adolescents who become pregnant, face an increased risk of unsafe abortion, eclampsia, obstruction during childbirth, obstetric fistula, and maternal death and disability because their reproductive organs may not be fully matured (4–9). The risk of maternal death among girls between the ages of 15 and 19 is double that of women over the age of 20(10).

Many epidemiological studies have shown a considerable disparity in the drivers of adolescent pregnancy, where disadvantaged adolescents are more at risk of pregnancy than those better-off. For example, child marriage, non-school attendance, poverty, living in rural residence, household living arrangement, no maternal education, no paternal education, history of maternal teenage pregnancy, cost of contraceptives, misconceptions about contraceptives, and lack of parental counselling and guidance on sexual and reproductive health issues are the main sociodemographic and economic factors that drive adolescent pregnancy in Africa(3,11,12). Furthermore, sexual risk factors, including unwanted sexual advances from adult males, coercive sexual relations, unequal gender power relations, lack of comprehensive sexuality education, and early sexual debut, also increase adolescents susceptibility to pregnancy(3,9,11,12).

In Ghana, not many studies have explored the predictors of adolescent pregnancy, despite the need for country-specific estimates and determinants to inform national policies and programmes on adolescent pregnancy and interventions targeted at improving the lives of adolescents. Moreover, the few studies conducted over the last few years had small samples and reported contradictory findings(13,14). The present study used data from eleven nationally representative surveys conducted between 1988 and 2019 to estimate the national and regional prevalence of adolescent pregnancy and track changes in the sociodemographic and sexual risk behaviours associated with adolescent pregnancy across three decades.

Methods

Eleven nationally representative cross-sectional surveys from the Ghana Demographic and Health Survey (1988, 1993, 1998, 2003, 2008, 2014), Multiple Indicator Cluster Survey (2006, 2011, 20117-18), and Malaria Indicator Survey (2016 and 2019) provided the data for this analysis. A detailed description of the methods used in the surveys, including information about survey design, sampling, and data collection, is published elsewhere(15). Briefly, the surveys used a multistage stratified cluster sampling method to select households from rural and urban clusters in Ghana's former ten administrative regions (now 16 regions). The participant questionnaires administered in these surveys are comparable, allowing data pooling across surveys and time. For all the surveys, trained field officers under the supervision of the Ghana Statistical Services administered the questionnaires face-to-face to women, men, and children in

For peer review only - http://bmjopen.bmj.com/site/about/guidelines.xhtml

selected households. The response rate in the surveys was higher than 90%. Data on women aged 15 to 19 years were extracted from data files of the eleven surveys for the present analysis.

Variables

The outcome of interest for this study was adolescent pregnancy. It was defined as women aged 15 to 19 years who had a birth or were pregnant at the time of the interview. Across the surveys, the same questions were asked to elicit responses to measure the outcome. Women were asked *"are you pregnant now?"* to determine those pregnant at the time of the survey and *"have you ever given birth?"* to ascertain those who have ever given birth. Adolescents sociodemographic and sexual risk behaviours were explored as potential explanatory factors of adolescent pregnancy. The sociodemographic factors included adolescent age, place of residence, sex of household head, number of household members, mother still alive, father still alive, literacy level, employment status, household income, and frequency of media exposure (newspaper + radio + TV). The sexual behaviours that were considered in the analysis are adolescents age at first sex, number of previous sex partners, age of last sexual partner, knowledge of contraception, knowledge of fertility period during menstruation, whether adolescent can refuse sex, and whether wife-beating is justified if she refuses to have sex with husband. The explanatory variables were not available for all surveys.

Data management and statistical analysis

Data from each survey year were standardised and pooled into a single dataset before being grouped into three periods, 1988-1998, 2003-2008, and 2011-2019. The prevalence of adolescent pregnancy was estimated as a percentage of all adolescents aged 15 to 19 years for each survey year, the three eras, and the overall sample. Time trends of adolescent pregnancy and age patterns were plotted for survey years and selected sociodemographic factors. Random effect meta-analysis was used to pool the prevalence across the survey years to estimate the prevalence of adolescent pregnancy in Ghana. The pooled prevalence and corresponding 95% confidence intervals were presented using forest plots. Heterogeneity among the surveys was assessed using Higgins and Thompson's I^2 statistic(16). Subgroup analyses were performed to assess variations in the prevalence across the administrative regions of Ghana and rural-urban residence. To scale prevalence estimates to represent all adolescents in Ghana, denormalised survey weights were applied to correct for unequal sampling in certain areas and subgroups.

Multivariable logistic regression was used to determine the factors associated with adolescent pregnancy across the three decades (1988-1998, 2003-2008, and 2011-2019) and the overall sample. Potential determinants were categorised into distal (sociodemographic factors) and proximal (sexual behaviours) factors, and a conceptual framework was used to guide the analysis (Supplementary Table 1). Based on the framework, distal factors were considered potential confounders of the association between the proximal factors and the outcome in the multivariable model. The logistic regression models accounted for stratification in the survey design, the year surveys were conducted, and calculated robust standard errors to account for clustering in the design of the surveys. To minimise the influence of reverse causation and recall bias, the logistic regression analysis was restricted to adolescents who were pregnant at the time of the survey. Because this study is exploratory rather than confirmatory, P<0.10 was used to determine statistically significant associations. A test for linear trend was performed for literacy level and household income using Stata postestimation command, *contrast*. Estimates for explanatory variables not available in all the surveys were produced in a separate multivariable model. Stata version 17 was used for all data management and analysis.

For peer review only - http://bmjopen.bmj.com/site/about/guidelines.xhtml

Ethics approval

Ethical approval was not required for this study

Patient and public involvement

Patients or the public were not involved in the design, conduct, reporting, or dissemination plans of our research

Results

Characteristics of the study sample

A nationally representative sample of 14556 adolescents aged 15 to 19 years from surveys conducted between 1988 and 2019 was included in the analysis. Table 1 presents the characteristics of the total study sample. Overall, most adolescents lived in rural areas, in households with high income, headed by men, and with four or more household members. The majority of the sample were middle-aged adolescents aged 15 to 17 years. Only a small percentage of the adolescents were orphans, ever consumed alcohol, or worked at some point in the 12 months prior to the surveys. More than half of the adolescents could read a whole sentence, but only 18.3% had attained secondary or post-secondary education; most had only attained junior high school. Although many adolescents were exposed to mainstream media at least once a week (radio, television, or newspaper), few owned a mobile phone or had internet access. Approximately half of them initiated sex after age 15, and only 26.2% wore a condom for their first sex. The majority of them had had only one sexual relationship, indicated they could decline sex from a partner, were aware of modern or traditional contraceptive methods, and agreed that a wife should not be beaten for refusing sex with her spouse. For most adolescents in this sample, their last sexual partner was an adult over age 20.

Table 1 Characteristics of the study population, 1988-2019

Survey years	Total sample (1988 - 2019)	
Population analysed (n)	14556	
Mean age (SD), years	16.9 (0.01)	
	n	%
**Age**		
15 - 17	8986	62.4
18 - 19	5570	37.6
**Place of residence**		
Urban	6254	47.1
Rural	8302	52.9
**Sex of household head**		
Male	8718	59.9
Female	4989	40.1
**Number of household members**		
1 - 4 members	4209	30.8
More than 4 members	10347	69.2

For peer review only - http://bmjopen.bmj.com/site/about/guidelines.xhtml

For peer review only

**Mother still alive**		
Yes	5454	94.2
No	309	5.8
**Father still alive**		
Yes	4965	86.1
No	790	13.9
**Ever drunk alcohol**		
Yes	581	18.8
No	2393	81.2
**Literacy level**		
Cannot read at all	3540	27.9
Able to read only partially sentence	1932	14.1
Able to read whole sentence	6238	58.1
**Employment**		
Yes	1985	30.3
No	4442	69.7
**Household income**		
Low income	6268	36.9
Middle income	2561	21.2
High income	4878	41.9
**Education level**		
No education	1170	6.8
Primary school	2884	19.3
Junior high school	7339	55.7
Senior high or post-secondary school	2314	18.3
**Frequency of media exposure**		
Not at all	2483	22.1
Less than once a week	1282	14.8
At least once a week	5134	63.1
**Own a mobile phone**		
Yes	1081	37.5
No	1893	62.5
**Has access to the internet in any device**		
Yes	170	11.5
No	1552	88.5
**Age at first sex**		
Before age 16	2339	49.7
16 - 19 years	2416	50.3
**Condom used at first sexual intercourse**		
Yes	100	26.2
No	285	73.8
**Number of previous sex partners**		
1	764	66.7
2	243	23.8

For peer review only - http://bmjopen.bmj.com/site/about/guidelines.xhtml

For peer review only

Three or more	106	9.4
**Age of last sexual partner**		
Below 20 years	381	28.4
More than 20 years	993	71.6
**Knowledge of contraception**		
Knows no method	666	14.1
Knows modern or traditional method	4892	85.9
**knowledge of fertility period during menstruation**		
Just before or during menstrual period	546	14.6
Right after menstrual period or at any time	1964	52.1
Halfway between two menstrual periods	1067	33.3
**Can refuse sex**		
Yes	162	75.3
No	49	24.7
**Wife beating justified if she refuses to have sex with him**		
Yes	1645	14.4
No	8096	85.6

Prevalence and time trends of adolescent pregnancy from 1988 - 2019

The prevalence of adolescent pregnancy was 15.4% (95%CI=13.49% – 17.30%) with significant variation across the eleven surveys (I^2 = 89.9%, p < 0.001; figure 1). There was a substantial regional disparity in the prevalence, ranging from 8.2% (95%CI=6.29%-10.39%) in the Greater Accra region to 19.3% (95% CI=15.75%-23.19%) in the Central region (Table 2; see Supplementary Figure 1 for details). Table 2 shows that the prevalence of adolescent pregnancy in rural areas (19.5%) was nearly twice that of urban areas (10.6%) (see Supplementary Figure 2 for details). The prevalence dropped between 1988 and 1998, then remained relatively consistent from 2003 to 2008 before gradually increasing in the last decade (figure 2A). Adolescents from high-income households and those who could read an entire sentence consistently had a lower prevalence of adolescent pregnancy throughout the eleven surveys, with the steepest decline seen between 1993 and 2006(Figures 2B and 2C). There was an upward trend of adolescent pregnancy with increasing age across the surveys (figure 2D).

Table 2 Subnational prevalence of adolescent pregnancy based on meta-analysis of nationally representative surveys from 1988 to 2019.

	Prevalence (95% confidence interval)
**Region**	
Western region	14.42 (11.27 - 17.87)
Central region	19.34 (15.75 - 23.19)
Greater Accra region	8.23 (6.29 - 10.39)
Volta region	16.18 (13.50 - 19.05)
Eastern region	16.20 (13.93 - 18.61)
Ashanti region	14.14 (11.94 - 16.48)
Brong-Ahafo region	16.73 (12.66 - 21.22)
Northern region	16.41 (11.62 - 21.82)
Upper West region	11.48 (8.48 - 14.82)

For peer review only - http://bmjopen.bmj.com/site/about/guidelines.xhtml

Upper East region	11.40 (8.73 - 14.36)
**Residence**	
Urban	10.59 (8.69 - 12.66)
Rural	19.50 (17.51 - 21.57)

Sexual behaviours and sociodemographic factors associated with adolescent pregnancy

Table 3 summarises the adolescents sexual behaviour and sociodemographic characteristics stratified by the number and percent who were pregnant at the time of the survey. Unadjusted sociodemographic factors and sexual behaviours associated with adolescent pregnancy in the three eras studied are presented in Supplementary Table 2 and Table 3.

After adjusting for other sociodemographic variables, adolescents age, household size, mother being alive, literacy level, and employment status were the factors associated with adolescent pregnancy between 1988 and 1998 (Table 4). Middle adolescents were less likely to become pregnant than those in their late adolescence. Adolescents in households of five or more members were less likely to become pregnant than those in households of four or fewer members. Those whose mothers were no longer alive were about three times more likely than those whose mothers were still alive to become pregnant. Adolescents who could read a portion of a sentence or a whole sentence were less likely than those who could not read at all to become pregnant. Those who had not worked in the previous 12 months were 50% less likely than those who had worked to become pregnant.

Between 2003 and 2008, adolescents age, household size, literacy level, household income level, age of last sexual partner, and knowledge of contraception were the factors associated with adolescent pregnancy after adjusting for other sexual behaviours and sociodemographic factors. Adolescents in high-income families were 68% less likely than those in low-income families to become pregnant. Middle adolescents were less likely to become pregnant than those in their late adolescence. Adolescents in households of five or more members were less likely to become pregnant than those living in households of four or fewer members. Those who could read a portion of a sentence or a whole sentence were less likely to become pregnant than those who could not read at all (Table 4). Those whose last sexual partner was an older person were less likely to become pregnant than those whose last partner was an adolescent. Adolescents who did not know any contraceptive methods were less likely than those who were aware of modern or traditional methods to become pregnant, albeit with wider confidence intervals (Table 5).

From 2011 to 2019, adolescent age, place of residence, literacy level, and knowledge of fertility period during menstruation were the factors associated with adolescent pregnancy after adjusting for other sexual behaviours and sociodemographic factors. Middle adolescents were less likely to become pregnant than those in their late adolescence. Adolescents in urban residents were about 50% less likely than those in rural residences to become pregnant. Those who could read a portion of a sentence or a whole sentence were less likely to become pregnant compared to those who could not read at all (Table 4). Adolescents who indicated that the most fertile time was immediately before the menstrual period were less likely than those who said halfway between two menstrual periods to become pregnant (Table 5).

For peer review only - http://bmjopen.bmj.com/site/about/guidelines.xhtml

Table 3 Characteristics of the study population stratified by number and percent currently pregnant, 1988 to 2019.

Survey years	1988 - 1998		2003 - 2008		2011 - 2019	
Population analysed (n)	2541		8665		8383	
Number currently pregnant	96		117		282	
Percent currently pregnant	3.4%		3.7%		3.7%	
Mean age (SD), years	16.9 (0.03)		16.9 (0.03)		16.9 (0.03)	
	n	w-%	n	w-%	n	w-%
**Age**						
15 - 17	27	1.9	40	2.2	87	1.8
18 - 19	69	5.6	77	6.2	195	7.1
**Place of residence**						
Urban	27	2.6	30	2.2	85	2.6
Rural	69	3.9	87	5.3	197	4.8
**Sex of household head**						
Male	35	3.0	66	3.2	191	3.9
Female	26	2.6	51	4.6	91	3.4
**Number of household members**						
1 - 4 members	54	5.5	61	5.7	102	4.8
More than 4 members	42	2.5	56	2.8	180	3.2
**Mother still alive**						
Yes	25	12.9	23	2.2	59	1.8
No	4	33.3	0	0.0	3	1.7
**Father still alive**						
Yes	24	14.0	18	2.0	49	1.7
No	5	15.2	4	2.4	13	2.8
**Ever drunk alcohol**						
Yes	-	-	-	-	24	5.5
No	-	-	-	-	59	3.0
**Literacy level**						
Cannot read at all	62	6.1	81	8.6	119	8.7
Able to read only partially sentence	7	2.8	14	3.2	38	4.7
Able to read whole sentence	27	2.0	21	1.7	68	2.1
**Employment**						
Yes	48	5.9	49	7.3	27	5.9
No	48	2.4	37	2.7	33	2.8
**Household income**						
Low income	26	3.9	71	6.6	178	5.1
Middle income	13	2.2	28	4.4	56	3.8
High income	22	2.3	18	1.5	48	2.2
**Education level**						
No education	285	6.0	372	7.3	430	5.5
Primary school	273	5.1	789	6.7	1664	6.6

For peer review only

For peer review only - http://bmjopen.bmj.com/site/about/guidelines.xhtml

Junior high school	901	1.7	1572	3.0	4665	3.7
Senior high or post-secondary school	172	0.5	500	0.1	1624	0.9
**Frequency of media exposure**						
Not at all	-	-	15	7.0	70	3.6
Less than once a week	-	-	11	7.7	22	2.6
At least once a week	-	-	58	3.4	114	3.6
**Own a mobile phone**						
Yes	-	-	-	-	36	3.5
No	-	-	-	-	47	3.4
**Has access to the internet in any device**						
Yes	-	-	-	-	2	0.9
No	-	-	-	-	57	4.2
**Age at first sex**						
Before age 16	38	6.5	46	10.6	99	9.1
16 - 19 years	45	7.6	52	8.6	89	7.6
**Condom used at first sexual intercourse**						
Yes	-	-	8	8.6	-	-
No	-	-	32	11.8	-	-
**Number of previous sex partners**						
1	-	-	23	8.2	36	8.2
2	-	-	11	14.8	16	9.8
Three or more	-	-	6	24.0	8	11.3
**Age of last sexual partner**						
Below 20 years	-	-	5	9.3	14	4.7
More than 20 years	-	-	2	1.1	54	7.2
**Knowledge of contraception**						
Knows no method	14	2.7	4	2.5	-	-
Knows modern or traditional method	55	4.9	82	4.2	-	-
**knowledge of fertility period during menstruation**						
Just before or during menstrual period	4	8.1	12	5.4	4	1.3
Right after menstrual period or at any time	18	5.9	42	5.1	27	3.9
Halfway between two menstrual periods	13	6.1	12	3	23	6.8
**Can refuse sex**	-	-				
Yes	-	-	20	31.3	17	22.9
No			6	26.9	4	12.0
**Wife beating justified if she refuses to have sex with him**						
Yes	-	-	25	4.0	41	4.1
No	-	-	91	3.9	163	3.4

n= Unweighted number of adolescents in each category.

w-% Weighted percentage of adolescents in each category

For peer review only

For peer review only - http://bmjopen.bmj.com/site/about/guidelines.xhtml

Table 4 Adjusted analysis of the sociodemographic factors associated with adolescent pregnancy, 1988-2019.

Survey years	1988 - 1998	2003 - 2008	2011 - 2019	Overall sample (1988-2019)
Population analysed (n)	2541	3350	8665	14556
Number currently pregnant	96	117	282	495
	**Adjusted odds ratio (95% CI)**	**Adjusted odds ratio (95% CI)**	**Adjusted odds ratio (95% CI)**	**Adjusted odds ratio (95% CI)**
**Age**	**P < 0.001**	**P < 0.001**	**P < 0.001**	**P < 0.001**
15 - 17	**0.40 (0.24 - 0.66)**	**0.39 (0.24 - 0.64)**	**0.22 (0.15 - 0.33)**	**0.30 (0.23 - 0.39)**
18 - 19	**1.00**	**1.00**	**1.00**	**1.00**
**Place of residence**	P = 0.23	P = 0.72	**P = 0.002**	**P < 0.001**
Urban	0.74 (0.45 - 1.22)	0.86 (0.40 - 1.89)	**0.54 (0.36 - 0.81)**	**0.56 (0.43 - 0.74)**
Rural	1.00	1.00	**1.00**	**1.00**
**Sex of household head**	P = 0.23	P = 0.06	P = 0.60	P = 0.42
Male	1.61 (0.73 - 3.54)	0.61 (0.36 - 1.02)	0.91 (0.63 - 1.31)	0.89 (0.66 - 1.19)
Female	1.00	1.00	1.00	1.00
**Number of household members**	**P = 0.001**	**P = 0.02**	P = 0.19	**P < 0.001**
1 - 4 members	**1.00**	**1.00**	1.00	**1.00**
More than 4 members	**0.46 (0.29 - 0.73)**	**0.58 (0.37 - 0.91)**	0.78 (0.54 - 1.13)	**0.62 (0.49 - 0.78)**
**Mother still alive**	P = 0.07		P = 0.48	P = 0.94
Yes	0.30 (0.08 - 1.09)	-	0.58 (0.13 - 2.58)	0.94 (0.24 - 3.77)
No	1.00	-	1.00	1.00
**Father still alive**	P = 0.85	P = 0.86	P = 0.99	P = 0.84
Yes	1.00	1.00	1.00	1.00
No	1.12 (0.36 - 3.44)	1.13 (0.30 - 4.21)	1.00 (0.40 - 2.51)	1.08 (0.52 - 2.24)
**Literacy level**	**P = 0.001***	**P < 0.001***	**P < 0.001***	**P < 0.001***
Cannot read at all	**1.00**	**1.00**	**1.00**	**1.00**
Able to read only partially sentence	**0.55 (0.20 - 1.50)**	**0.42 (0.22 - 0.81)**	**0.66 (0.39 - 1.13)**	**0.56 (0.39 - 0.82)**
Able to read whole sentence	**0.40 (0.23 - 0.69)**	**0.27 (0.16 - 0.45)**	**0.27 (0.17 - 0.42)**	**0.28 (0.21 - 0.37)**
**Employment**	**P = 0.05**	P = 0.29	P = 0.12	P = 0.01
Yes	**1.00**	1.00	1.00	1.00
No	**0.61 (0.37 - 1.01)**	0.73 (0.41 - 1.31)	0.59 (0.30 - 1.15)	0.62 (0.43 - 0.90)
**Household income**	P = 0.56	**P < 0.001***	P = 0.27	P = 0.08
Low income	1.00	**1.00**	1.00	1.00
Middle income	0.67 (0.32 - 1.39)	**0.65 (0.39 - 1.09)**	1.09 (0.70 - 1.69)	0.91 (0.67 - 1.24)

For peer review only

For peer review only - http://bmjopen.bmj.com/site/about/guidelines.xhtml

High income	0.88 (0.45 - 1.74)	**0.30 (0.15 - 0.58)**	0.63 (0.32 - 1.26)	0.59 (0.36 - 0.94)
**Frequency of media exposure**		P = 0.82	P = 0.23	P = 0.51
Not at all	-	1.00	1.00	1.00
Less than once a week	-	1.40 (0.45 - 4.37)	1.29 (0.62 - 2.69)	1.25 (0.68 - 2.32)
At least once a week	-	1.04 (0.49 - 2.20)	1.70 (0.91 - 3.18)	1.34 (0.82 - 2.20)
**Education level**	**P = 0.003***	**P < 0.001***	**P < 0.001***	**P < 0.001***
No education	**1.00**	**1.00**	**1.00**	**1.00**
Primary school	**0.67 (0.33 - 1.35)**	**1.15 (0.66 - 2.00)**	**1.94 (0.99 - 3.81)**	**1.40 (0.98 - 2.00)**
Junior high school	**0.32 (0.17 - 0.58)**	**0.53 (0.30 - 0.93)**	**0.92 (0.50 - 1.67)**	**0.67 (0.48 - 0.93)**
Senior high or post-secondary school	**0.06 (0.01 - 0.44)**	**0.02 (0.00 - 0.15)**	**0.14 (0.06 - 0.32)**	**0.10 (0.05 - 0.20)**

*P-value for trend

For peer review only

Table 5 Adjusted analysis of the sexual behaviours associated with adolescent pregnancy, 1988-2019.

Survey years	1988 - 1998	2003 - 2008	2011 - 2019	Overall sample (1988-2019)
Population analysed (n)	2541	3350	8665	14556
Number currently pregnant	96	117	282	495
	**Adjusted odds ratio (95% CI)**	**Adjusted odds ratio (95% CI)**	**Adjusted odds ratio (95% CI)**	**Adjusted odds ratio (95% CI)**
**Age at first sex**	P = 0.29	P = 0.72	P = 0.26	P = 0.32
Before age 16	0.76 (0.45 - 1.27)	1.12 (0.61 - 2.04)	0.77 (0.49 - 1.22)	0.86 (0.63 - 1.16)
16 - 19 years	1.00	1.00	1.00	1.00
**Number of previous sex partners**		P = 0.09	P = 0.79	P = 0.10
1	-	0.29 (0.10 - 0.88)	0.73 (0.27 - 1.95)	0.46 (0.23 - 0.95)
2	-	0.56 (0.21 - 1.52)	0.90 (0.31 - 2.58)	0.67 (0.33 - 1.36)
Three or more	-	1.00	1.00	1.00
**Age of last sexual partner**		**P = 0.02**	P = 0.20	P = 0.39
Below 20 years	-	**1.00**	1.00	1.00
More than 20 years	-	**0.14 (0.03 - 0.76)**	2.00 (0.69 - 5.81)	1.46 (0.61 - 3.48)
**Knowledge of contraception**	P = 0.20	P = 0.08	-	**P = 0.02**
Knows no method	0.62 (0.30 - 1.29)	0.36 (0.11 - 1.15)	-	**0.49 (0.27 - 0.90)**
Knows modern or traditional emthod	1.00	1.00	-	**1.00**
**knowledge of fertility period during menstruation**	P = 0.56	P = 0.45	**P = 0.02**	P = 0.83
Just before or during menstrual period	2.09 (0.50 - 8.67)	1.88 (0.70 - 5.05)	**0.17 (0.04 - 0.69)**	0.90 (0.45 - 1.77)
Right after menstrual period or at any time	0.95 (0.42 - 2.13)	1.24 (0.60 - 2.56)	**0.51 (0.24 - 1.07)**	0.87 (0.56 - 1.36)
Halfway between two menstrual periods	1.00	1.00	**1.00**	1.00
**Can refuse sex**	-	P = 0.55	P = 0.45	P = 0.72
Yes	-	1.00	1.00	1.00
No		1.57 (0.34 - 7.19)	0.49 (0.08 - 3.13)	0.84 (0.34 - 2.11)
**Wife beating justified if she refuses to have sex with him**		P = 0.42	P = 0.66	P = 0.35
Yes	-	0.79 (0.45 - 1.40)	0.89 (0.52 - 1.51)	0.83 (0.57 - 1.22)
No	-	1.00	1.00	1.00

Discussion

Based on a meta-analysis of eleven nationally representative surveys conducted between 1988 and 2019, the prevalence of adolescent pregnancy was 15.4%, with substantial regional variation. The prevalence was higher in rural areas than urban areas. Over the last three decades, adolescent pregnancy peaked in 1988, followed by a progressive decline in the 1990s and early 2000s; but the prevalence has slowly

For peer review only - http://bmjopen.bmj.com/site/about/guidelines.xhtml

increased over the last decade. The pooled data suggest that the prevalence of adolescent pregnancy rose with increasing adolescent age, a pattern that was consistent across all eleven surveys.

Over time, most of the factors associated with adolescent pregnancy changed, but a few stayed consistent. For instance, throughout the period 1988 to 2019, middle-aged adolescents, those who could read at least part of a sentence, and those who could read a complete sentence were less likely to become pregnant. However, living with five or more household members was associated with lower odds of adolescent pregnancy from 1988 to 2008, and though a similar pattern was seen between 2011 and 2019, the confidence interval, in this case, was wider. Between 1988 and 1998, adolescents who had never worked were less likely to get pregnant. Household income was inversely associated with adolescent pregnancy between 2003 and 2008; higher household income was associated with lower odds of adolescent pregnancy. There was no link between income and adolescent pregnancy in the other eras. Having a sexual relationship with an older person was associated with lower odds of adolescent pregnancy between 2003 and 2008. From 2011 to 2019, living in an urban area and reporting the fertile phase of the menstrual cycle to be immediately before menstruation were associated with a lower likelihood of adolescent pregnancy.

The prevalence of adolescent pregnancy in this study is lower than the 18.8%, 19.3%, and 17.7% prevalence estimates for Africa, sub-Saharan Africa, and West Africa, respectively (3). In line with the present findings, studies in Indonesia and Uganda reported a higher likelihood of pregnancy among teenagers living in rural areas, those with no or low education, and adolescents in low-income households(17,18). Similarly, in a systematic review of 24 studies in sub-Saharan Africa, about half of the included studies reported a higher likelihood of adolescent pregnancy in low-income households(11). Furthermore, an analysis of the 2014–2015 Rwanda Demographic and Health Survey confirmed that adolescents in large families were less likely than those from small families to get pregnant(19). Similar to the findings of this study, Jonas et al., found higher odds of adolescent pregnancy among those with more than one sex partner in South Africa(20). Though there was no association between adolescent age and pregnancy in the south African study, in a pooled analysis of data from five African countries and two cross-sectional studies in Ghana, older adolescents had higher odds of adolescent pregnancy (6,13,21). In support of the present findings, a qualitative study in Kenya found that adolescents engaged in incoming generating activities were more likely to get pregnant than those not working(22); however, a study in Ethiopia reported a contrary finding(23). In an earlier study in Ghana, there was no association between care status (orphanhood) and adolescent pregnancy(21), which agrees with the results for 2011-2019 and the overall sample.

There are several reasons to explain the heightened vulnerability of adolescents in low-income households to pregnancy. In most cases, adolescents in these homes are not in school; instead, parents force them into early marriage to relieve the financial burden of taking care of the adolescents or prevent out-of-wedlock pregnancy(24). Several studies have revealed that most adolescent girls from poor homes engage in early sexual relationships for financial and material gains because parents cannot provide their basic needs, including food, toiletries, clothes, shoes, school supplies, fees, and housing (22,25–28). For instance, In a Ugandan study, adolescents acknowledged that they sometimes offer sex to boys/men as payment for money borrowed or in return for material items because their parents cannot provide(28). Under these situations, adolescents are less able to negotiate safe sex and condom use, increasing their chance of pregnancy. Some may engage multiple sexual partners for greater financial benefits. Therefore,

For peer review only - http://bmjopen.bmj.com/site/about/guidelines.xhtml

it is not surprising that in this present study, adolescents who had had three or more sexual partners were more likely to conceive pregnancy than those with one partner.

Additionally, some adolescents engage in all manner of income-generating activities to meet their basic needs, and this exposes them to mistreatment, sexual abuse, and exploitation, particularly those working as domestic help, bartenders, waitresses, shopkeepers, and selling at night or work that involves late-night shifts(22,28). This is probably why adolescents who worked 12 months prior to the surveys analysed in this study had a higher chance of becoming pregnant. Also, it has been suggested that some adolescents spend the income from these jobs on activities that increase their risk of pregnancy, such as alcohol, drugs, night clubs(22). In large families, adolescents are supervised, guided, and supported emotionally and financially not only by their parents but also by other family members to make sexual and reproductive health decisions favouring pregnancy prevention, reducing the likelihood of adolescent pregnancy in such families as reported in this study. Also, in these families' adolescents who are uncomfortable discussing sexual health issues with their parents have other family members to consult.

The lower likelihood of pregnancy among adolescents in urban areas may be due to the advantages the urban setting offers. Urban residents have easy access to pregnancy prevention information and services, including easy access to various effective modern contraceptive methods (including emergency contraceptives) through the many hospitals, clinics, pharmacy shops, and family planning centres in urban areas. In rural areas, misconceptions are promoted, even when contraceptives are available, and sexually active adolescents usually rely on traditional contraceptive methods that are ineffective. Furthermore, traditional and cultural practices that promote early adolescent marriage are rarely practised in most urban settings, reducing adolescents' exposure to pregnancy. In addition, most adolescents in urban areas are better educated than their rural counterparts, increasing their ability to understand and effectively use sexual and reproductive health information to reduce their susceptibility to pregnancy. This further explains why adolescents who could read were less likely to conceive pregnancy than those who could not read in this study. Also, schooling delays marriage and empowers girls to make rational decisions in their relationships that prevent pregnancy(9).

Most adolescents in the present study did not know the fertile phase of the menstrual cycle. Therefore, it is surprising that those who did not know the fertile period were less likely to become pregnant. It is possible that these girls had not initiated sex or that those who correctly identified the fertile phase learned it when they became pregnant (reverse causality). This may also explain the unexpected finding on knowledge about contraceptives. However, it was not surprising that older adolescents were more likely to get pregnant than younger adolescents because most older adolescents are sexually active and engage in more sexual risk behaviours than middle-aged adolescents. In addition, the lower adolescent pregnancy among those whose last partner was older is possible due to better pregnancy prevention and sexual and reproductive health knowledge of older partners.

Age-appropriate sexual and reproductive health education as part of the school curriculum and in communities is essential to help adolescents from all settings, income groups, and family backgrounds make rational decisions that will delay pregnancy and motherhood. Adolescents access and use of contraceptives, including emergency contraceptives, should be promoted, particularly in rural communities and low-income households. Traditional leaders and stakeholders who promote cultural and traditional practices that encourage early motherhood should be educated on the harmful effects of adolescent childbearing and such practices discouraged. Girls should be encouraged and motivated to

For peer review only - http://bmjopen.bmj.com/site/about/guidelines.xhtml

stay in school even when they become pregnant, considering the long-term repercussions of dropping out. In addition, interventions to reduce adolescent pregnancy should include boys and men.

The main strength of this study is the use of a large nationally representative sample that captures adolescents from various sociodemographic backgrounds and settings. The study's findings are generalisable to all adolescents in Ghana and more relevant to national policies than previous studies that mainly focused on individual communities. One major limitation of the study is the possibility of reverse causality and recall bias. To minimise the effect of reverse causality, analysis of the determinants was restricted to currently pregnant adolescents. However, it is important to note that this study was not designed for causal relationships, so I make no such claims. Furthermore, it is possible that the prevalence reported in this study was underestimated because most Ghanaian women admit to pregnancy after the first trimester. In addition, because the study relied on secondary data, cultural and traditional practices that could potentially impact adolescent pregnancy were not considered in the analysis because data were not available on these practices. Future studies should address the limitations with longitudinal and qualitative studies.

Conclusion

Adolescent pregnancy has risen slowly over the last decade, following a steady prevalence between 1998 and 2008. Regional and urban-rural disparities in the prevalence were substantial, and age patterns showed that adolescent pregnancy increased as adolescents got older. The factors influencing adolescent pregnancy differed across the eras except for literacy level and adolescent age; illiteracy and older adolescent age were associated with a higher likelihood of adolescent pregnancy across the three decades from 1988 to 2019.

Ethics approval and consent to participate: Ethical approval was not required for this secondary analysis.

Consent for publication: Not applicable

Data sharing statement: All the datasets analysed are publicly available with the measure DHS program (https://dhsprogram.com) and UNICEF's MICS programme (https://mics.unicef.org/).

Competing interests: None

Funding: The author is receiving PhD studentship from the Economic and Social Research Council (ESRC) for doctoral research and training at the London School of Hygiene & Tropical Medicine, London, UK [grant number: ES/P000592/1]. The ESRC had no role in this study's design, analysis of the data, or manuscript preparation.

Authors' contributions: SM conceived and designed the study, accessed the data, conducted the literature search, performed the statistical analysis, and drafted the manuscript.

Acknowledgements: I would like to thank measure DHS and UNICEF's MICS programme for prompt approval and access to the datasets for the analysis.

For peer review only - http://bmjopen.bmj.com/site/about/guidelines.xhtml

For peer review only

References

1. Unicef. Early Childbearing [Internet]. 2021 [cited 2022 Sep 6]. Available from: https://data.unicef.org/topic/child-health/adolescent-health/

2. UNFPA. ADOLESCENT PREGNANCY : A Review of the Evidence [Internet]. New York, USA; 2013. Available from: https://www.unfpa.org/adolescent-pregnancy

3. Kassa GM, Arowojolu AO, Odukogbe AA, Yalew AW. Prevalence and determinants of adolescent pregnancy in Africa: A systematic review and Meta-analysis 11 Medical and Health Sciences 1117 Public Health and Health Services. Reprod Health. 2018;15(1):1–17.

4. UNFPA. Girlhood, not motherhood: Preventing adolescent pregnancy [Internet]. United Nations Population Fund. New York, USA; 2015. Available from: https://www.unfpa.org/sites/default/files/pub-pdf/Girlhood_not_motherhood_final_web.pdf

5. Yussif AS, Lassey A, Ganyaglo GYK, Kantelhardt EJ, Kielstein H. The long-term effects of adolescent pregnancies in a community in Northern Ghana on subsequent pregnancies and births of the young mothers. Reprod Health. 2017;14(1):1–7.

6. Tetteh J, Nuertey BD, Dwomoh D, Udofia EA, Mohammed S, Adjei-Mensah E, et al. Teenage pregnancy and experience of physical violence among women aged 15-19 years in five African countries: Analysis of complex survey data. PLoS One [Internet]. 2020;15(10 October):1–18. Available from: http://dx.doi.org/10.1371/journal.pone.0241348

7. Dzotsi HT, Oppong Asante K, Osafo J. Challenges associated with teenage motherhood in Ghana: a qualitative study. Vulnerable Child Youth Stud [Internet]. 2020;15(1):85–96. Available from: https://doi.org/10.1080/17450128.2020.1719251

8. Gbogbo S. Early motherhood: voices from female adolescents in the Hohoe Municipality, Ghana—a qualitative study utilizing Schlossberg's Transition Theory. Int J Qual Stud Health Well-being [Internet]. 2020;15(1). Available from: https://doi.org/10.1080/17482631.2020.1716620

9. UNFPA. Adolescent pregnancy: A matter of human rights [Internet]. 2017 [cited 2022 Jan 2]. Available from: https://www.unfpa.org/adolescent-pregnancy#readmore-expand

10. UNFPA. Adolescent pregnancy. [Internet]. 2022 [cited 2022 Sep 6]. Available from: https://esaro.unfpa.org/en/topics/adolescent-pregnancy

11. Yakubu I, Salisu WJ. Determinants of adolescent pregnancy in sub-Saharan Africa: A systematic review. Reprod Health. 2018;15(1).

12. Gunawardena N, Fantaye AW, Yaya S. Predictors of pregnancy among young people in sub-Saharan Africa: A systematic review and narrative synthesis. BMJ Glob Heal. 2019;4(3):1–8.

13. Asare BYA, Baafi D, Dwumfour-Asare B, Adam AR. Factors associated with adolescent pregnancy in the Sunyani Municipality of Ghana. Int J Africa Nurs Sci. 2019;10(February):87–91.

14. Bedzo JY, Manortey S. Factors Influencing Teenage Pregnancy in the Lower Manya Krobo Municipality in the Eastern Region of Ghana: A Cross-Sectional Study. OALib. 2019;06(12):1–17.

15. The DHS Program. Publications Catalog [Internet]. 2022 [cited 2022 Feb 4]. Available from: https://dhsprogram.com/Publications/Publication-Search.cfm?ctry_id=14&country=Ghana

For peer review only - http://bmjopen.bmj.com/site/about/guidelines.xhtml

16. Higgins JPT, Thompson SG. Quantifying heterogeneity in a meta-analysis. Stat Med. 2002;21(11):1539–58.

17. Gayatri M, Gayatri M, Permata J, Kusuma HP, Timur J. Socioeconomic determinants of adolescent pregnancy in Indonesia : A population-based survey Key Messages : Ann Trop Med Public Heal. 2021;24(01).

18. Byonanebye J, Brazauskas R, Tumwesigye N, Young S, May T, Cassidy L. Geographic variation and risk factors for teenage pregnancy in Uganda. Afr Health Sci. 2020;20(4):1898–907.

19. Uwizeye D, Muhayiteto R, Kantarama E, Wiehler S, Murangwa Y. Prevalence of teenage pregnancy and the associated contextual correlates in Rwanda. Heliyon [Internet]. 2020;6(10):e05037. Available from: https://doi.org/10.1016/j.heliyon.2020.e05037

20. Jonas K, Crutzen R, Van Den Borne B, Sewpaul R, Reddy P. Teenage pregnancy rates and associations with other health risk behaviours: A three-wave cross-sectional study among South African school-going adolescents. Reprod Health [Internet]. 2016;13(1). Available from: http://dx.doi.org/10.1186/s12978-016-0170-8

21. Morhe ESK, Tagbor HK, Ankobea FK, Danso KA. Reproductive experiences of teenagers in the Ejisu-Juabeng district of Ghana. Int J Gynecol Obstet [Internet]. 2012;118(2):137–40. Available from: http://dx.doi.org/10.1016/j.ijgo.2012.03.035

22. Juma M, Alaii J, Bartholomew LK, Askew I, Van Den Born B. Understanding orphan and non-orphan adolescents' sexual risks in the context of poverty: A qualitative study in Nyanza Province, Kenya. BMC Int Health Hum Rights. 2013;13(32).

23. Alemayehu T, Haider J, Habte D. Determinants of adolescent fertility in Ethiopia [Internet]. Vol. 24, The Ethiopian Journal of Health Development. 2010. p. 30–8. Available from: http://search.ebscohost.com/login.aspx?direct=true&AuthType=cookie,ip,shib&db=awn&AN=ejhd-62942&site=ehost-live

24. UNICEF. Early marriage: child spouses. [Internet]. Innocenti Digest. Florence, Italy Piazza; 2001. Available from: https://www.unicef-irc.org/publications/pdf/digest7e.pdf

25. McCleary-Sills J, Douglas Z, Rwehumbiza A, Hamisi A, Mabala R. Gendered norms, sexual exploitation and adolescent pregnancy in rural Tanzania. Reprod Health Matters [Internet]. 2013;21(41):97–105. Available from: http://dx.doi.org/10.1016/S0968-8080(13)41682-8

26. Krugu JK, Mevissen FEF, Prinsen A, Ruiter RAC. Who's that girl? A qualitative analysis of adolescent girls' views on factors associated with teenage pregnancies in Bolgatanga, Ghana. Reprod Health [Internet]. 2016;13(1):1–12. Available from: http://dx.doi.org/10.1186/s12978-016-0161-9

27. Zakayo MO, Lwelamira J. Sexual Behaviours among Adolescents in Community Secondary Schools in Rural Areas of Central Tanzania: A Case of Bahi District in Dodoma Region. Curr Res J Soc Sci. 2011;3(4):374–80.

28. Nabugoomu J, Seruwagi GK, Hanning R. What can be done to reduce the prevalence of teen pregnancy in rural Eastern Uganda?: Multi-stakeholder perceptions. Reprod Health. 2020;17(134):1–12.

For peer review only - http://bmjopen.bmj.com/site/about/guidelines.xhtml

For peer review only

For peer review only - http://bmjopen.bmj.com/site/about/guidelines.xhtml

For peer review only

For peer review only - http://bmjopen.bmj.com/site/about/guidelines.xhtml

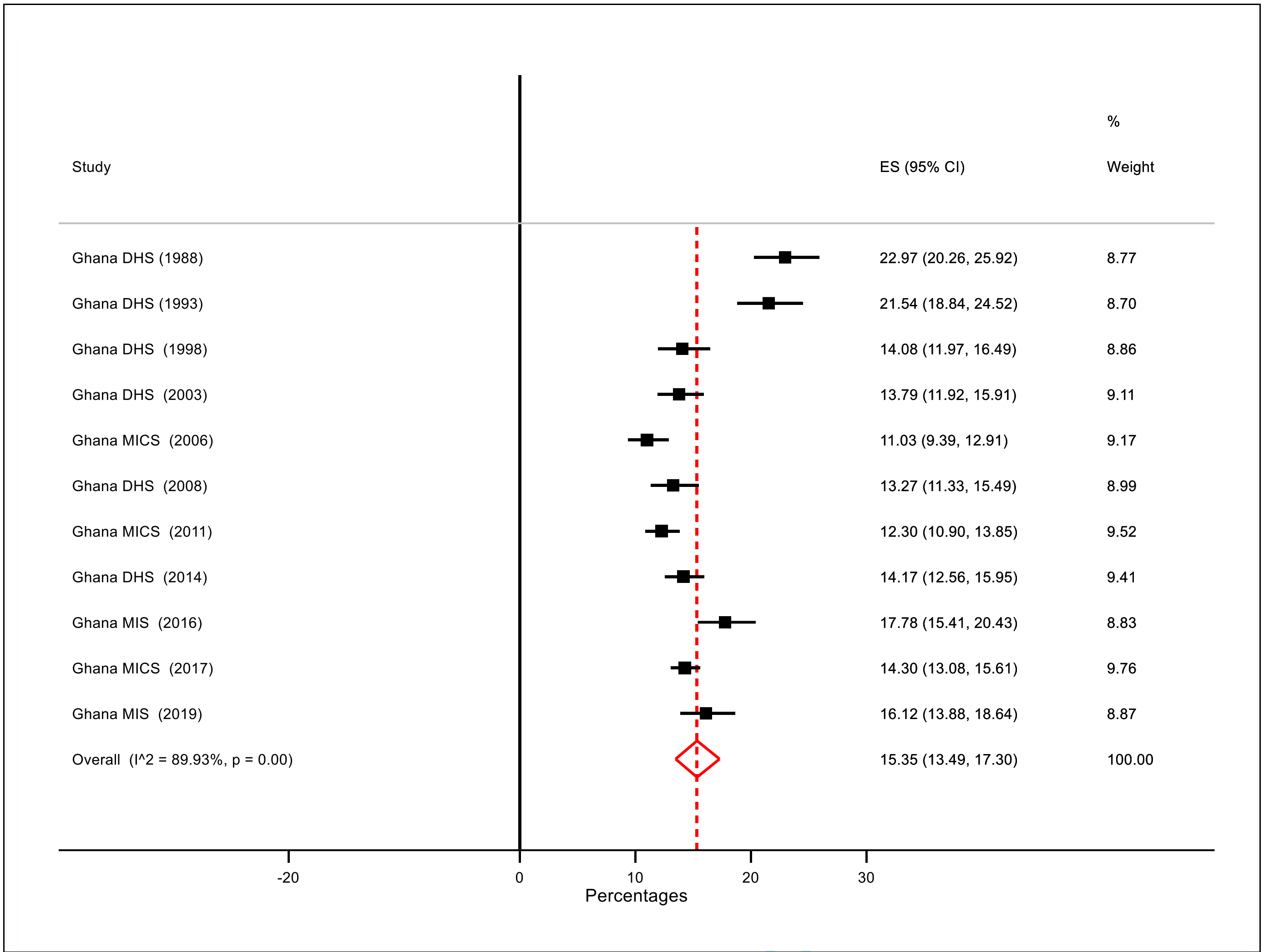

Figure 1: National prevalence of adolescent pregnancy , 1988 - 2019

For peer review only - http://bmjopen.bmj.com/site/about/guidelines.xhtml

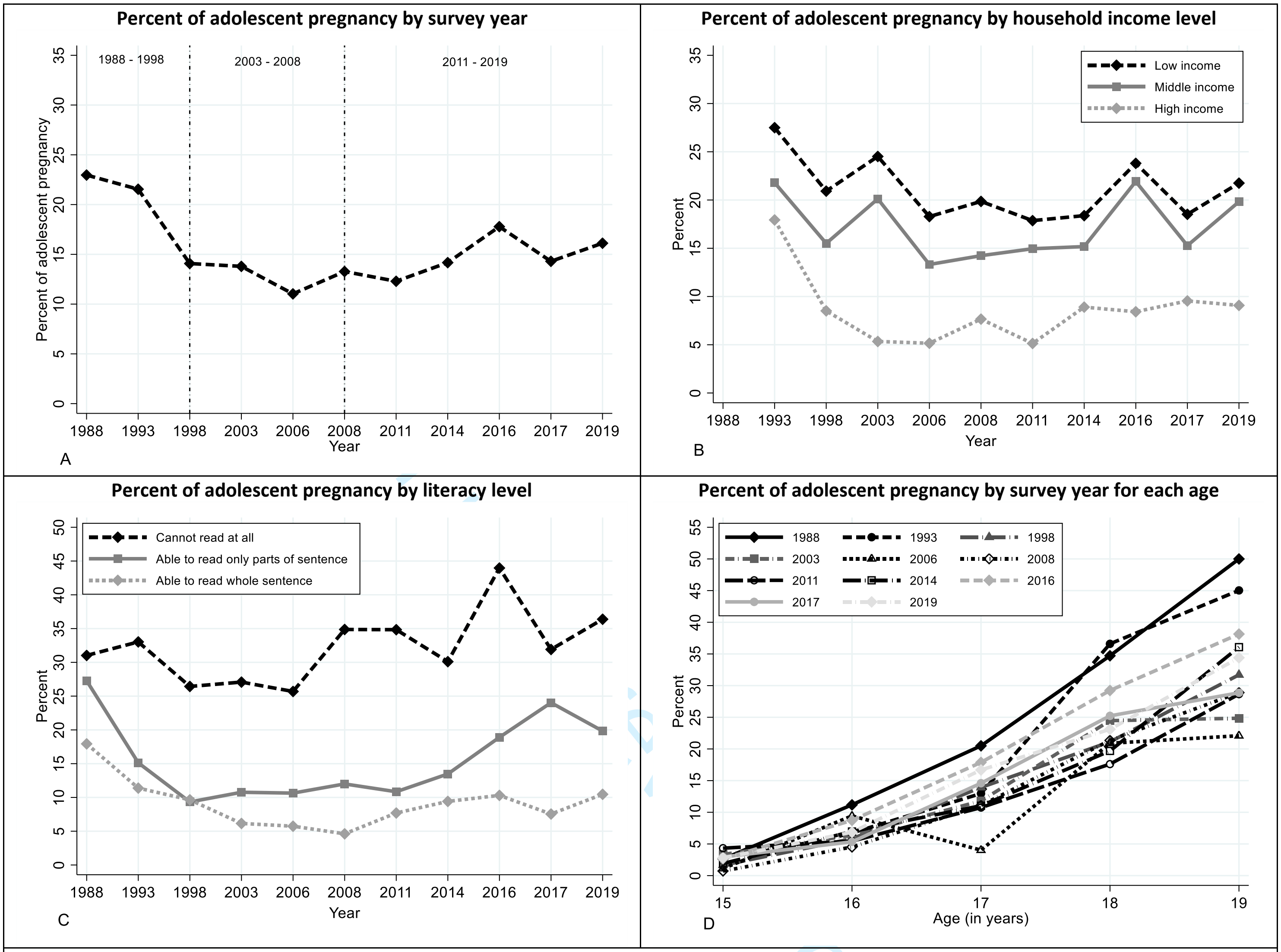

Figure 2: Time trends of adolescent pregnancy by (A) Survey year (B) household income (C) literacy level (D) age

Supplementary Table 1. Conceptual model of factors associated with adolescent pregnancy

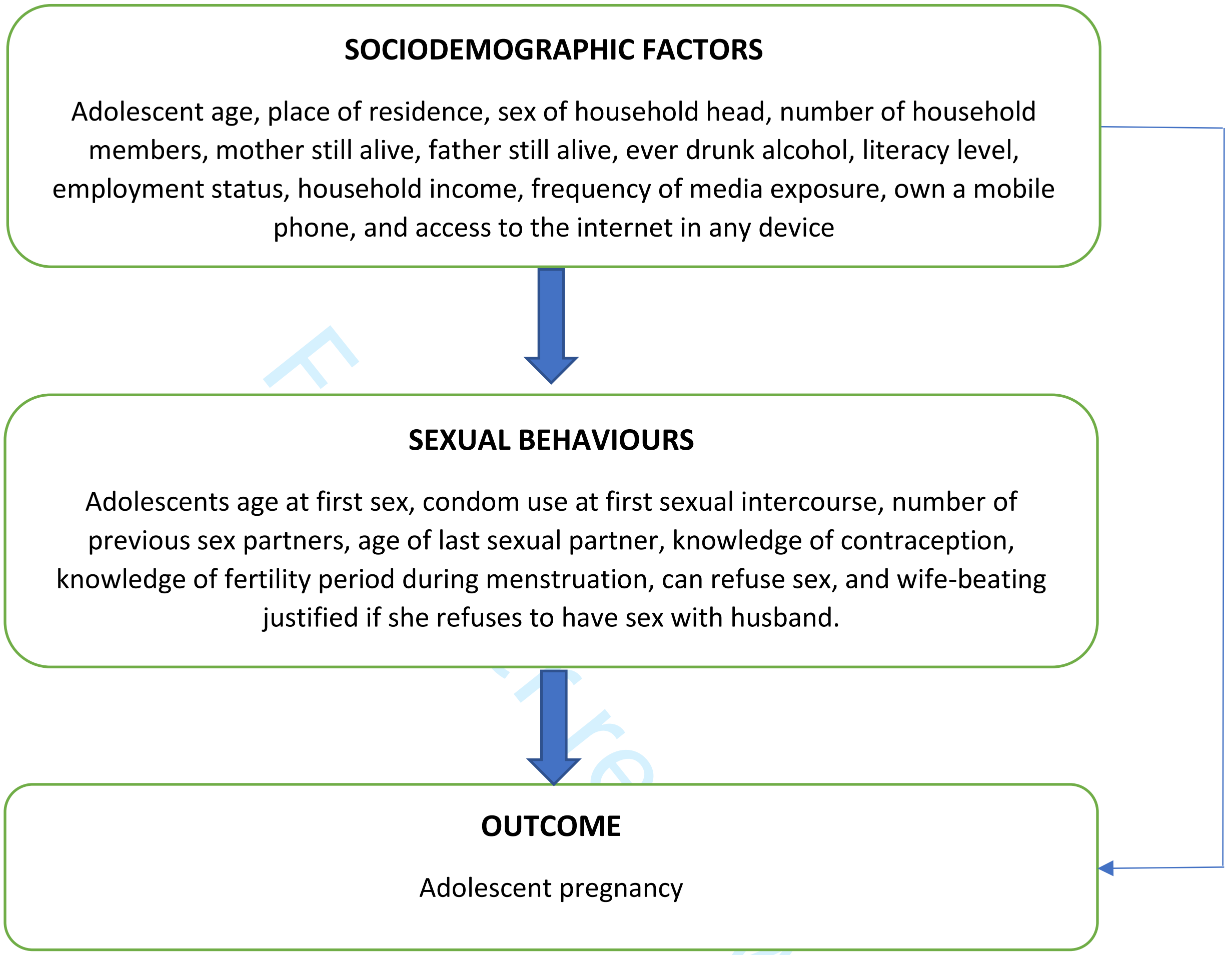

Supplementary Table 2 Unadjusted analysis of the sociodemographic factors associated with adolescent pregnancy, 1988-2019.

Survey years	1988 - 1998	2003 - 2008	2011 - 2019	Overall sample (1988-2019)
Population analysed (n)	2541	3350	8665	14556
Number currently pregnant	96	117	282	495
	**Unadjusted odds ratio (95% CI)**	**Unadjusted odds ratio (95% CI)**	**Unadjusted odds ratio (95% CI)**	**Unadjusted odds ratio (95% CI)**
**Place of residence**	P = 0.08	P < 0.001	P < 0.001	P < 0.001
Urban	0.64 (0.39 - 1.06)	0.40 (0.24 - 0.66)	0.53 (0.38 - 0.74)	0.52 (0.41 - 0.66)
Rural	1.00	1.00	1.00	1.00
**Sex of household head**	P = 0.68	P = 0.09	P = 0.37	P = 0.89
Male	1.00	1.00	1.00	1.00
Female	0.87 (0.46 - 1.67)	1.45 (0.94 - 2.25)	0.86 (0.61 - 1.20)	0.98 (0.76 - 1.27)
**Number of household members**	P < 0.001	P < 0.001	P = 0.01	P < 0.001
1 - 4 members	1.00	1.00	1.00	1.00
More than 4 members	0.43 (0.28 - 0.67)	0.48 (0.32 - 0.71)	0.65 (0.47 - 0.90)	0.56 (0.45 - 0.69)
**Mother still alive**	P = 0.07		P = 0.95	P = 0.53
Yes	0.30 (0.08 - 1.10)	-	1.04 (0.25 - 4.37)	0.76 (0.33 - 1.77)
No	1.00	-	1.00	1.00
**Father still alive**	P = 0.86	P = 0.74	P = 0.15	P = 0.16
Yes	1.00	1.00	1.00	1.00
No	1.10 (0.37 - 3.24)	1.23 (0.36 - 4.19)	1.69 (0.83 - 3.41)	1.47 (0.87 - 2.48)
**Literacy level**	P < 0.001	P < 0.001	P < 0.001	P < 0.001
Cannot read at all	1.00	1.00	1.00	1.00
Able to read only partially sentence	0.44 (0.18 - 1.07)	0.36 (0.19 - 0.68)	0.52 (0.30 - 0.89)	0.48 (0.33 - 0.70)
Able to read whole sentence	0.31 (0.19 - 0.51)	0.18 (0.11 - 0.29)	0.22 (0.15 - 0.34)	0.24 (0.18 - 0.31)
**Employment**	P < 0.001	P < 0.001	P = 0.02	P < 0.001
Yes	1.00	1.00	1.00	1.00
No	0.40 (0.25 - 0.62)	0.35 (0.21 - 0.58)	0.46 (0.24 - 0.88)	0.39 (0.29 - 0.52)
**Household income**	P = 0.13	P < 0.001	P < 0.001	P < 0.001
Low income	1.00	1.00	1.00	1.00
Middle income	0.56 (0.27 - 1.15)	0.65 (0.41 - 1.03)	0.74 (0.50 - 1.09)	0.70 (0.53 - 0.93)
High income	0.57 (0.31 - 1.05)	0.22 (0.13 - 0.38)	0.42 (0.27 - 0.63)	0.37 (0.27 - 0.50)
**Frequency of media exposure**		P = 0.02	P = 0.50	P = 0.73
Not at all	-	2.10 (1.10 - 4.01)	0.99 (0.65 - 1.52)	1.13 (0.79 - 1.61)

For peer review only

Less than once a week	-	2.33 (0.94 - 5.74)	0.72 (0.41 - 1.26)	0.93 (0.58 - 1.49)
At least once a week	-	1.00	1.00	1.00
**Education level**	P < 0.001	P < 0.001	P < 0.001	P < 0.001
No education	1.00	1.00	1.00	1.00
Primary school	0.84 (0.38 - 1.86)	0.91 (0.52 - 2.59)	1.21 (0.67 - 2.21)	1.03 (0.73 - 1.46)
Junior high school	0.27 (0.14 - 0.52)	0.39 (0.23 - 0.67)	0.66 (0.38 - 1.14)	0.50 (0.37 - 0.69)
Senior high or post-secondary school	0.07 (0.01 - 0.59)	0.01 (0.00 - 0.09)	0.15 (0.07 - 0.32)	0.10 (0.06 - 0.18)

For peer review only

Supplementary Table 3 Unadjusted analysis of the sexual behaviours associated with adolescent pregnancy, 1988-2019.

Survey years	1988 - 1998	2003 - 2008	2011 - 2019	Overall sample (1988-2019)
Population analysed (n)	2541	3350	8665	14556
Number currently pregnant	96	117	282	495
	**Unadjusted odds ratio (95% CI)**	**Unadjusted odds ratio (95% CI)**	**Unadjusted odds ratio (95% CI)**	**Unadjusted odds ratio (95% CI)**
**Age at first sex**	P = 0.47	P = 0.37	P = 0.32	P = 0.43
Before age 16	0.84 (0.51 - 1.36)	1.27 (0.75 - 2.14)	1.22 (0.83 - 1.80)	1.11 (0.85 - 1.45)
16 - 19 years	1.00	1.00	1.00	1.00
**Number of previous sex partners**		P = 0.06	P = 0.72	P = 0.08
1	-	0.28 (0.10 - 0.83)	0.70 (0.28 - 1.75)	0.46 (0.23 - 0.93)
2	-	0.55 (0.20 - 1.53)	0.85 (0.31 - 2.31)	0.69 (0.34 - 1.41)
Three or more	-	1.00	1.00	1.00
**Age of last sexual partner**		P = 0.02	P = 0.26	P = 0.53
Below 20 years	-	1.00	1.00	1.00
More than 20 years	-	0.11 (0.02 - 0.65)	1.56 (0.72 - 3.37)	1.24 (0.63 - 2.45)
**Knowledge of contraception**	P = 0.09	P = 0.34		P = 0.06
Knows no method	0.55 (0.27 - 1.10)	0.57 (0.18 - 1.79)	-	0.57 (0.32 - 1.03)
Knows modern or traditional method	1.00	1.00	-	1.00
**knowledge of fertility period during menstruation**	P = 0.88	P = 0.23		P = 0.78
Just before or during menstrual period	1.35 (0.36 - 5.13)	1.85 (0.74 - 4.61)	-	0.81 (0.44 - 1.52)
Right after menstrual period or at any time	0.97 (0.44 - 2.16)	1.75 (0.90 - 3.39)	-	1.00 (0.67 - 1.49)
Halfway between two menstrual periods	1.00	1.00	-	1.00
**Can refuse sex**	-	P = 0.72	P = 0.27	P = 0.35
Yes	-	1.00	1.00	1.00
No	-	0.80 (0.24 - 2.65)	0.46 (0.11 - 1.87)	0.65 (0.26 - 1.62)
**Wife beating justified if she refuses to have sex with him**		P = 0.90	P = 0.46	P = 0.49
Yes	-	1.03 (0.61 - 1.75)	1.20 (0.74 - 1.94)	1.14 (0.79 - 1.64)
No	-	1.00	1.00	1.00

For peer review only

STROBE Statement—Checklist of items that should be included in reports of ***cross-sectional studies***

	Item No	Recommendation	Page No
**Title and abstract**	1	(*a*) Indicate the study's design with a commonly used term in the title or the abstract	1
		(*b*) Provide in the abstract an informative and balanced summary of what was done and what was found	2
**Introduction**			
Background/rationale	2	Explain the scientific background and rationale for the investigation being reported	3
Objectives	3	State specific objectives, including any prespecified hypotheses	3
**Methods**			
Study design	4	Present key elements of study design early in the paper	3
Setting	5	Describe the setting, locations, and relevant dates, including periods of recruitment, exposure, follow-up, and data collection	4
Participants	6	(*a*) Give the eligibility criteria, and the sources and methods of selection of participants	4
Variables	7	Clearly define all outcomes, exposures, predictors, potential confounders, and effect modifiers. Give diagnostic criteria, if applicable	4
Data sources/ measurement	8*	For each variable of interest, give sources of data and details of methods of assessment (measurement). Describe comparability of assessment methods if there is more than one group	*4*
Bias	9	Describe any efforts to address potential sources of bias	4
Study size	10	Explain how the study size was arrived at	4
Quantitative variables	11	Explain how quantitative variables were handled in the analyses. If applicable, describe which groupings were chosen and why	4
Statistical methods	12	(*a*) Describe all statistical methods, including those used to control for confounding	4
		(*b*) Describe any methods used to examine subgroups and interactions	4
		(*c*) Explain how missing data were addressed	4
		(*d*) If applicable, describe analytical methods taking account of sampling strategy	4
		(*e*) Describe any sensitivity analyses	NA
**Results**			
Participants	13*	(a) Report numbers of individuals at each stage of study—eg numbers potentially eligible, examined for eligibility, confirmed eligible, included in the study, completing follow-up, and analysed	5
		(b) Give reasons for non-participation at each stage	
		(c) Consider use of a flow diagram	
Descriptive data	14*	(a) Give characteristics of study participants (eg demographic, clinical, social) and information on exposures and potential confounders	5
		(b) Indicate number of participants with missing data for each variable of interest	
Outcome data	15*	Report numbers of outcome events or summary measures	5
Main results	16	(*a*) Give unadjusted estimates and, if applicable, confounder-adjusted estimates and their precision (eg, 95% confidence interval). Make clear which confounders were adjusted for and why they were included	5-6

For peer review only - http://bmjopen.bmj.com/site/about/guidelines.xhtml

		(*b*) Report category boundaries when continuous variables were categorized	NA
		(*c*) If relevant, consider translating estimates of relative risk into absolute risk for a meaningful time period	
Other analyses	17	Report other analyses done—eg analyses of subgroups and interactions, and sensitivity analyses	NA
**Discussion**			
Key results	18	Summarise key results with reference to study objectives	6
Limitations	19	Discuss limitations of the study, taking into account sources of potential bias or imprecision. Discuss both direction and magnitude of any potential bias	8-9
Interpretation	20	Give a cautious overall interpretation of results considering objectives, limitations, multiplicity of analyses, results from similar studies, and other relevant evidence	7
Generalisability	21	Discuss the generalisability (external validity) of the study results	8-9
**Other information**			
Funding	22	Give the source of funding and the role of the funders for the present study and, if applicable, for the original study on which the present article is based	10

*Give information separately for exposed and unexposed groups.

Note: An Explanation and Elaboration article discusses each checklist item and gives methodological background and published examples of transparent reporting. The STROBE checklist is best used in conjunction with this article (freely available on the Web sites of PLoS Medicine at http://www.plosmedicine.org/, Annals of Internal Medicine at http://www.annals.org/, and Epidemiology at http://www.epidem.com/). Information on the STROBE Initiative is available at www.strobe-statement.org.

For peer review only

For peer review only - http://bmjopen.bmj.com/site/about/guidelines.xhtml

BMJ Open

National and subnational prevalence of adolescent pregnancy and changes in the associated sexual behaviours and sociodemographic determinants across three decades in Ghana, 1988 – 2019: analysis of national surveys

Journal:	*BMJ Open*
Manuscript ID	bmjopen-2022-068117.R1
Article Type:	Original research
Date Submitted by the Author:	23-Feb-2023
Complete List of Authors:	Mohammed, Shamsudeen; London School of Hygiene & Tropical Medicine, Department of Non-communicable Disease Epidemiology
**Primary Subject Heading**:	Public health
Secondary Subject Heading:	Epidemiology, Global health, Sexual health, Obstetrics and gynaecology
Keywords:	Epidemiology < INFECTIOUS DISEASES, Public health < INFECTIOUS DISEASES, REPRODUCTIVE MEDICINE

SCHOLARONE™
Manuscripts

For peer review only - http://bmjopen.bmj.com/site/about/guidelines.xhtml

I, the Submitting Author has the right to grant and does grant on behalf of all authors of the Work (as defined in the below author licence), an exclusive licence and/or a non-exclusive licence for contributions from authors who are: i) UK Crown employees; ii) where BMJ has agreed a CC-BY licence shall apply, and/or iii) in accordance with the terms applicable for US Federal Government officers or employees acting as part of their official duties; on a worldwide, perpetual, irrevocable, royalty-free basis to BMJ Publishing Group Ltd ("BMJ") its licensees and where the relevant Journal is co-owned by BMJ to the co-owners of the Journal, to publish the Work in this journal and any other BMJ products and to exploit all rights, as set out in our licence.

The Submitting Author accepts and understands that any supply made under these terms is made by BMJ to the Submitting Author unless you are acting as an employee on behalf of your employer or a postgraduate student of an affiliated institution which is paying any applicable article publishing charge ("APC") for Open Access articles. Where the Submitting Author wishes to make the Work available on an Open Access basis (and intends to pay the relevant APC), the terms of reuse of such Open Access shall be governed by a Creative Commons licence – details of these licences and which Creative Commons licence will apply to this Work are set out in our licence referred to above.

Other than as permitted in any relevant BMJ Author's Self Archiving Policies, I confirm this Work has not been accepted for publication elsewhere, is not being considered for publication elsewhere and does not duplicate material already published. I confirm all authors consent to publication of this Work and authorise the granting of this licence.

For peer review only

National and subnational prevalence of adolescent pregnancy and changes in the associated sexual behaviours and sociodemographic determinants across three decades in Ghana, 1988 – 2019: analysis of national surveys

Shamsudeen Mohammed [1]

[1]Department of Non-communicable Disease Epidemiology, Faculty of Epidemiology and Population Health, London School of Hygiene & Tropical Medicine, London, UK,

Correspondence: Shamsudeen Mohammed, Department of Non-communicable Disease Epidemiology, London School of Hygiene and Tropical Medicine, Keppel Street, London WC1E 7HT Shamsudeen.Mohammed1@lshtm.ac.uk

For peer review only

Abstract

Objective

Understanding the determinants of adolescent pregnancy and how they have changed over time is essential to measuring progress and identifying approaches to improving adolescent reproductive health. This study examined changes over time in the prevalence and determinants of adolescent pregnancy in Ghana.

Methods

Eleven nationally representative surveys from the Ghana Demographic and Health Survey (1988, 1993, 1998, 2003, 2008, 2014), Multiple Indicator Cluster Survey (2006, 2011, 20117-18), and Malaria Indicator Survey (2016 and 2019) provided data on 14556 adolescents girls aged 15-19 for this analysis. A random-effect meta-analysis, time trends, and multivariable logistic regression models were used to track the prevalence and determinants of adolescent pregnancy.

Results

The prevalence of adolescent pregnancy in Ghana was 15.4% (95%CI=13.49%–17.30%). Rural areas (19.5%) had a higher prevalence of adolescent pregnancy than urban areas (10.6%). In the overall sample, middle adolescents (15-17 years) (aOR=0.30, 95%CI=0.23-0.39), adolescents in urban areas (aOR=0.56, 95%CI=0.43 - 0.74), large households (aOR=0.62, 95%CI=0.49-0.78), not working (aOR=0.62, 95%CI=0.43 - 0.90), and those unaware of contraceptive methods (aOR=0.49, 95%CI=0.27-0.90) were less likely to become pregnant. Adolescents from middle-income (aOR=0.91, 95%CI=0.67-1.24) or high-income (aOR=0.59, 95%CI=0.36-0.94) households, those who were semi-literate (aOR=0.56, 95%CI=0.39-0.82) or literate (aOR=0.28, 95%CI=0.21-0.37) and those with fewer previous sex partners were less likely to become pregnant. Not all determinants in the overall sample were consistently associated with adolescent pregnancy in the last three decades. Between 1988 and 1998, determinants of adolescent pregnancy were age, literacy, employment, household size, and whether the mother was alive. Between 2003 and 2008, age, literacy, household size, income, age of last sexual partner, number of previous partners, and contraception knowledge determined adolescent pregnancy. From 2011 to 2019, age, residence, literacy, and menstrual cycle knowledge were determinants of adolescent pregnancy.

Conclusion

Interventions and policies to prevent adolescent pregnancy should prioritise those from disadvantaged backgrounds.

Strengths and limitations of this study

The study included eleven nationally representative surveys across three decades.

Because the sample size was large, the study had adequate power to detect and estimate more reliable results.

Adolescents from varied sociodemographic backgrounds were included in the study sample.

For peer review only - http://bmjopen.bmj.com/site/about/guidelines.xhtml

The secondary data sources lacked information on cultural and traditional practices that may influence adolescent pregnancy.

Background

Globally, one in five adolescent girls gives birth before age 18(1). In low- and middle-income countries (LMICs), about 21 million teenagers become pregnant each year(2), and if current trends continue, that number is expected to rise to 86 million by 2030 (1). The prevalence is highest in South Asia and sub-Saharan Africa(1). In 2018, a systematic review and meta-analysis of 52 studies revealed that the prevalence of adolescent pregnancy in sub-Saharan Africa is 19.3%, with substantial regional variation ranging from 16% in Central Africa to 22% in East Africa(3). The consequences of adolescent pregnancy can be devastating. For instance, teenagers who become pregnant face many negative social consequences, including isolation, stigma, rejection from peers and family, forced marriage, and even the risk of dropping out of school(4). Studies have shown that adolescents who become pregnant face an increased risk of unsafe abortion, eclampsia, obstruction during childbirth, obstetric fistula, preterm birth, and maternal death or disability(2,5–10).

Many epidemiological studies have shown a considerable disparity in the drivers of adolescent pregnancy, where disadvantaged adolescents are more at risk of pregnancy than those better off. For example, child marriage, non-school attendance, poverty, living in rural residence, household living arrangement, no maternal education, no paternal education, history of maternal teenage pregnancy, cost of contraceptives, misconceptions about contraceptives, and lack of parental counselling and guidance on sexual and reproductive health have been identified as the main sociodemographic and economic factors that drive adolescent pregnancy in Africa(3,11,12). Furthermore, sexual risk factors, including unwanted sexual advances from adult males, coercive sexual relations, unequal gender power relations, lack of comprehensive sexuality education, and early sexual debut, also increase adolescents susceptibility to pregnancy(3,10–12).

These findings from prior research demonstrate that adolescent pregnancy results from a complex interaction of social, economic, cultural, and personal factors. Understanding these different determinants can help identify the groups of adolescents most at risk of becoming pregnant. It can also inform interventions and policies to prevent adolescent pregnancy and improve the sexual and reproductive health of adolescent girls. Furthermore, tracking changes in the determinants over time can provide valuable information for assessing the efficacy of existing interventions and programs to reduce adolescent pregnancies.

In Ghana, not many studies have explored the predictors of adolescent pregnancy, despite the need for country-specific estimates and determinants to inform national policies and programmes on adolescent pregnancy and interventions targeted at improving the lives of adolescents. Moreover, the few studies conducted over the last few years had small samples and reported contradictory findings(13,14). The present study used data from eleven nationally representative surveys conducted between 1988 and 2019 to estimate the national and regional prevalence of adolescent pregnancy and track changes in the sociodemographic and sexual risk behaviours associated with adolescent pregnancy across three decades.

For peer review only - http://bmjopen.bmj.com/site/about/guidelines.xhtml

Methods

Eleven nationally representative cross-sectional surveys from the Ghana Demographic and Health Survey (1988, 1993, 1998, 2003, 2008, 2014), Multiple Indicator Cluster Survey (2006, 2011, 20117-18), and Malaria Indicator Survey (2016 and 2019) provided the data for this analysis. A detailed description of the methods used in the surveys, including information about survey design, sampling, and data collection, is published elsewhere(15). Briefly, the surveys used a multistage stratified cluster sampling method to select households from rural and urban clusters in Ghana's former ten administrative regions (now 16 regions). The participant questionnaires administered in these surveys are comparable, allowing data pooling across surveys and time. For all the surveys, trained field officers under the supervision of the Ghana Statistical Services administered the questionnaires face-to-face to women and men in selected households. The response rate in the surveys was higher than 90%. Data on only women aged 15 to 19 years were extracted from data files of the eleven surveys for the present analysis.

Variables

The outcome of interest was adolescent pregnancy. In this study, it was defined as women aged 15 to 19 who had previously given birth or were pregnant at the time of the survey. Across the surveys, the same questions were asked to elicit responses to measure the outcome. Women were asked, *"are you pregnant now?"* to determine those pregnant at the time of the survey and *"have you ever given birth?"* to ascertain those who had ever given birth. Response options for both questions were *"Yes"* or *"No"*. In order to reduce reverse causation and recall bias, the analysis of the factors associated with adolescent pregnancy was restricted to adolescents who were pregnant at the time of the survey. Adolescents sociodemographic and sexual risk behaviours were explored as potential explanatory factors of adolescent pregnancy. The sociodemographic factors included adolescent age, place of residence, sex of household head, number of household members *("≤4 members" and ">4 members"* (16)), mother still alive, father still alive, literacy level (*illiterate* = "cannot read at all", *semi-literate* = "able to read only partially sentence", and *literate* = "able to read whole sentence"), employment status, household income, and frequency of media exposure (newspaper + radio + TV). The sexual behaviours that were considered in the analysis are adolescents age at first sex *("≤ 14 years" vs "≥ 15 years"* (17)), number of previous sex partners, age of last sexual partner, knowledge of contraception, and knowledge of fertility period during menstruation. The explanatory variables were not available for all surveys.

Data management and statistical analysis

Data from each survey year were standardised and pooled into a single dataset before being grouped into three periods, 1988-1998, 2003-2008, and 2011-2019. The prevalence of adolescent pregnancy was estimated as a percentage of all adolescents aged 15 to 19 years for each survey year, the three eras, and the overall sample. Time trends of adolescent pregnancy and age patterns were plotted for survey years and selected sociodemographic factors. A random effect meta-analysis was used to pool the prevalence across the survey years to estimate the prevalence of adolescent pregnancy in Ghana. The pooled prevalence and corresponding 95% confidence intervals were presented using forest plots. Heterogeneity among the surveys was assessed using Higgins and Thompson's I^2 statistic(18). Subgroup analyses were

For peer review only - http://bmjopen.bmj.com/site/about/guidelines.xhtml

performed to assess variations in the prevalence across the administrative regions of Ghana and rural-urban residence. To scale prevalence estimates to represent all adolescents in Ghana, denormalised survey weights were applied to correct for unequal sampling in certain areas and subgroups.

Bivariate logistic regression was used to investigate the unadjusted association between the explanatory variables and the binary outcome, adolescent pregnancy. Multivariable logistic regression was then used to determine the factors associated with adolescent pregnancy across the three decades (1988-1998, 2003-2008, and 2011-2019) and the overall sample (1988-2019) in an adjusted analysis. Potential determinants were categorised into distal (sociodemographic factors) and proximal (sexual behaviours) factors, and a conceptual framework was used to guide the analysis (Supplementary Figure 1). Based on the framework, distal factors were considered potential confounders of the association between the proximal factors and the outcome in the multivariable model. The logistic regression models accounted for stratification in the survey design, the year surveys were conducted, and calculated robust standard errors to account for clustering in the design of the surveys. To minimise the influence of reverse causation and recall bias, the logistic regression analysis was restricted to adolescents who were pregnant at the time of the survey. Because this study is exploratory rather than confirmatory, P<0.10 was used to determine statistically significant associations. A test for linear trend was performed for "literacy level", "household income", and "number of previous sex partners" using the Stata postestimation command, *contrast*. Estimates for explanatory variables not available in all the surveys were produced in a separate multivariable model. Stata version 17 was used for all data management and analysis.

Ethics approval

The Ghana Statistical Services and DHS program obtained ethical approval, participant consent, and permission for each primary survey. MEASURE DHS granted permission and access for the secondary use of the survey data for the present analysis. Because the data is available in the public domain with no personal identifiers, ethical approval was not sought for the secondary analysis.

Patient and public involvement

Patients or the public were not involved in the design, conduct, reporting, or dissemination plans of our research.

Results

Characteristics of the study sample

A nationally representative sample of 14556 adolescents aged 15 to 19 years from surveys conducted between 1988 and 2019 was included in the analysis. Table 1 presents the characteristics of the total study sample. Most adolescents lived in rural areas, in households with high income, headed by men, and with more than five household members. The majority of the sample were middle adolescents aged 15 to 17 years. Only a small percentage of the adolescents were orphans or worked at some point in the 12 months before the surveys. Nearly a third (28%) of the adolescents were illiterate, and 63.1% were exposed to mainstream media at least once a week (radio, television, or newspaper). Three-fourths (75.9%) of the adolescents initiated sex after age 14, and 66.7% had had only one sexual relationship.

For peer review only - http://bmjopen.bmj.com/site/about/guidelines.xhtml

Most adolescents were aware of modern or traditional contraceptive methods, and for the majority of those who had had a sexual partner, their last sexual partner was an adult over age 20.

Table 1 Characteristics of the study population, 1988-2019 (n=14556)

	Unweighted number	Weighted percent
**Age**		
15 - 17	8986	62.4
18 - 19	5570	37.6
**Place of residence**		
Urban	6254	47.1
Rural	8302	52.9
**Sex of household head**		
Male	8718	59.9
Female	4989	40.1
**Number of household members**		
≤4 members	4209	30.8
>4 members	10347	69.2
**Mother still alive**		
Yes	5454	94.2
No	309	5.8
**Father still alive**		
Yes	4965	86.1
No	790	13.9
**Literacy level**		
Illiterate	3540	27.9
Semi-literate	1932	14.1
Literate	6238	58.1
**Employment**		
Yes	1985	30.3
No	4442	69.7
**Household income**		
Low income	6268	36.9
Middle income	2561	21.2
High income	4878	41.9
**Frequency of media exposure**		
Not at all	2483	22.1
Less than once a week	1282	14.8
At least once a week	5134	63.1
**Age at first sex**		
≤14 years	1107	24.1

For peer review only - http://bmjopen.bmj.com/site/about/guidelines.xhtml

≥15 years	3648	75.9
**Number of previous sex partners**		
1	764	66.7
2	243	23.8
Three or more	106	9.4
**Age of last sexual partner**		
Below 20 years	381	28.4
More than 20 years	993	71.6
**Knowledge of contraception**		
Knows no method	666	14.1
Knows modern or traditional method	4892	85.9
**knowledge of fertility period during menstruation**		
Just before or during menstrual period	546	14.6
Right after menstrual period or at any time	1964	52.1
Halfway between two menstrual periods	1067	33.3

Prevalence and time trends of adolescent pregnancy, 1988 - 2019

Based on a meta-analysis of the eleven national surveys, the prevalence of adolescent pregnancy was 15.4% (95%CI=13.49% – 17.30%) with significant variation across the surveys (I^2 = 89.9%, p < 0.001; Figure 1). There was a substantial regional disparity in the prevalence, ranging from 8.2% (95%CI=6.29%-10.39%) in the Greater Accra region to 19.3% (95% CI=15.75%-23.19%) in the Central region (Table 2; see Supplementary Figure 2 for details). Table 2 shows that the prevalence of adolescent pregnancy in rural areas (19.5%) was nearly twice that of urban areas (10.6%) (Table 2; see Supplementary Figure 3 for details).

The prevalence of adolescent pregnancy declined between 1988 and 1998, then remained relatively consistent from 2003 to 2008 before gradually increasing in the last decade (figure 2A). Adolescents from high-income households (Figures 2B) and those who were literate (Figures 2C) had a lower prevalence of adolescent pregnancy throughout the eleven surveys, with the steepest decline seen between 1993 and 2006. The surveys showed an upward trend of adolescent pregnancy with increasing age (figure 2D). There was no consistent pattern in the prevalence of adolescent pregnancy across ethnic groups.

Table 2 Subnational prevalence of adolescent pregnancy based on a meta-analysis of nationally representative surveys from 1988 to 2019 (see supplementary Figure 2 and supplementary Figure 3 for detailed forest plots for each region and urban-rural residence, respectively).

	Prevalence of adolescent pregnancy	95% confidence interval	I^2
**Region**			
Western region	14.42%	11.27% - 17.87%	66.5%
Central region	19.34%	15.75% - 23.19%	65.0%
Greater Accra region	8.23%	6.29% - 10.39%	62.6%
Volta region	16.18%	13.50% - 19.05%	36.8%
Eastern region	16.20%	13.93% - 18.61%	31.6%
Ashanti region	14.14%	11.94% - 16.48%	60.7%
Brong-Ahafo region	16.73%	12.66% - 21.22%	75.8%

For peer review only - http://bmjopen.bmj.com/site/about/guidelines.xhtml

Northern region	16.41%	11.62% - 21.82%	79.7%
Upper West region	11.48%	8.48% - 14.82%	0.0%
Upper East region	11.40%	8.73% - 14.36%	0.0%
**Residence**			
Urban	10.59%	8.69% - 12.66%	85.6%
Rural	19.50%	17.51% - 21.57%	79.3%

Note: I^2 measures the percentage of total variability that was due to between survey heterogeneity

Sexual behaviours and sociodemographic factors associated with adolescent pregnancy

Supplementary Table 1 summarises the sexual behaviour and sociodemographic characteristics according to the number and percentage of adolescents pregnant at the time of the survey. Results of unadjusted analysis of the association between sociodemographic factors, sexual behaviours and adolescent pregnancy are presented in Supplementary Table 2 and Supplementary Table 3, respectively.

In the overall sample, adolescent age, place of residence, household size, literacy level, household wealth, number of previous sex partners, and knowledge of contraception were the factors associated with adolescent pregnancy after adjusting for confounders (Table 3 and Table 4). Middle adolescents (age 15-17 years) were 70% less likely to become pregnant than late adolescents (aOR=0.30, 95%CI=0.23 - 0.39). Adolescents in urban areas were less likely to become pregnant than those in rural areas (aOR=0.56, 95%CI=0.43 - 0.74). The odds of adolescent pregnancy was lower among adolescents in large households (>4 household members) than among those in small households (aOR=0.62, 95%CI=0.49 - 0.78). There was a dose-response relationship between literacy level and adolescent pregnancy. Semi-literates (aOR=0.56, 95%CI=0.39 - 0.82) and literates (aOR=0.28, 95%CI=0.21 - 0.37) were less likely to become pregnant than illiterate. Adolescents who were not working had a reduced likelihood (aOR=0.62, 95%CI=0.43 - 0.90) of becoming pregnant than those who worked. There was a dose-response relationship between household income and adolescent pregnancy. Those from middle- (aOR=0.91, 95%CI=0.67 - 1.24) and high- (aOR=0.59, 95%CI=0.36 - 0.94) income households were less likely to become pregnant compared to those from low-income households. Similarly, a dose-response relationship was found between the number of previous sex partners and adolescent pregnancy. Adolescents with one (aOR=0.46, 0.23 - 0.95) or two (aOR=0.67, 95%CI=0.33 - 1.36) previous sex partners were less likely to become pregnant than those with three or more previous partners. Adolescents who were unaware of contraceptive methods were less likely (aOR=0.49, 95%CI=0.27 - 0.90) to become pregnant than those who knew about modern or traditional methods.

However, not all the determinants identified in the overall sample were consistently associated with adolescent pregnancy in the last three decades. Between 1988 and 1998, adolescent age, literacy level, employment status, household size, and whether the mother was alive were the only factors associated with adolescent pregnancy in the adjusted analysis (Table 3 and Table 4). Middle adolescents were less likely to become pregnant than late adolescents. Semi-literate or literate adolescents were less likely to become pregnant than illiterate. Those who had not worked in the previous 12 months were less likely than those who had worked to become pregnant. Adolescents in large households were less likely to become pregnant than those in small households. Adolescents whose mothers were still alive were less likely to experience pregnancy than those whose mothers had passed away.

For peer review only - http://bmjopen.bmj.com/site/about/guidelines.xhtml

Between 2003 and 2008, adolescent age, literacy level, household size, household wealth, age of last sexual partner, number of previous sex partners, and knowledge of contraception were the factors associated with adolescent pregnancy after adjusting for potential confounders (Table 3 and Table 4). Adolescents in middle and high-income families were less likely than those in low-income families to become pregnant. Middle adolescents were less likely to become pregnant than late adolescents. Adolescent pregnancy was less likely among those in large households than those in small households. Semi-literates or literates were less likely to become pregnant than illiterate. Those whose last sexual partner was an older person were less likely to become pregnant than those whose last partner was an adolescent. Adolescent pregnancy was less likely among those who had one or two previous sex partners compared to those who had three or more previous partners. Those unaware of contraceptive methods had reduced odds of getting pregnant than those who knew about modern or traditional methods.

From 2011 to 2019, adolescent age, place of residence, literacy level, and knowledge of the fertility period during menstruation were the factors associated with adolescent pregnancy after adjusting for other sexual behaviours and sociodemographic factors (Table 3 and Table 4). Middle adolescents were less likely to become pregnant than late adolescents. Adolescents in urban areas were about 50% less likely than those in rural areas to become pregnant. Semi-literates and literates were less likely to become pregnant than illiterate. Adolescents who believed the most fertile period was just before or during menstruation or right after it were less likely to become pregnant than those who said it was halfway between two menstrual periods.

Table 3 Results of adjusted logistic regression analysis of the association between sociodemographic characteristics and adolescent pregnancy, 1988-2019

Survey years	1988 - 1998	2003 - 2008	2011 - 2019	Overall sample (1988-2019)
Population analysed (n)	2541	3350	8665	14556
Number currently pregnant	96	117	282	495
	**Adjusted odds ratio (95% CI)**	**Adjusted odds ratio (95% CI)**	**Adjusted odds ratio (95% CI)**	**Adjusted odds ratio (95% CI)**
**Age**	P < 0.001	P < 0.001	P < 0.001	P < 0.001
15 - 17	0.40 (0.24 - 0.66)	0.39 (0.24 - 0.64)	0.22 (0.15 - 0.33)	0.30 (0.23 - 0.39)
18 - 19	1.00	1.00	1.00	1.00
**Place of residence**	P = 0.23	P = 0.72	P = 0.002	P < 0.001
Urban	0.74 (0.45 - 1.22)	0.86 (0.40 - 1.89)	0.54 (0.36 - 0.81)	0.56 (0.43 - 0.74)
Rural	1.00	1.00	1.00	1.00
**Sex of household head**	P = 0.23	P = 0.06	P = 0.60	P = 0.42
Male	1.61 (0.73 - 3.54)	0.61 (0.36 - 1.02)	0.91 (0.63 - 1.31)	0.89 (0.66 - 1.19)
Female	1.00	1.00	1.00	1.00
**Number of household members**	P = 0.001	P = 0.02	P = 0.19	P < 0.001
≤4 members	1.00	1.00	1.00	1.00
>4 members	0.46 (0.29 - 0.73)	0.58 (0.37 - 0.91)	0.78 (0.54 - 1.13)	0.62 (0.49 - 0.78)
**Mother still alive**	P = 0.07		P = 0.48	P = 0.94

For peer review only - http://bmjopen.bmj.com/site/about/guidelines.xhtml

Yes	0.30 (0.08 - 1.09)	--	0.58 (0.13 - 2.58)	0.94 (0.24 - 3.77)
No	1.00	--	1.00	1.00
**Father still alive**	P = 0.85	P = 0.86	P = 0.99	P = 0.84
Yes	1.00	1.00	1.00	1.00
No	1.12 (0.36 - 3.44)	1.13 (0.30 - 4.21)	1.00 (0.40 - 2.51)	1.08 (0.52 - 2.24)
**Literacy level**	P = 0.001*	P < 0.001*	P < 0.001*	P < 0.001*
Illiterate	1.00	1.00	1.00	1.00
Semi-literate	0.55 (0.20 - 1.50)	0.42 (0.22 - 0.81)	0.66 (0.39 - 1.13)	0.56 (0.39 - 0.82)
Literate	0.40 (0.23 - 0.69)	0.27 (0.16 - 0.45)	0.27 (0.17 - 0.42)	0.28 (0.21 - 0.37)
**Employment**	P = 0.05	P = 0.29	P = 0.12	P = 0.01
Yes	1.00	1.00	1.00	1.00
No	0.61 (0.37 - 1.01)	0.73 (0.41 - 1.31)	0.59 (0.30 - 1.15)	0.62 (0.43 - 0.90)
**Household income**	P = 0.56	P < 0.001*	P = 0.27	P = 0.03*
Low income	1.00	1.00	1.00	1.00
Middle income	0.67 (0.32 - 1.39)	0.65 (0.39 - 1.09)	1.09 (0.70 - 1.69)	0.91 (0.67 - 1.24)
High income	0.88 (0.45 - 1.74)	0.30 (0.15 - 0.58)	0.63 (0.32 - 1.26)	0.59 (0.36 - 0.94)
**Frequency of media exposure**		P = 0.82	P = 0.23	P = 0.51
Not at all	--	1.00	1.00	1.00
Less than once a week	--	1.40 (0.45 - 4.37)	1.29 (0.62 - 2.69)	1.25 (0.68 - 2.32)
At least once a week	--	1.04 (0.49 - 2.20)	1.70 (0.91 - 3.18)	1.34 (0.82 - 2.20)

Table 4 Results of adjusted logistic regression analysis of the association between sexual behaviours and adolescent pregnancy, 1988-2019.

Survey years	1988 - 1998	2003 - 2008	2011 - 2019	Overall sample (1988-2019)
Population analysed (n)	2541	3350	8665	14556
Number currently pregnant	96	117	282	495
	**Adjusted odds ratio (95% CI)**	**Adjusted odds ratio (95% CI)**	**Adjusted odds ratio (95% CI)**	**Adjusted odds ratio (95% CI)**
**Age at first sex**	P = 0.63	P = 0.79	P = 0.10	P = 0.14
Before age 16	0.85 (0.45 - 1.63)	0.91 (0.44 - 1.88)	0.63 (0.36 - 1.10)	0.75 (0.52 - 1.10)
16 - 19 years	1.00	1.00	1.00	1.00
**Number of previous sex partners**		P = 0.03*	P = 0.79	P = 0.04*
1	--	0.29 (0.10 - 0.88)	0.73 (0.27 - 1.95)	0.46 (0.23 - 0.95)
2	--	0.56 (0.21 - 1.52)	0.90 (0.31 - 2.58)	0.67 (0.33 - 1.36)
Three or more	--	1.00	1.00	1.00
**Age of last sexual partner**		P = 0.02	P = 0.20	P = 0.39

For peer review only - http://bmjopen.bmj.com/site/about/guidelines.xhtml

Below 20 years	--	1.00	1.00	1.00
More than 20 years	--	0.14 (0.03 - 0.76)	2.00 (0.69 - 5.81)	1.46 (0.61 - 3.48)
**Knowledge of contraception**	P = 0.20	P = 0.08		P = 0.02
Knows no method	0.62 (0.30 - 1.29)	0.36 (0.11 - 1.15)	--	0.49 (0.27 - 0.90)
Knows modern or traditional method	1.00	1.00	--	1.00
**knowledge of fertility period during menstruation**	P = 0.56	P = 0.45	P = 0.02	P = 0.83
Just before or during menstrual period	2.09 (0.50 - 8.67)	1.88 (0.70 - 5.05)	0.17 (0.04 - 0.69)	0.90 (0.45 - 1.77)
Right after menstrual period or at any time	0.95 (0.42 - 2.13)	1.24 (0.60 - 2.56)	0.51 (0.24 - 1.07)	0.87 (0.56 - 1.36)
Halfway between two menstrual periods	1.00	1.00	1.00	1.00

Discussion

Based on a meta-analysis of eleven nationally representative surveys conducted between 1988 and 2019, the prevalence of adolescent pregnancy was 15.4%, with substantial regional variation. The prevalence was higher in rural areas than in urban areas. Over the last three decades, adolescent pregnancy peaked in 1988, followed by a progressive decline in the 1990s and early 2000s; but the prevalence has slowly increased over the last decade. The pooled data suggest that the prevalence of adolescent pregnancy rose with increasing adolescent age, a pattern consistent across all eleven surveys. The prevalence reported in this study is lower than the 18.8%, 19.3%, and 17.7% prevalence estimates for Africa, sub-Saharan Africa, and West Africa, respectively (3).

In line with the present findings, studies in Indonesia and Uganda reported a higher likelihood of pregnancy among teenagers living in rural areas, those with no or low education, and adolescents in low-income households(19,20). Similarly, in a systematic review of 24 studies in sub-Saharan Africa, about half of the included studies reported a higher likelihood of adolescent pregnancy in low-income households(11). Furthermore, an analysis of the 2014–2015 Rwanda Demographic and Health Survey confirmed that adolescents from large families were less likely than those from small families to get pregnant(21). Similar to the findings of this study, Jonas et al., found higher odds of adolescent pregnancy among those with more than one sex partner in South Africa(22). Though there was no association between adolescent age and pregnancy in the south African study, in a pooled analysis of data from five African countries and two cross-sectional studies in Ghana, older adolescents had higher odds of adolescent pregnancy (7,13,23). In support of the present findings, a qualitative study in Kenya found that adolescents engaged in incoming generating activities were more likely to get pregnant than those not working(24); however, a study in Ethiopia reported a contrary finding(25). In an earlier study in Ghana, there was no association between care status (orphanhood) and adolescent pregnancy(23), which agrees with the current results for 2011-2019 and the overall sample.

There are several reasons to explain the heightened vulnerability of adolescents in low-income households to pregnancy. In most cases, adolescents in these homes are not in school; instead, parents force them into early marriage to relieve the financial burden of taking care of the adolescents or prevent out-of-wedlock pregnancy(26). Several studies have revealed that most adolescent girls from poor homes

For peer review only - http://bmjopen.bmj.com/site/about/guidelines.xhtml

engage in early sexual relationships for financial and material gains because parents cannot provide their basic needs, including food, toiletries, clothes, shoes, school supplies, fees, and housing (24,27–30). For instance, in a Ugandan study, adolescents acknowledged that they sometimes offer sex to boys/men as payment for money borrowed or in return for material items because their parents cannot provide(30). Under these situations, adolescents are less able to negotiate safe sex and condom use, increasing their chance of pregnancy. Some may engage multiple sexual partners for greater financial benefits. Therefore, it is not surprising that in this study, adolescents with three or more sexual partners were more likely to experience pregnancy than those with one partner.

Additionally, some adolescents engage in all manner of income-generating activities to meet their basic needs, and this exposes them to mistreatment, sexual abuse, and exploitation, particularly those working as domestic help, bartenders, waitresses, shopkeepers, and selling at night or work that involves late-night shifts(24,30). This is probably why adolescents who worked 12 months prior to the surveys analysed in this study had a higher chance of becoming pregnant. Also, it has been suggested that some adolescents spend the income from these jobs on activities that increase their risk of pregnancy, such as alcohol, drugs, and nightclubs (24). In large families, adolescents are supervised, guided, and supported emotionally and financially not only by their parents but also by other family members to make sexual and reproductive health decisions favouring pregnancy prevention, reducing the likelihood of adolescent pregnancy in such families, as reported in this study. Also, in these families' adolescents who are uncomfortable discussing sexual health issues with their parents have other family members to consult.

The lower likelihood of pregnancy among adolescents in urban areas may be due to the advantages the urban setting offers. Urban residents have easy access to pregnancy prevention information and services, including easy access to various effective modern contraceptive methods (including emergency contraceptives) through the many hospitals, clinics, pharmacy shops, and family planning centres in urban areas. In rural areas, misconceptions are promoted, even when contraceptives are available, and sexually active adolescents usually rely on traditional contraceptive methods that are ineffective. Furthermore, traditional and cultural practices that promote early adolescent marriage are rarely practised in most urban settings, reducing adolescents' exposure to pregnancy. In addition, most adolescents in urban areas are better educated than their rural counterparts, increasing their ability to understand and effectively use sexual and reproductive health information to reduce their susceptibility to pregnancy. This further explains why adolescents who were semi-literate or literate in this study were less likely to experience pregnancy than those who were illiterate. Also, schooling delays marriage and empowers girls to make rational decisions in their relationships that prevent pregnancy(10).

Most adolescents in the present study did not know the fertile phase of the menstrual cycle. Therefore, it is surprising that those who did not know the fertile period were less likely to become pregnant. It is possible that these girls had not initiated sex or that those who correctly identified the fertile phase learned it when they became pregnant (reverse causality). This may also explain the unexpected finding on knowledge about contraceptives. However, it was not surprising that older adolescents were more likely to get pregnant than younger adolescents because most older adolescents are sexually active and engage in more sexual risk behaviours than middle adolescents. It is also possible that parents and other adults support and guide middle-aged adolescents more than they do older teenagers. In addition, the lower adolescent pregnancy among those whose last partner was older in this study is possible due to better pregnancy prevention and sexual and reproductive health knowledge of older partners.

For peer review only - http://bmjopen.bmj.com/site/about/guidelines.xhtml

Age-appropriate sexual and reproductive health education as part of the school curriculum and in communities is essential to help adolescents from all settings, income groups, and family backgrounds make rational decisions that will delay pregnancy and motherhood. Adolescents access and use of contraceptives, including emergency contraceptives, should be promoted, particularly in rural communities and low-income households. Traditional leaders and stakeholders who promote cultural and traditional practices that encourage early motherhood should be educated on the harmful effects of adolescent childbearing, and such practices discouraged. Girls should be encouraged and motivated to stay in school even when they become pregnant, considering the long-term repercussions of dropping out. In addition, interventions to reduce adolescent pregnancy should include boys and men.

The main strength of this study is the use of a large nationally representative sample that captures adolescents from various sociodemographic backgrounds and settings. The study's findings are generalisable to all adolescents in Ghana and more relevant to national policies than previous studies that mainly focused on individual communities. One major limitation of the study is the possibility of reverse causality and recall bias. To minimise the effect of reverse causality, analysis of the determinants was restricted to currently pregnant adolescents. However, it is important to note that this study was not designed for causal relationships, so I make no such claims. Furthermore, it is possible that the prevalence reported in this study was underestimated because most Ghanaian women admit to pregnancy after the first trimester. In addition, the definition of adolescent pregnancy in this study excluded abortion. Furthermore, because the study relied on secondary data, cultural and traditional practices that could potentially impact adolescent pregnancy were not considered in the analysis because data were unavailable on these practices. Future studies should address the limitations with longitudinal and qualitative studies.

Conclusion

Adolescent pregnancy has risen slowly over the last decade, following a steady prevalence between 1998 and 2008. Regional and urban-rural disparities in the prevalence were substantial, and age patterns showed that adolescent pregnancy increased as adolescents got older. The factors influencing adolescent pregnancy differed across the eras except for literacy level and adolescent age; illiteracy and older adolescent age were associated with a higher likelihood of adolescent pregnancy across the three decades from 1988 to 2019.

Ethics approval and consent to participate: The Ghana Statistical Services and DHS program obtained ethical approval, participant consent, and permission for each primary survey. MEASURE DHS granted permission and access for the secondary use of the survey data for the present analysis. Because the data is available in the public domain with no personal identifiers, ethical approval was not sought for the secondary analysis.

Consent for publication: Not applicable

Data sharing statement: All the datasets analysed are publicly available with the measure DHS program (https://dhsprogram.com) and UNICEF's MICS programme (https://mics.unicef.org/).

Competing interests: None

Funding: The author is receiving PhD studentship from the Economic and Social Research Council (ESRC) for doctoral research and training at the London School of Hygiene & Tropical Medicine, London, UK [grant

For peer review only - http://bmjopen.bmj.com/site/about/guidelines.xhtml

number: ES/P000592/1]. The ESRC had no role in this study's design, analysis of the data, or manuscript preparation.

Authors' contributions: SM conceived and designed the study, accessed the data, conducted the literature search, performed the statistical analysis, and drafted the manuscript.

Acknowledgements: I would like to thank measure DHS and UNICEF's MICS programme for prompt approval and access to the datasets for the analysis.

For peer review only

References

1. UNFPA. ADOLESCENT PREGNANCY : A Review of the Evidence [Internet]. New York, USA; 2013. Available from: https://www.unfpa.org/adolescent-pregnancy

2. World Health Organization. Adolescent pregnancy [Internet]. 2022 [cited 2023 Feb 20]. Available from: https://www.who.int/en/news-room/fact-sheets/detail/adolescent-pregnancy

3. Kassa GM, Arowojolu AO, Odukogbe AA, Yalew AW. Prevalence and determinants of adolescent pregnancy in Africa: A systematic review and Meta-analysis 11 Medical and Health Sciences 1117 Public Health and Health Services. Reprod Health. 2018;15(1):1–17.

4. Unicef. Early Childbearing [Internet]. 2021 [cited 2022 Sep 6]. Available from: https://data.unicef.org/topic/child-health/adolescent-health/

5. UNFPA. Girlhood, not motherhood: Preventing adolescent pregnancy [Internet]. United Nations Population Fund. New York, USA; 2015. Available from: https://www.unfpa.org/sites/default/files/pub-pdf/Girlhood_not_motherhood_final_web.pdf

6. Yussif AS, Lassey A, Ganyaglo GYK, Kantelhardt EJ, Kielstein H. The long-term effects of adolescent pregnancies in a community in Northern Ghana on subsequent pregnancies and births of the young mothers. Reprod Health. 2017;14(1):1–7.

7. Tetteh J, Nuertey BD, Dwomoh D, Udofia EA, Mohammed S, Adjei-Mensah E, et al. Teenage pregnancy and experience of physical violence among women aged 15-19 years in five African countries: Analysis of complex survey data. PLoS One [Internet]. 2020;15(10 October):1–18. Available from: http://dx.doi.org/10.1371/journal.pone.0241348

8. Dzotsi HT, Oppong Asante K, Osafo J. Challenges associated with teenage motherhood in Ghana: a qualitative study. Vulnerable Child Youth Stud [Internet]. 2020;15(1):85–96. Available from: https://doi.org/10.1080/17450128.2020.1719251

9. Gbogbo S. Early motherhood: voices from female adolescents in the Hohoe Municipality, Ghana—a qualitative study utilizing Schlossberg's Transition Theory. Int J Qual Stud Health Well-being [Internet]. 2020;15(1). Available from: https://doi.org/10.1080/17482631.2020.1716620

10. UNFPA. Adolescent pregnancy: A matter of human rights [Internet]. 2017 [cited 2022 Jan 2]. Available from: https://www.unfpa.org/adolescent-pregnancy#readmore-expand

11. Yakubu I, Salisu WJ. Determinants of adolescent pregnancy in sub-Saharan Africa: A systematic review. Reprod Health. 2018;15(1).

12. Gunawardena N, Fantaye AW, Yaya S. Predictors of pregnancy among young people in sub-Saharan Africa: A systematic review and narrative synthesis. BMJ Glob Heal. 2019;4(3):1–8.

13. Asare BYA, Baafi D, Dwumfour-Asare B, Adam AR. Factors associated with adolescent pregnancy in the Sunyani Municipality of Ghana. Int J Africa Nurs Sci. 2019;10(February):87–91.

14. Bedzo JY, Manortey S. Factors Influencing Teenage Pregnancy in the Lower Manya Krobo Municipality in the Eastern Region of Ghana: A Cross-Sectional Study. OALib. 2019;06(12):1–17.

15. The DHS Program. Publications Catalog [Internet]. 2022 [cited 2022 Feb 4]. Available from: https://dhsprogram.com/Publications/Publication-Search.cfm?ctry_id=14&country=Ghana

16. United Nations Population Division. Population Fact Sheet No. 2: Household size and composition around the world [Internet]. 2017. Available from: http://www.un.org/en/development/desa/population/publications/pdf/popfacts/PopFacts_2017-2.pdf

17. Amoateng AY, Ewemooje OS, Biney E. Prevalence and determinants of adolescent pregnancy among women of reproductive age in South Africa. Afr J Reprod Health. 2022;26(1):82–91.

18. Higgins JPT, Thompson SG. Quantifying heterogeneity in a meta-analysis. Stat Med. 2002;21(11):1539–58.

19. Gayatri M, Gayatri M, Permata J, Kusuma HP, Timur J. Socioeconomic determinants of adolescent pregnancy in Indonesia : A population-based survey Key Messages : Ann Trop Med Public Heal. 2021;24(01).

20. Byonanebye J, Brazauskas R, Tumwesigye N, Young S, May T, Cassidy L. Geographic variation and risk factors for teenage pregnancy in Uganda. Afr Health Sci. 2020;20(4):1898–907.

21. Uwizeye D, Muhayiteto R, Kantarama E, Wiehler S, Murangwa Y. Prevalence of teenage pregnancy and the associated contextual correlates in Rwanda. Heliyon [Internet]. 2020;6(10):e05037. Available from: https://doi.org/10.1016/j.heliyon.2020.e05037

22. Jonas K, Crutzen R, Van Den Borne B, Sewpaul R, Reddy P. Teenage pregnancy rates and associations with other health risk behaviours: A three-wave cross-sectional study among South African school-going adolescents. Reprod Health [Internet]. 2016;13(1). Available from: http://dx.doi.org/10.1186/s12978-016-0170-8

23. Morhe ESK, Tagbor HK, Ankobea FK, Danso KA. Reproductive experiences of teenagers in the Ejisu-Juabeng district of Ghana. Int J Gynecol Obstet [Internet]. 2012;118(2):137–40. Available from: http://dx.doi.org/10.1016/j.ijgo.2012.03.035

24. Juma M, Alaii J, Bartholomew LK, Askew I, Van Den Born B. Understanding orphan and non-orphan adolescents' sexual risks in the context of poverty: A qualitative study in Nyanza Province, Kenya. BMC Int Health Hum Rights. 2013;13(32).

25. Alemayehu T, Haider J, Habte D. Determinants of adolescent fertility in Ethiopia [Internet]. Vol. 24, The Ethiopian Journal of Health Development. 2010. p. 30–8. Available from: http://search.ebscohost.com/login.aspx?direct=true&AuthType=cookie,ip,shib&db=awn&AN=ejh

For peer review only - http://bmjopen.bmj.com/site/about/guidelines.xhtml

d-62942&site=ehost-live

26. UNICEF. Early marriage: child spouses. [Internet]. Innocenti Digest. Florence, Italy Piazza; 2001. Available from: https://www.unicef-irc.org/publications/pdf/digest7e.pdf

27. McCleary-Sills J, Douglas Z, Rwehumbiza A, Hamisi A, Mabala R. Gendered norms, sexual exploitation and adolescent pregnancy in rural Tanzania. Reprod Health Matters [Internet]. 2013;21(41):97–105. Available from: http://dx.doi.org/10.1016/S0968-8080(13)41682-8

28. Krugu JK, Mevissen FEF, Prinsen A, Ruiter RAC. Who's that girl? A qualitative analysis of adolescent girls' views on factors associated with teenage pregnancies in Bolgatanga, Ghana. Reprod Health [Internet]. 2016;13(1):1–12. Available from: http://dx.doi.org/10.1186/s12978-016-0161-9

29. Zakayo MO, Lwelamira J. Sexual Behaviours among Adolescents in Community Secondary Schools in Rural Areas of Central Tanzania: A Case of Bahi District in Dodoma Region. Curr Res J Soc Sci. 2011;3(4):374–80.

30. Nabugoomu J, Seruwagi GK, Hanning R. What can be done to reduce the prevalence of teen pregnancy in rural Eastern Uganda?: Multi-stakeholder perceptions. Reprod Health. 2020;17(134):1–12.

Figure Legend/Caption

Figure 1: National prevalence of adolescent pregnancy, 1988 - 2019

Figure 2: Time trends of adolescent pregnancy by (A) survey year (B) household income (C) literacy level (D) adolescent age (E) residence (F) ethnic group

For peer review only - http://bmjopen.bmj.com/site/about/guidelines.xhtml

For peer review only

For peer review only

For peer review only - http://bmjopen.bmj.com/site/about/guidelines.xhtml

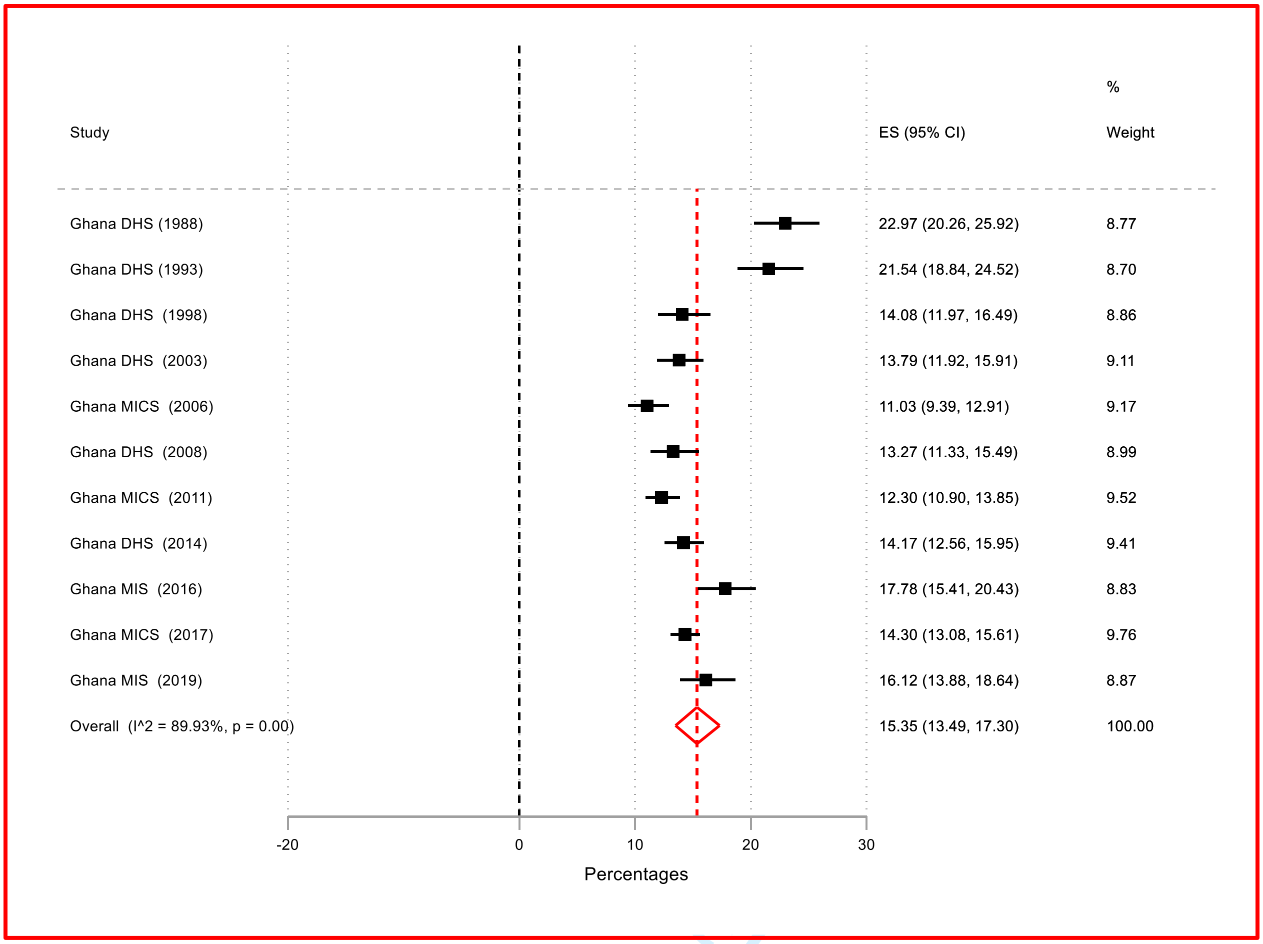

Figure 1: National prevalence of adolescent pregnancy, 1988 - 2019

For peer review only

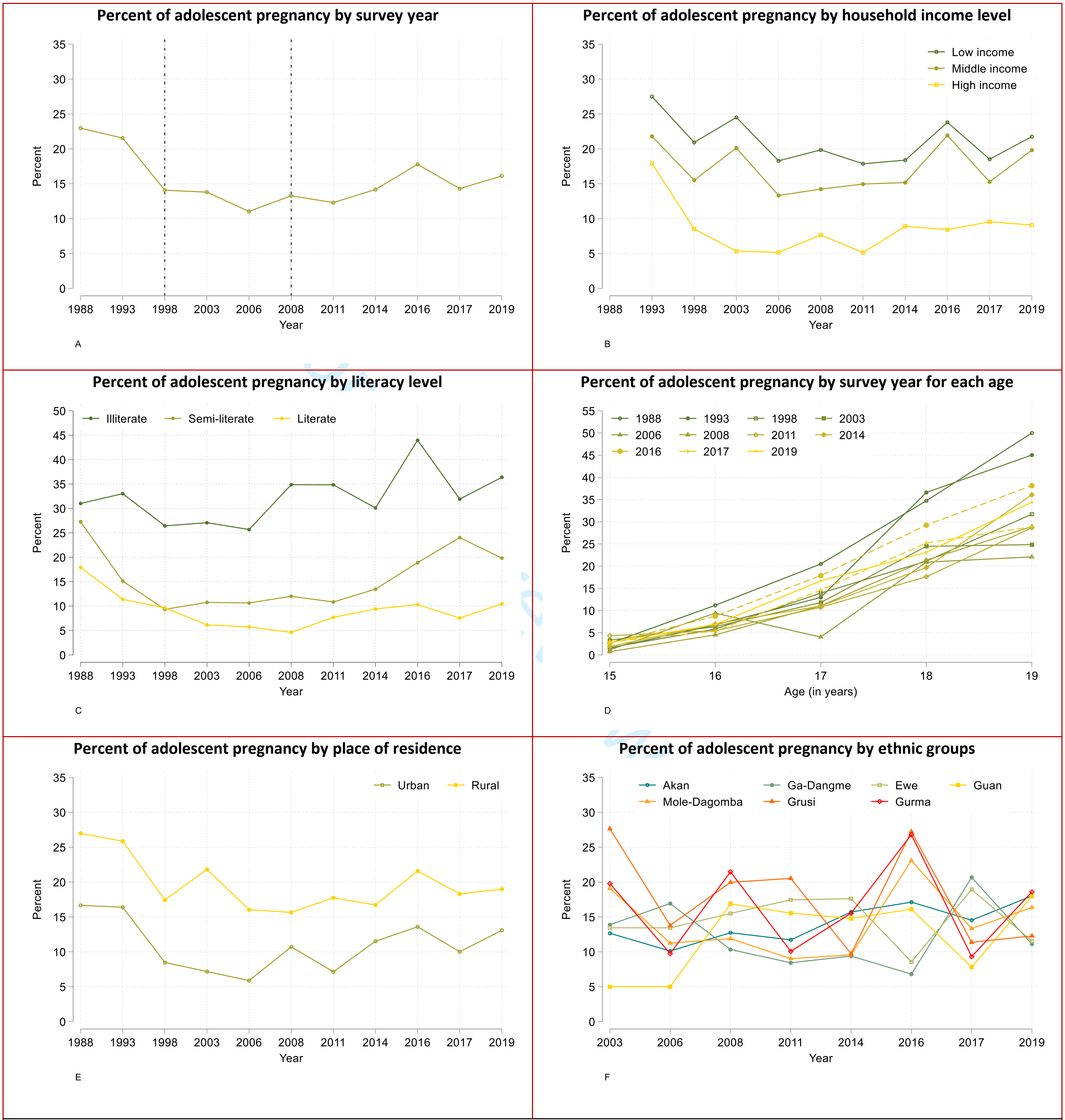

Figure 2: Time trends of adolescent pregnancy by (A) survey year (B) household income (C) literacy level (D) adolescent age (E) residence (F) ethnic group

Supplementary Figure 1. Conceptual model of factors associated with adolescent pregnancy

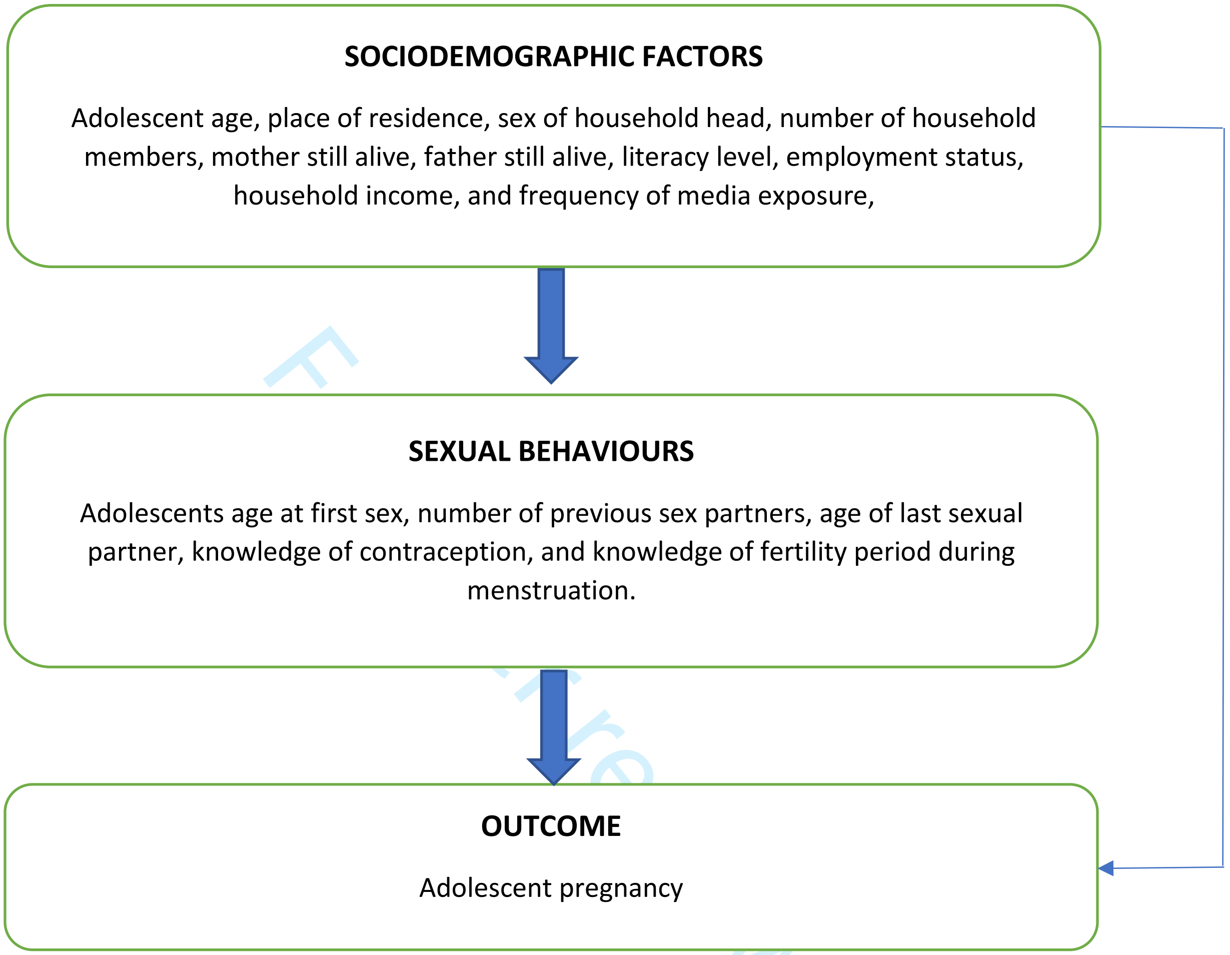

For peer review only - http://bmjopen.bmj.com/site/about/guidelines.xhtml

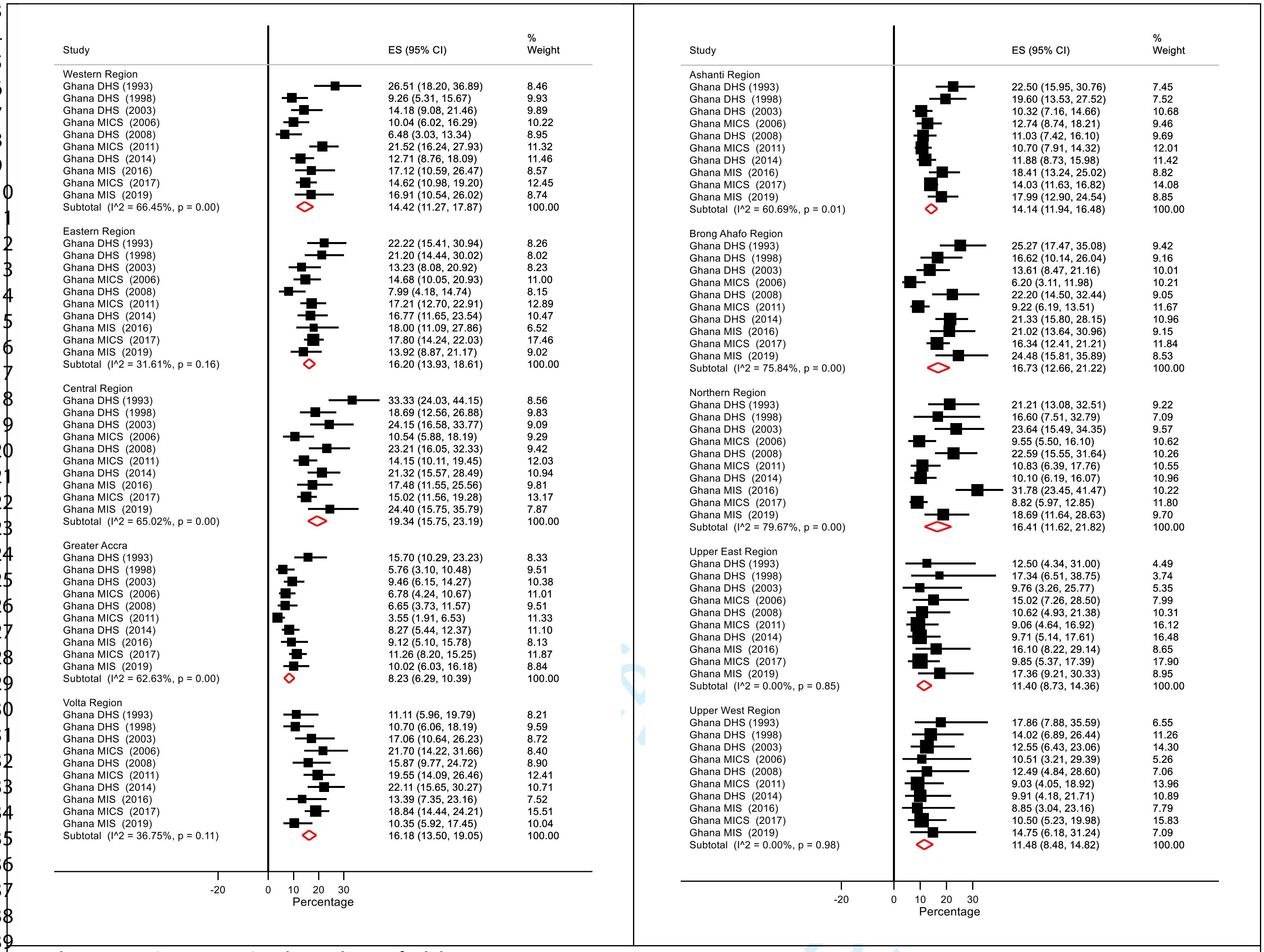

Supplementary Figure 2: Regional prevalence of adolescent pregnancy, 1993 - 2019

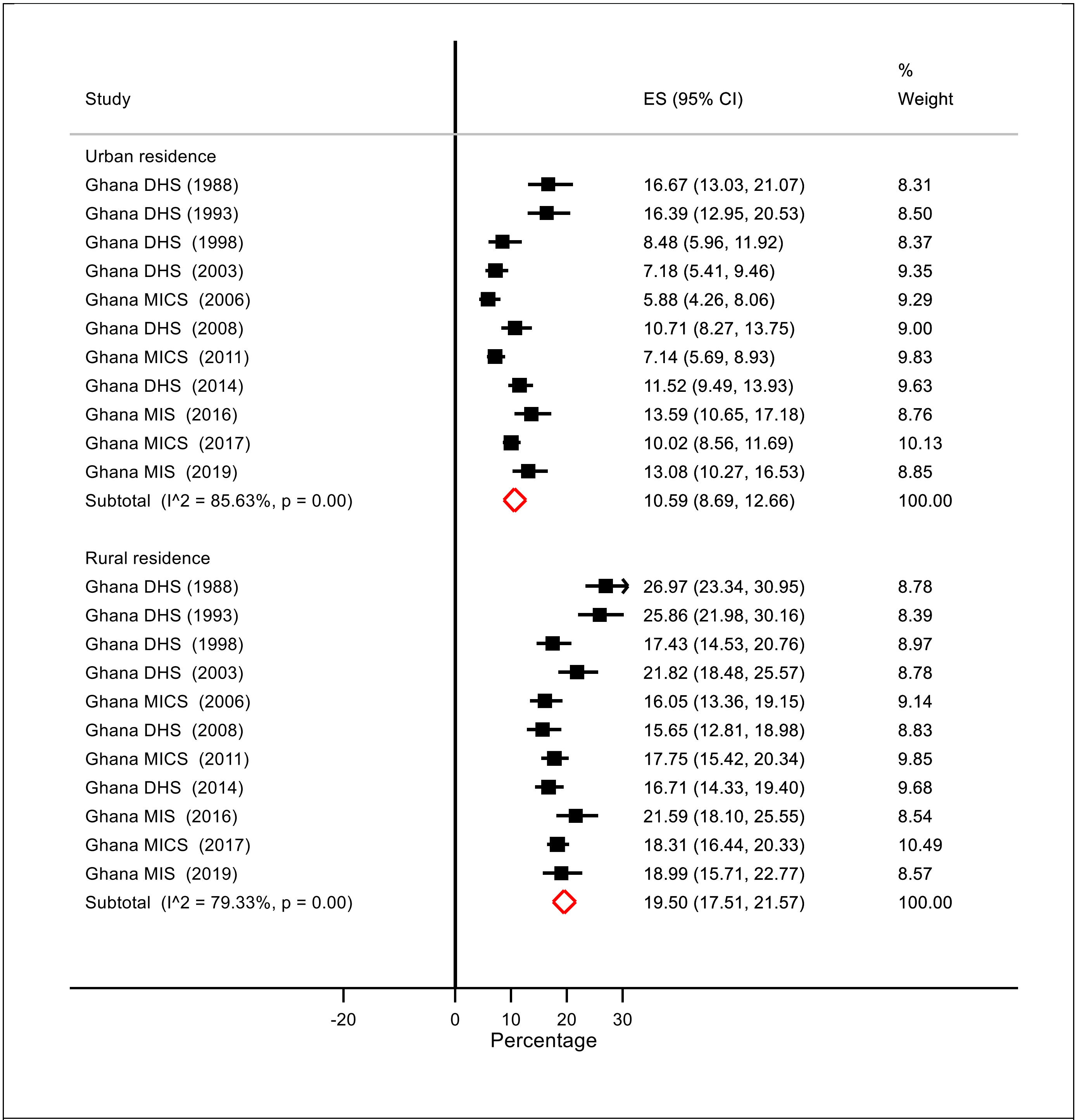

Supplementary Figure 3: Urban-rural prevalence of adolescent pregnancy , 1988 - 2019

Supplementary Table 1 Characteristics of the study population according to number and percent currently pregnant, 1988 to 2019.

Survey years	1988 - 1998		2003 - 2008		2011 - 2019	
Population analysed (unweighted)	2541		8665		8383	
Number (unweighted) currently pregnant	96		117		282	
Percent (weighted) currently pregnant	3.4%		3.7%		3.7%	
Mean age (SD), years	16.9 (0.03)		16.9 (0.03)		16.9 (0.03)	
	n	%	n	%	n	%
**Age**						
15 - 17	27	1.9	40	2.2	87	1.8
18 - 19	69	5.6	77	6.2	195	7.1
**Place of residence**						
Urban	27	2.6	30	2.2	85	2.6
Rural	69	3.9	87	5.3	197	4.8
**Sex of household head**						
Male	35	3.0	66	3.2	191	3.9
Female	26	2.6	51	4.6	91	3.4
**Number of household members**						
≤4 members	54	5.5	61	5.7	102	4.8
>4 members	42	2.5	56	2.8	180	3.2
**Mother still alive**						
Yes	25	12.9	23	2.2	59	1.8
No	4	33.3	0	0.0	3	1.7
**Father still alive**						
Yes	24	14.0	18	2.0	49	1.7
No	5	15.2	4	2.4	13	2.8
**Literacy level**						
Illiterate	62	6.1	81	8.6	119	8.7
Semi-literate	7	2.8	14	3.2	38	4.7
Literate	27	2.0	21	1.7	68	2.1
**Employment**						
Yes	48	5.9	49	7.3	27	5.9
No	48	2.4	37	2.7	33	2.8
**Household income**						
Low income	26	3.9	71	6.6	178	5.1
Middle income	13	2.2	28	4.4	56	3.8
High income	22	2.3	18	1.5	48	2.2
**Frequency of media exposure**						
Not at all	-	-	15	7.0	70	3.6
Less than once a week	-	-	11	7.7	22	2.6
At least once a week	-	-	58	3.4	114	3.6
**Age at first sex**						
≤14 years	19	6.6	17	9.7	48	7.7

For peer review only

≥15 years	64	7.2	81	9.4	140	8.6
**Number of previous sex partners**						
1	-	-	23	8.2	36	8.2
2	-	-	11	14.8	16	9.8
Three or more	-	-	6	24.0	8	11.3
**Age of last sexual partner**						
Below 20 years	-	-	5	9.3	14	4.7
More than 20 years	-	-	2	1.1	54	7.2
**Knowledge of contraception**						
Knows no method	14	2.7	4	2.5	-	-
Knows modern or traditional method	55	4.9	82	4.2	-	-
**knowledge of fertility period during menstruation**						
Just before or during menstrual period	4	8.1	12	5.4	4	1.3
Right after menstrual period or at any time	18	5.9	42	5.1	27	3.9
Halfway between two menstrual periods	13	6.1	12	3	23	6.8

For peer review only

Supplementary Table 2 Results of unadjusted logistic regression analysis of the association between sociodemographic characteristics and adolescent pregnancy, 1988-2019

Survey years	1988 - 1998	2003 - 2008	2011 - 2019	Overall sample (1988-2019)
Population analysed (n)	2541	3350	8665	14556
Number currently pregnant	96	117	282	495
	**Unadjusted odds ratio (95% CI)**	**Unadjusted odds ratio (95% CI)**	**Unadjusted odds ratio (95% CI)**	**Unadjusted odds ratio (95% CI)**
**Age**	P < 0.001	P < 0.001	P < 0.001	P < 0.001
15 - 17	1.00	1.00	1.00	1.00
18 - 19	3.08 (1.87 - 5.08)	2.95 (1.86 - 4.68)	4.23 (2.98 - 6.00)	3.62 (2.83 - 4.64)
**Place of residence**	P = 0.08	P < 0.001	P < 0.001	P < 0.001
Urban	0.64 (0.39 - 1.06)	0.40 (0.24 - 0.66)	0.53 (0.38 - 0.74)	0.52 (0.41 - 0.66)
Rural	1.00	1.00	1.00	1.00
**Sex of household head**	P = 0.68	P = 0.09	P = 0.37	P = 0.89
Male	1.00	1.00	1.00	1.00
Female	0.87 (0.46 - 1.67)	1.45 (0.94 - 2.25)	0.86 (0.61 - 1.20)	0.98 (0.76 - 1.27)
**Number of household members**	P < 0.001	P < 0.001	P = 0.01	P < 0.001
≤4 members	1.00	1.00	1.00	1.00
>4 members	0.43 (0.28 - 0.67)	0.48 (0.32 - 0.71)	0.65 (0.47 - 0.90)	0.56 (0.45 - 0.69)
**Mother still alive**	P = 0.07		P = 0.95	P = 0.53
Yes	0.30 (0.08 - 1.10)	--	1.04 (0.25 - 4.37)	0.76 (0.33 - 1.77)
No	1.00	--	1.00	1.00
**Father still alive**	P = 0.86	P = 0.74	P = 0.15	P = 0.16
Yes	1.00	1.00	1.00	1.00
No	1.10 (0.37 - 3.24)	1.23 (0.36 - 4.19)	1.69 (0.83 - 3.41)	1.47 (0.87 - 2.48)
**Literacy level**	P < 0.001	P < 0.001	P < 0.001	P < 0.001
Illiterate	1.00	1.00	1.00	1.00
Semi-literate	0.44 (0.18 - 1.07)	0.36 (0.19 - 0.68)	0.52 (0.30 - 0.89)	0.48 (0.33 - 0.70)
Literate	0.31 (0.19 - 0.51)	0.18 (0.11 - 0.29)	0.22 (0.15 - 0.34)	0.24 (0.18 - 0.31)
**Employment**	P < 0.001	P < 0.001	P = 0.02	P < 0.001
Yes	1.00	1.00	1.00	1.00
No	0.40 (0.25 - 0.62)	0.35 (0.21 - 0.58)	0.46 (0.24 - 0.88)	0.39 (0.29 - 0.52)
**Household income**	P = 0.13	P < 0.001	P < 0.001	P < 0.001
Low income	1.00	1.00	1.00	1.00
Middle income	0.56 (0.27 - 1.15)	0.65 (0.41 - 1.03)	0.74 (0.50 - 1.09)	0.70 (0.53 - 0.93)
High income	0.57 (0.31 - 1.05)	0.22 (0.13 - 0.38)	0.42 (0.27 - 0.63)	0.37 (0.27 - 0.50)

For peer review only

**Frequency of media exposure**		P = 0.02	P = 0.50	P = 0.73
Not at all	--	2.10 (1.10 - 4.01)	0.99 (0.65 - 1.52)	1.13 (0.79 - 1.61)
Less than once a week	--	2.33 (0.94 - 5.74)	0.72 (0.41 - 1.26)	0.93 (0.58 - 1.49)
At least once a week	--	1.00	1.00	1.00

For peer review only

Supplementary Table 3 Results of unadjusted logistic regression analysis of the association between sexual behaviours and adolescent pregnancy, 1988-2019.

Survey years	1988 - 1998	2003 - 2008	2011 - 2019	Overall sample (1988-2019)
Population analysed (n)	2541	3350	8665	14556
Number currently pregnant	96	117	282	495
	**Unadjusted odds ratio (95% CI)**	**Unadjusted odds ratio (95% CI)**	**Unadjusted odds ratio (95% CI)**	**Unadjusted odds ratio (95% CI)**
**Age at first sex**	P = 0.79	P = 0.0.91	P = 0.56	P = 0.61
≤14 years	0.92 (0.49 - 1.71)	1.04 (0.53 - 2.03)	0.88 (0.57 - 1.36)	0.92 (0.68 - 1.26)
≥15 years	1.00	1.00	1.00	1.00
**Number of previous sex partners**		P = 0.06	P = 0.72	P = 0.08
1	-	0.28 (0.10 - 0.83)	0.70 (0.28 - 1.75)	0.46 (0.23 - 0.93)
2	-	0.55 (0.20 - 1.53)	0.85 (0.31 - 2.31)	0.69 (0.34 - 1.41)
Three or more	-	1.00	1.00	1.00
**Age of last sexual partner**		P = 0.02	P = 0.26	P = 0.53
Below 20 years	-	1.00	1.00	1.00
More than 20 years	-	0.11 (0.02 - 0.65)	1.56 (0.72 - 3.37)	1.24 (0.63 - 2.45)
**Knowledge of contraception**	P = 0.09	P = 0.34		P = 0.06
Knows no method	0.55 (0.27 - 1.10)	0.57 (0.18 - 1.79)	-	0.57 (0.32 - 1.03)
Knows modern or traditional method	1.00	1.00	-	1.00
**knowledge of fertility period during menstruation**	P = 0.88	P = 0.23		P = 0.78
Just before or during menstrual period	1.35 (0.36 - 5.13)	1.85 (0.74 - 4.61)	-	0.81 (0.44 - 1.52)
Right after menstrual period or at any time	0.97 (0.44 - 2.16)	1.75 (0.90 - 3.39)	-	1.00 (0.67 - 1.49)
Halfway between two menstrual periods	1.00	1.00	-	1.00

For peer review only

STROBE Statement—Checklist of items that should be included in reports of ***cross-sectional studies***

	Item No	Recommendation	Page No
**Title and abstract**	1	(*a*) Indicate the study's design with a commonly used term in the title or the abstract	1
		(*b*) Provide in the abstract an informative and balanced summary of what was done and what was found	2
**Introduction**			
Background/rationale	2	Explain the scientific background and rationale for the investigation being reported	3
Objectives	3	State specific objectives, including any prespecified hypotheses	3
**Methods**			
Study design	4	Present key elements of study design early in the paper	3
Setting	5	Describe the setting, locations, and relevant dates, including periods of recruitment, exposure, follow-up, and data collection	4
Participants	6	(*a*) Give the eligibility criteria, and the sources and methods of selection of participants	4
Variables	7	Clearly define all outcomes, exposures, predictors, potential confounders, and effect modifiers. Give diagnostic criteria, if applicable	4
Data sources/ measurement	8*	For each variable of interest, give sources of data and details of methods of assessment (measurement). Describe comparability of assessment methods if there is more than one group	*4*
Bias	9	Describe any efforts to address potential sources of bias	4
Study size	10	Explain how the study size was arrived at	4
Quantitative variables	11	Explain how quantitative variables were handled in the analyses. If applicable, describe which groupings were chosen and why	4
Statistical methods	12	(*a*) Describe all statistical methods, including those used to control for confounding	4
		(*b*) Describe any methods used to examine subgroups and interactions	4
		(*c*) Explain how missing data were addressed	4
		(*d*) If applicable, describe analytical methods taking account of sampling strategy	4
		(*e*) Describe any sensitivity analyses	NA
**Results**			
Participants	13*	(a) Report numbers of individuals at each stage of study—eg numbers potentially eligible, examined for eligibility, confirmed eligible, included in the study, completing follow-up, and analysed	5
		(b) Give reasons for non-participation at each stage	
		(c) Consider use of a flow diagram	
Descriptive data	14*	(a) Give characteristics of study participants (eg demographic, clinical, social) and information on exposures and potential confounders	5
		(b) Indicate number of participants with missing data for each variable of interest	
Outcome data	15*	Report numbers of outcome events or summary measures	5
Main results	16	(*a*) Give unadjusted estimates and, if applicable, confounder-adjusted estimates and their precision (eg, 95% confidence interval). Make clear which confounders were adjusted for and why they were included	5-6

For peer review only - http://bmjopen.bmj.com/site/about/guidelines.xhtml

		(*b*) Report category boundaries when continuous variables were categorized	NA
		(*c*) If relevant, consider translating estimates of relative risk into absolute risk for a meaningful time period	
Other analyses	17	Report other analyses done—eg analyses of subgroups and interactions, and sensitivity analyses	NA
**Discussion**			
Key results	18	Summarise key results with reference to study objectives	6
Limitations	19	Discuss limitations of the study, taking into account sources of potential bias or imprecision. Discuss both direction and magnitude of any potential bias	8-9
Interpretation	20	Give a cautious overall interpretation of results considering objectives, limitations, multiplicity of analyses, results from similar studies, and other relevant evidence	7
Generalisability	21	Discuss the generalisability (external validity) of the study results	8-9
**Other information**			
Funding	22	Give the source of funding and the role of the funders for the present study and, if applicable, for the original study on which the present article is based	10

*Give information separately for exposed and unexposed groups.

Note: An Explanation and Elaboration article discusses each checklist item and gives methodological background and published examples of transparent reporting. The STROBE checklist is best used in conjunction with this article (freely available on the Web sites of PLoS Medicine at http://www.plosmedicine.org/, Annals of Internal Medicine at http://www.annals.org/, and Epidemiology at http://www.epidem.com/). Information on the STROBE Initiative is available at www.strobe-statement.org.

For peer review only

BMJ Open

National and subnational prevalence of adolescent pregnancy and changes in the associated sexual behaviours and sociodemographic determinants across three decades in Ghana, 1988 – 2019: analysis of national surveys

Journal:	*BMJ Open*
Manuscript ID	bmjopen-2022-068117.R2
Article Type:	Original research
Date Submitted by the Author:	27-Feb-2023
Complete List of Authors:	Mohammed, Shamsudeen; London School of Hygiene & Tropical Medicine, Department of Non-communicable Disease Epidemiology
**Primary Subject Heading**:	Public health
Secondary Subject Heading:	Epidemiology, Global health, Sexual health, Obstetrics and gynaecology
Keywords:	Epidemiology < INFECTIOUS DISEASES, Public health < INFECTIOUS DISEASES, REPRODUCTIVE MEDICINE

SCHOLARONE™
Manuscripts

I, the Submitting Author has the right to grant and does grant on behalf of all authors of the Work (as defined in the below author licence), an exclusive licence and/or a non-exclusive licence for contributions from authors who are: i) UK Crown employees; ii) where BMJ has agreed a CC-BY licence shall apply, and/or iii) in accordance with the terms applicable for US Federal Government officers or employees acting as part of their official duties; on a worldwide, perpetual, irrevocable, royalty-free basis to BMJ Publishing Group Ltd ("BMJ") its licensees and where the relevant Journal is co-owned by BMJ to the co-owners of the Journal, to publish the Work in this journal and any other BMJ products and to exploit all rights, as set out in our licence.

The Submitting Author accepts and understands that any supply made under these terms is made by BMJ to the Submitting Author unless you are acting as an employee on behalf of your employer or a postgraduate student of an affiliated institution which is paying any applicable article publishing charge ("APC") for Open Access articles. Where the Submitting Author wishes to make the Work available on an Open Access basis (and intends to pay the relevant APC), the terms of reuse of such Open Access shall be governed by a Creative Commons licence – details of these licences and which Creative Commons licence will apply to this Work are set out in our licence referred to above.

Other than as permitted in any relevant BMJ Author's Self Archiving Policies, I confirm this Work has not been accepted for publication elsewhere, is not being considered for publication elsewhere and does not duplicate material already published. I confirm all authors consent to publication of this Work and authorise the granting of this licence.

For peer review only

National and subnational prevalence of adolescent pregnancy and changes in the associated sexual behaviours and sociodemographic determinants across three decades in Ghana, 1988 – 2019: analysis of national surveys

Shamsudeen Mohammed [1]

[1]Department of Non-communicable Disease Epidemiology, Faculty of Epidemiology and Population Health, London School of Hygiene & Tropical Medicine, London, UK,

Correspondence: Shamsudeen Mohammed, Department of Non-communicable Disease Epidemiology, London School of Hygiene and Tropical Medicine, Keppel Street, London WC1E 7HT Shamsudeen.Mohammed1@lshtm.ac.uk

For peer review only

For peer review only - http://bmjopen.bmj.com/site/about/guidelines.xhtml

Abstract

Objective

Understanding the determinants of adolescent pregnancy and how they have changed over time is foundational to measuring progress and identifying approaches to improving adolescent reproductive health. This study examined changes over time in the prevalence and determinants of adolescent pregnancy in Ghana.

Methods

Eleven nationally representative surveys from the Ghana Demographic and Health Survey (1988, 1993, 1998, 2003, 2008, 2014), Multiple Indicator Cluster Survey (2006, 2011, 20117-18), and Malaria Indicator Survey (2016 and 2019) provided data on 14556 adolescent girls aged 15-19 for this analysis. A random-effect meta-analysis, time trends, and multivariable logistic regression models were used to track the prevalence and determinants of adolescent pregnancy.

Results

The prevalence of adolescent pregnancy in Ghana was 15.4% (95%CI=13.49%–17.30%). Rural areas (19.5%) had a higher prevalence of adolescent pregnancy than urban areas (10.6%). In the overall sample, middle adolescents (15-17 years) (aOR=0.30, 95%CI=0.23-0.39), adolescents in urban areas (aOR=0.56, 95%CI=0.43 - 0.74), large households (aOR=0.62, 95%CI=0.49-0.78), not working (aOR=0.62, 95%CI=0.43 - 0.90), and those unaware of contraceptive methods (aOR=0.49, 95%CI=0.27-0.90) were less likely to become pregnant. Adolescents from middle-income (aOR=0.91, 95%CI=0.67-1.24) or high-income (aOR=0.59, 95%CI=0.36-0.94) households, those who were semi-literate (aOR=0.56, 95%CI=0.39-0.82) or literate (aOR=0.28, 95%CI=0.21-0.37) and those with fewer previous sex partners were less likely to become pregnant. Not all determinants in the overall sample were consistently associated with adolescent pregnancy in the last three decades. Between 1988 and 1998, determinants of adolescent pregnancy were age, literacy, employment, household size, and whether the mother was alive. Between 2003 and 2008, age, literacy, household size, income, age of last sexual partner, number of previous partners, and contraception knowledge determined adolescent pregnancy. From 2011 to 2019, age, residence, literacy, and menstrual cycle knowledge were determinants of adolescent pregnancy.

Conclusion

Interventions and policies to prevent adolescent pregnancy should prioritise adolescents from disadvantaged backgrounds.

Strengths and limitations of this study

The study included eleven nationally representative surveys across three decades.

Because the sample size was large, the study had adequate power to detect and estimate more reliable results.

Adolescents from varied sociodemographic backgrounds were included in the study sample.

For peer review only - http://bmjopen.bmj.com/site/about/guidelines.xhtml

For peer review only

The secondary data sources lacked information on cultural and traditional practices that may influence adolescent pregnancy.

Background

Globally, one in five adolescent girls gives birth before age 18(1). In low- and middle-income countries (LMICs), about 21 million teenagers become pregnant each year(2), and if current trends continue, that number is expected to rise to 86 million by 2030 (1). The prevalence is highest in South Asia and sub-Saharan Africa(1). In 2018, a systematic review and meta-analysis of 52 studies revealed that the prevalence of adolescent pregnancy in sub-Saharan Africa is 19.3%, with substantial regional variation ranging from 16% in Central Africa to 22% in East Africa(3). The consequences of adolescent pregnancy can be devastating. For instance, teenagers who become pregnant face many negative social consequences, including isolation, stigma, rejection from peers and family, forced marriage, and even the risk of dropping out of school(4). Studies have shown that adolescents who become pregnant face an increased risk of unsafe abortion, eclampsia, obstruction during childbirth, obstetric fistula, preterm birth, and maternal death or disability(2,5–10).

Many epidemiological studies have shown a considerable disparity in the drivers of adolescent pregnancy, where disadvantaged adolescents are more at risk of pregnancy than those better off. For example, child marriage, non-school attendance, poverty, living in rural residence, household living arrangement, no maternal education, no paternal education, history of maternal teenage pregnancy, cost of contraceptives, misconceptions about contraceptives, and lack of parental counselling and guidance on sexual and reproductive health have been identified as the main sociodemographic and economic factors that drive adolescent pregnancy in Africa(3,11,12). Furthermore, sexual risk factors, including unwanted sexual advances from adult males, coercive sexual relations, unequal gender power relations, lack of comprehensive sexuality education, and early sexual debut, also increase adolescents susceptibility to pregnancy(3,10–12).

These findings from prior research demonstrate that adolescent pregnancy results from a complex interaction of social, economic, cultural, and personal factors. Understanding these different determinants can help identify the groups of adolescents most at risk of becoming pregnant. It can also inform interventions and policies to prevent adolescent pregnancy and improve the sexual and reproductive health of adolescent girls. Furthermore, tracking changes in the determinants over time can provide valuable information for assessing the efficacy of existing interventions and programs to reduce adolescent pregnancies.

In Ghana, not many studies have explored the predictors of adolescent pregnancy, despite the need for country-specific estimates and determinants to inform national policies and programmes on adolescent pregnancy and interventions targeted at improving the lives of adolescents. Moreover, the few studies conducted over the last few years had small samples and reported contradictory findings(13,14). The present study used data from eleven nationally representative surveys conducted between 1988 and 2019 to estimate the national and regional prevalence of adolescent pregnancy and track changes in the sociodemographic and sexual risk behaviours associated with adolescent pregnancy across three decades.

For peer review only - http://bmjopen.bmj.com/site/about/guidelines.xhtml

Methods

Eleven nationally representative cross-sectional surveys from the Ghana Demographic and Health Survey (1988, 1993, 1998, 2003, 2008, 2014), Multiple Indicator Cluster Survey (2006, 2011, 20117-18), and Malaria Indicator Survey (2016 and 2019) provided the data for this analysis. A detailed description of the methods used in the surveys, including information about survey design, sampling, and data collection, is published elsewhere(15). Briefly, the surveys used a multistage stratified cluster sampling method to select households from rural and urban clusters in Ghana's former ten administrative regions (now 16 regions). The participant questionnaires administered in these surveys are comparable, allowing data pooling across surveys and time. For all the surveys, trained field officers under the supervision of the Ghana Statistical Services administered the questionnaires face-to-face to women and men in selected households. The response rate in the surveys was higher than 90%. Data on only women aged 15 to 19 years were extracted from data files of the eleven surveys for the present analysis.

Variables

The outcome of interest was adolescent pregnancy. In this study, it was defined as women aged 15 to 19 who had previously given birth or were pregnant at the time of the survey. Across the surveys, the same questions were asked to elicit responses to measure the outcome. Women were asked, *"are you pregnant now?"* to determine those pregnant at the time of the survey and *"have you ever given birth?"* to ascertain those who had ever given birth. Response options for both questions were *"Yes"* or *"No"*. In order to reduce reverse causation and recall bias, the analysis of the factors associated with adolescent pregnancy was restricted to adolescents who were pregnant at the time of the survey. Adolescents sociodemographic and sexual risk behaviours were explored as potential explanatory factors of adolescent pregnancy. The sociodemographic factors included adolescent age, place of residence, sex of household head, number of household members *("≤4 members" and ">4 members"* (16)), mother still alive, father still alive, literacy level (*illiterate* = "cannot read at all", *semi-literate* = "able to read only partially sentence", and *literate* = "able to read whole sentence"), employment status, household income, and frequency of media exposure (newspaper + radio + TV). The sexual behaviours that were considered in the analysis are adolescents age at first sex *("≤ 14 years" vs "≥ 15 years"* (17)), number of previous sex partners, age of last sexual partner, knowledge of contraception, and knowledge of fertility period during menstruation. The explanatory variables were not available for all surveys.

Data management and statistical analysis

Data from each survey year were standardised and pooled into a single dataset before being grouped into three periods, 1988-1998, 2003-2008, and 2011-2019. The prevalence of adolescent pregnancy was estimated as a percentage of all adolescents aged 15 to 19 years for each survey year, the three eras, and the overall sample. Time trends of adolescent pregnancy and age patterns were plotted for survey years and selected sociodemographic factors. A random effect meta-analysis was used to pool the prevalence across the survey years to estimate the prevalence of adolescent pregnancy in Ghana. The pooled prevalence and corresponding 95% confidence intervals were presented using forest plots. Heterogeneity among the surveys was assessed using Higgins and Thompson's I^2 statistic(18). Subgroup analyses were performed to assess variations in the prevalence across the administrative regions of Ghana and rural-urban residence. To scale prevalence estimates to represent all adolescents in Ghana, denormalised survey weights were applied to correct for unequal sampling in certain areas and subgroups.

For peer review only - http://bmjopen.bmj.com/site/about/guidelines.xhtml

Bivariate logistic regression was used to investigate the unadjusted association between the explanatory variables and the binary outcome, adolescent pregnancy. Multivariable logistic regression was then used to determine the factors associated with adolescent pregnancy across the three decades (1988-1998, 2003-2008, and 2011-2019) and the overall sample (1988-2019) in an adjusted analysis. Potential determinants were categorised into distal (sociodemographic factors) and proximal (sexual behaviours) factors, and a conceptual framework was used to guide the analysis (Supplementary Figure 1). Based on the framework, distal factors were considered potential confounders of the association between the proximal factors and the outcome in the multivariable model. The logistic regression models accounted for stratification in the survey design, the year surveys were conducted, and calculated robust standard errors to account for clustering in the design of the surveys. To minimise the influence of reverse causation and recall bias, the logistic regression analysis was restricted to adolescents who were pregnant at the time of the survey. Because this study is exploratory rather than confirmatory, P<0.10 was used to determine statistically significant associations. A test for linear trend was performed for "literacy level", "household income", and "number of previous sex partners" using the Stata postestimation command, *contrast*. Estimates for explanatory variables not available in all the surveys were produced in a separate multivariable model. Stata version 17 was used for all data management and analysis.

Ethics approval

The Ghana Statistical Services and DHS program obtained ethical approval, participant consent, and permission for each primary survey. MEASURE DHS granted permission and access for the secondary use of the survey data for the present analysis. Because the data is available in the public domain with no personal identifiers, ethical approval was not sought for the secondary analysis.

Patient and public involvement

Patients or the public were not involved in the design, conduct, reporting, or dissemination plans of our research.

Results

Characteristics of the study sample

A nationally representative sample of 14556 adolescents aged 15 to 19 years from surveys conducted between 1988 and 2019 was included in the analysis. Table 1 presents the characteristics of the total study sample. Most adolescents lived in rural areas, in households with high income, headed by men, and with more than five household members. The majority of the sample were middle adolescents aged 15 to 17 years. Only a small percentage of the adolescents were orphans or worked at some point in the 12 months before the surveys. Nearly a third (28%) of the adolescents were illiterate, and 63.1% were exposed to mainstream media at least once a week (radio, television, or newspaper). Three-fourths (75.9%) of the adolescents initiated sex after age 14, and 66.7% had had only one sexual relationship. Most adolescents were aware of modern or traditional contraceptive methods, and for the majority of those who had had a sexual partner, their last sexual partner was an adult over age 20.

For peer review only - http://bmjopen.bmj.com/site/about/guidelines.xhtml

Table 1 Characteristics of the study population, 1988-2019 (n=14556)

	Unweighted number	Weighted percent
**Age**		
15 - 17	8986	62.4
18 - 19	5570	37.6
**Place of residence**		
Urban	6254	47.1
Rural	8302	52.9
**Sex of household head**		
Male	8718	59.9
Female	4989	40.1
**Number of household members**		
≤4 members	4209	30.8
>4 members	10347	69.2
**Mother still alive**		
Yes	5454	94.2
No	309	5.8
**Father still alive**		
Yes	4965	86.1
No	790	13.9
**Literacy level**		
Illiterate	3540	27.9
Semi-literate	1932	14.1
Literate	6238	58.1
**Employment**		
Yes	1985	30.3
No	4442	69.7
**Household income**		
Low income	6268	36.9
Middle income	2561	21.2
High income	4878	41.9
**Frequency of media exposure**		
Not at all	2483	22.1
Less than once a week	1282	14.8
At least once a week	5134	63.1
**Age at first sex**		
≤14 years	1107	24.1
≥15 years	3648	75.9
**Number of previous sex partners**		
1	764	66.7
2	243	23.8
Three or more	106	9.4

For peer review only

For peer review only - http://bmjopen.bmj.com/site/about/guidelines.xhtml

**Age of last sexual partner**		
Below 20 years	381	28.4
More than 20 years	993	71.6
**Knowledge of contraception**		
Knows no method	666	14.1
Knows modern or traditional method	4892	85.9
**knowledge of fertility period during menstruation**		
Just before or during menstrual period	546	14.6
Right after menstrual period or at any time	1964	52.1
Halfway between two menstrual periods	1067	33.3

Prevalence and time trends of adolescent pregnancy, 1988 - 2019

Based on a meta-analysis of the eleven national surveys, the prevalence of adolescent pregnancy was 15.4% (95%CI=13.49% – 17.30%) with significant variation across the surveys (I^2 = 89.9%, p < 0.001; Figure 1). There was a substantial regional disparity in the prevalence, ranging from 8.2% (95%CI=6.29%-10.39%) in the Greater Accra region to 19.3% (95% CI=15.75%-23.19%) in the Central region (Table 2; see Supplementary Figure 2 for details). Table 2 shows that the prevalence of adolescent pregnancy in rural areas (19.5%) was nearly twice that of urban areas (10.6%) (Table 2; see Supplementary Figure 3 for details).

The prevalence of adolescent pregnancy declined between 1988 and 1998, then remained relatively consistent from 2003 to 2008 before gradually increasing in the last decade (figure 2A). Adolescents from high-income households (Figures 2B) and those who were literate (Figures 2C) had a lower prevalence of adolescent pregnancy throughout the eleven surveys, with the steepest decline seen between 1993 and 2006. The surveys showed an upward trend of adolescent pregnancy with increasing age (figure 2D). There was no consistent pattern in the prevalence of adolescent pregnancy across ethnic groups.

Table 2 Subnational prevalence of adolescent pregnancy based on a meta-analysis of nationally representative surveys from 1988 to 2019 (see supplementary Figure 2 and supplementary Figure 3 for detailed forest plots for each region and urban-rural residence, respectively).

	Prevalence of adolescent pregnancy	95% confidence interval	I^2
**Region**			
Western region	14.42%	11.27% - 17.87%	66.5%
Central region	19.34%	15.75% - 23.19%	65.0%
Greater Accra region	8.23%	6.29% - 10.39%	62.6%
Volta region	16.18%	13.50% - 19.05%	36.8%
Eastern region	16.20%	13.93% - 18.61%	31.6%
Ashanti region	14.14%	11.94% - 16.48%	60.7%
Brong-Ahafo region	16.73%	12.66% - 21.22%	75.8%
Northern region	16.41%	11.62% - 21.82%	79.7%
Upper West region	11.48%	8.48% - 14.82%	0.0%
Upper East region	11.40%	8.73% - 14.36%	0.0%
**Residence**			
Urban	10.59%	8.69% - 12.66%	85.6%

For peer review only - http://bmjopen.bmj.com/site/about/guidelines.xhtml

Rural	19.50%	17.51% - 21.57%	79.3%

Note: I^2 measures the percentage of total variability that was due to between survey heterogeneity

Sexual behaviours and sociodemographic factors associated with adolescent pregnancy

Supplementary Table 1 summarises the sexual behaviour and sociodemographic characteristics according to the number and percentage of adolescents pregnant at the time of the survey. Results of unadjusted analysis of the association between sociodemographic factors, sexual behaviours and adolescent pregnancy are presented in Supplementary Table 2 and Supplementary Table 3, respectively.

In the overall sample, adolescent age, place of residence, household size, literacy level, household wealth, number of previous sex partners, and knowledge of contraception were the factors associated with adolescent pregnancy after adjusting for confounders (Table 3 and Table 4). Middle adolescents (age 15-17 years) were 70% less likely to become pregnant than late adolescents (aOR=0.30, 95%CI=0.23 - 0.39). Adolescents in urban areas were less likely to become pregnant than those in rural areas (aOR=0.56, 95%CI=0.43 - 0.74). The odds of adolescent pregnancy was lower among adolescents in large households (>4 household members) than among those in small households (aOR=0.62, 95%CI=0.49 - 0.78). There was a dose-response relationship between literacy level and adolescent pregnancy. Semi-literates (aOR=0.56, 95%CI=0.39 - 0.82) and literates (aOR=0.28, 95%CI=0.21 - 0.37) were less likely to become pregnant than illiterate. Adolescents who were not working had a reduced likelihood (aOR=0.62, 95%CI=0.43 - 0.90) of becoming pregnant than those who worked. There was a dose-response relationship between household income and adolescent pregnancy. Those from middle- (aOR=0.91, 95%CI=0.67 - 1.24) and high- (aOR=0.59, 95%CI=0.36 - 0.94) income households were less likely to become pregnant compared to those from low-income households. Similarly, a dose-response relationship was found between the number of previous sex partners and adolescent pregnancy. Adolescents with one (aOR=0.46, 0.23 - 0.95) or two (aOR=0.67, 95%CI=0.33 - 1.36) previous sex partners were less likely to become pregnant than those with three or more previous partners. Adolescents who were unaware of contraceptive methods were less likely (aOR=0.49, 95%CI=0.27 - 0.90) to become pregnant than those who knew about modern or traditional methods.

However, not all the determinants identified in the overall sample were consistently associated with adolescent pregnancy in the last three decades. Between 1988 and 1998, adolescent age, literacy level, employment status, household size, and whether the mother was alive were the only factors associated with adolescent pregnancy in the adjusted analysis (Table 3 and Table 4). Middle adolescents were less likely to become pregnant than late adolescents. Semi-literate or literate adolescents were less likely to become pregnant than illiterate. Those who had not worked in the previous 12 months were less likely than those who had worked to become pregnant. Adolescents in large households were less likely to become pregnant than those in small households. Adolescents whose mothers were still alive were less likely to experience pregnancy than those whose mothers had passed away.

Between 2003 and 2008, adolescent age, literacy level, household size, household wealth, age of last sexual partner, number of previous sex partners, and knowledge of contraception were the factors associated with adolescent pregnancy after adjusting for potential confounders (Table 3 and Table 4). Adolescents in middle and high-income families were less likely than those in low-income families to become pregnant. Middle adolescents were less likely to become pregnant than late adolescents.

For peer review only - http://bmjopen.bmj.com/site/about/guidelines.xhtml

Adolescent pregnancy was less likely among those in large households than those in small households. Semi-literates or literates were less likely to become pregnant than illiterate. Those whose last sexual partner was an older person were less likely to become pregnant than those whose last partner was an adolescent. Adolescent pregnancy was less likely among those who had one or two previous sex partners compared to those who had three or more previous partners. Those unaware of contraceptive methods had reduced odds of getting pregnant than those who knew about modern or traditional methods.

From 2011 to 2019, adolescent age, place of residence, literacy level, and knowledge of the fertility period during menstruation were the factors associated with adolescent pregnancy after adjusting for other sexual behaviours and sociodemographic factors (Table 3 and Table 4). Middle adolescents were less likely to become pregnant than late adolescents. Adolescents in urban areas were about 50% less likely than those in rural areas to become pregnant. Semi-literates and literates were less likely to become pregnant than illiterate. Adolescents who believed the most fertile period was just before or during menstruation or right after it were less likely to become pregnant than those who said it was halfway between two menstrual periods.

Table 3 Results of adjusted logistic regression analysis of the association between sociodemographic characteristics and adolescent pregnancy, 1988-2019

Survey years	1988 - 1998	2003 - 2008	2011 - 2019	Overall sample (1988-2019)
Population analysed (n)	2541	3350	8665	14556
Number currently pregnant	96	117	282	495
	**Adjusted odds ratio (95% CI)**	**Adjusted odds ratio (95% CI)**	**Adjusted odds ratio (95% CI)**	**Adjusted odds ratio (95% CI)**
**Age**	P < 0.001	P < 0.001	P < 0.001	P < 0.001
15 - 17	0.40 (0.24 - 0.66)	0.39 (0.24 - 0.64)	0.22 (0.15 - 0.33)	0.30 (0.23 - 0.39)
18 - 19	1.00	1.00	1.00	1.00
**Place of residence**	P = 0.23	P = 0.72	P = 0.002	P < 0.001
Urban	0.74 (0.45 - 1.22)	0.86 (0.40 - 1.89)	0.54 (0.36 - 0.81)	0.56 (0.43 - 0.74)
Rural	1.00	1.00	1.00	1.00
**Sex of household head**	P = 0.23	P = 0.06	P = 0.60	P = 0.42
Male	1.61 (0.73 - 3.54)	0.61 (0.36 - 1.02)	0.91 (0.63 - 1.31)	0.89 (0.66 - 1.19)
Female	1.00	1.00	1.00	1.00
**Number of household members**	P = 0.001	P = 0.02	P = 0.19	P < 0.001
≤4 members	1.00	1.00	1.00	1.00
>4 members	0.46 (0.29 - 0.73)	0.58 (0.37 - 0.91)	0.78 (0.54 - 1.13)	0.62 (0.49 - 0.78)
**Mother still alive**	P = 0.07		P = 0.48	P = 0.94
Yes	0.30 (0.08 - 1.09)	--	0.58 (0.13 - 2.58)	0.94 (0.24 - 3.77)
No	1.00	--	1.00	1.00
**Father still alive**	P = 0.85	P = 0.86	P = 0.99	P = 0.84
Yes	1.00	1.00	1.00	1.00

For peer review only - http://bmjopen.bmj.com/site/about/guidelines.xhtml

No	1.12 (0.36 - 3.44)	1.13 (0.30 - 4.21)	1.00 (0.40 - 2.51)	1.08 (0.52 - 2.24)
**Literacy level**	P = 0.001*	P < 0.001*	P < 0.001*	P < 0.001*
Illiterate	1.00	1.00	1.00	1.00
Semi-literate	0.55 (0.20 - 1.50)	0.42 (0.22 - 0.81)	0.66 (0.39 - 1.13)	0.56 (0.39 - 0.82)
Literate	0.40 (0.23 - 0.69)	0.27 (0.16 - 0.45)	0.27 (0.17 - 0.42)	0.28 (0.21 - 0.37)
**Employment**	P = 0.05	P = 0.29	P = 0.12	P = 0.01
Yes	1.00	1.00	1.00	1.00
No	0.61 (0.37 - 1.01)	0.73 (0.41 - 1.31)	0.59 (0.30 - 1.15)	0.62 (0.43 - 0.90)
**Household income**	P = 0.56	P < 0.001*	P = 0.27	P = 0.03*
Low income	1.00	1.00	1.00	1.00
Middle income	0.67 (0.32 - 1.39)	0.65 (0.39 - 1.09)	1.09 (0.70 - 1.69)	0.91 (0.67 - 1.24)
High income	0.88 (0.45 - 1.74)	0.30 (0.15 - 0.58)	0.63 (0.32 - 1.26)	0.59 (0.36 - 0.94)
**Frequency of media exposure**		P = 0.82	P = 0.23	P = 0.51
Not at all	--	1.00	1.00	1.00
Less than once a week	--	1.40 (0.45 - 4.37)	1.29 (0.62 - 2.69)	1.25 (0.68 - 2.32)
At least once a week	--	1.04 (0.49 - 2.20)	1.70 (0.91 - 3.18)	1.34 (0.82 - 2.20)

Table 4 Results of adjusted logistic regression analysis of the association between sexual behaviours and adolescent pregnancy, 1988-2019.

Survey years	1988 - 1998	2003 - 2008	2011 - 2019	Overall sample (1988-2019)
Population analysed (n)	2541	3350	8665	14556
Number currently pregnant	96	117	282	495
	**Adjusted odds ratio (95% CI)**	**Adjusted odds ratio (95% CI)**	**Adjusted odds ratio (95% CI)**	**Adjusted odds ratio (95% CI)**
**Age at first sex**	P = 0.63	P = 0.79	P = 0.10	P = 0.14
≤14 years	0.85 (0.45 - 1.63)	0.91 (0.44 - 1.88)	0.63 (0.36 - 1.10)	0.75 (0.52 - 1.10)
≥15 years	1.00	1.00	1.00	1.00
**Number of previous sex partners**		P = 0.03*	P = 0.79	P = 0.04*
1	--	0.29 (0.10 - 0.88)	0.73 (0.27 - 1.95)	0.46 (0.23 - 0.95)
2	--	0.56 (0.21 - 1.52)	0.90 (0.31 - 2.58)	0.67 (0.33 - 1.36)
Three or more	--	1.00	1.00	1.00
**Age of last sexual partner**		P = 0.02	P = 0.20	P = 0.39
Below 20 years	--	1.00	1.00	1.00
More than 20 years	--	0.14 (0.03 - 0.76)	2.00 (0.69 - 5.81)	1.46 (0.61 - 3.48)
**Knowledge of contraception**	P = 0.20	P = 0.08		P = 0.02
Knows no method	0.62 (0.30 - 1.29)	0.36 (0.11 - 1.15)	--	0.49 (0.27 - 0.90)

For peer review only - http://bmjopen.bmj.com/site/about/guidelines.xhtml

Knows modern or traditional method	1.00	1.00	--	1.00
**knowledge of fertility period during menstruation**	P = 0.56	P = 0.45	P = 0.02	P = 0.83
Just before or during menstrual period	2.09 (0.50 - 8.67)	1.88 (0.70 - 5.05)	0.17 (0.04 - 0.69)	0.90 (0.45 - 1.77)
Right after menstrual period or at any time	0.95 (0.42 - 2.13)	1.24 (0.60 - 2.56)	0.51 (0.24 - 1.07)	0.87 (0.56 - 1.36)
Halfway between two menstrual periods	1.00	1.00	1.00	1.00

Discussion

Based on a meta-analysis of eleven nationally representative surveys conducted between 1988 and 2019, the prevalence of adolescent pregnancy was 15.4%, with substantial regional variation. The prevalence was higher in rural areas than in urban areas. Over the last three decades, adolescent pregnancy peaked in 1988, followed by a progressive decline in the 1990s and early 2000s; but the prevalence has slowly increased over the last decade. The pooled data suggest that the prevalence of adolescent pregnancy rose with increasing adolescent age, a pattern consistent across all eleven surveys. The prevalence reported in this study is lower than the 18.8%, 19.3%, and 17.7% prevalence estimates for Africa, sub-Saharan Africa, and West Africa, respectively (3).

In line with the present findings, studies in Indonesia and Uganda reported a higher likelihood of pregnancy among teenagers living in rural areas, those with no or low education, and adolescents in low-income households(19,20). Similarly, in a systematic review of 24 studies in sub-Saharan Africa, about half of the included studies reported a higher likelihood of adolescent pregnancy in low-income households(11). Furthermore, an analysis of the 2014–2015 Rwanda Demographic and Health Survey confirmed that adolescents from large families were less likely than those from small families to get pregnant(21). Similar to the findings of this study, Jonas et al., found higher odds of adolescent pregnancy among those with more than one sex partner in South Africa(22). Though there was no association between adolescent age and pregnancy in the south African study, in a pooled analysis of data from five African countries and two cross-sectional studies in Ghana, older adolescents had higher odds of adolescent pregnancy (7,13,23). In support of the present findings, a qualitative study in Kenya found that adolescents engaged in incoming generating activities were more likely to get pregnant than those not working(24); however, a study in Ethiopia reported a contrary finding(25). In an earlier study in Ghana, there was no association between care status (orphanhood) and adolescent pregnancy(23), which agrees with the current results for 2011-2019 and the overall sample.

There are several reasons to explain the heightened vulnerability of adolescents in low-income households to pregnancy. In most cases, adolescents in these homes are not in school; instead, parents force them into early marriage to relieve the financial burden of taking care of the adolescents or prevent out-of-wedlock pregnancy(26). Several studies have revealed that most adolescent girls from poor homes engage in early sexual relationships for financial and material gains because parents cannot provide their basic needs, including food, toiletries, clothes, shoes, school supplies, fees, and housing (24,27–30). For instance, in a Ugandan study, adolescents acknowledged that they sometimes offer sex to boys/men as payment for money borrowed or in return for material items because their parents cannot provide(30). Under these situations, adolescents are less able to negotiate safe sex and condom use, increasing their

For peer review only - http://bmjopen.bmj.com/site/about/guidelines.xhtml

chance of pregnancy. Some may engage multiple sexual partners for greater financial benefits. Therefore, it is not surprising that in this study, adolescents with three or more sexual partners were more likely to experience pregnancy than those with one partner.

Additionally, some adolescents engage in all manner of income-generating activities to meet their basic needs, and this exposes them to mistreatment, sexual abuse, and exploitation, particularly those working as domestic help, bartenders, waitresses, shopkeepers, and selling at night or work that involves late-night shifts(24,30). This is probably why adolescents who worked 12 months prior to the surveys analysed in this study had a higher chance of becoming pregnant. Also, it has been suggested that some adolescents spend the income from these jobs on activities that increase their risk of pregnancy, such as alcohol, drugs, and nightclubs (24). In large families, adolescents are supervised, guided, and supported emotionally and financially not only by their parents but also by other family members to make sexual and reproductive health decisions favouring pregnancy prevention, reducing the likelihood of adolescent pregnancy in such families, as reported in this study. Also, in these families' adolescents who are uncomfortable discussing sexual health issues with their parents have other family members to consult.

The lower likelihood of pregnancy among adolescents in urban areas may be due to the advantages the urban setting offers. Urban residents have easy access to pregnancy prevention information and services, including easy access to various effective modern contraceptive methods (including emergency contraceptives) through the many hospitals, clinics, pharmacy shops, and family planning centres in urban areas. In rural areas, misconceptions are promoted, even when contraceptives are available, and sexually active adolescents usually rely on traditional contraceptive methods that are ineffective. Furthermore, traditional and cultural practices that promote early adolescent marriage are rarely practised in most urban settings, reducing adolescents' exposure to pregnancy. In addition, most adolescents in urban areas are better educated than their rural counterparts, increasing their ability to understand and effectively use sexual and reproductive health information to reduce their susceptibility to pregnancy. This further explains why adolescents who were semi-literate or literate in this study were less likely to experience pregnancy than those who were illiterate. Also, schooling delays marriage and empowers girls to make rational decisions in their relationships that prevent pregnancy(10).

Most adolescents in the present study did not know the fertile phase of the menstrual cycle. Therefore, it is surprising that those who did not know the fertile period were less likely to become pregnant. It is possible that these girls had not initiated sex or that those who correctly identified the fertile phase learned it when they became pregnant (reverse causality). This may also explain the unexpected finding on knowledge about contraceptives. However, it was not surprising that older adolescents were more likely to get pregnant than younger adolescents because most older adolescents are sexually active and engage in more sexual risk behaviours than middle adolescents. It is also possible that parents and other adults support and guide middle-aged adolescents more than they do older teenagers. In addition, the lower adolescent pregnancy among those whose last partner was older in this study is possible due to better pregnancy prevention and sexual and reproductive health knowledge of older partners.

Age-appropriate sexual and reproductive health education as part of the school curriculum and in communities is essential to help adolescents from all settings, income groups, and family backgrounds make rational decisions that will delay pregnancy and motherhood. Adolescents access and use of contraceptives, including emergency contraceptives, should be promoted, particularly in rural communities and low-income households. Traditional leaders and stakeholders who promote cultural and

For peer review only - http://bmjopen.bmj.com/site/about/guidelines.xhtml

traditional practices that encourage early motherhood should be educated on the harmful effects of adolescent childbearing, and such practices discouraged. Girls should be encouraged and motivated to stay in school even when they become pregnant, considering the long-term repercussions of dropping out. In addition, interventions to reduce adolescent pregnancy should include boys and men.

The main strength of this study is the use of a large nationally representative sample that captures adolescents from various sociodemographic backgrounds and settings. The study's findings are generalisable to all adolescents in Ghana and more relevant to national policies than previous studies that mainly focused on individual communities. One major limitation of the study is the possibility of reverse causality and recall bias. To minimise the effect of reverse causality, analysis of the determinants was restricted to currently pregnant adolescents. However, it is important to note that this study was not designed for causal relationships, so I make no such claims. Furthermore, it is possible that the prevalence reported in this study was underestimated because most Ghanaian women admit to pregnancy after the first trimester. In addition, the definition of adolescent pregnancy in this study excluded abortion. Furthermore, because the study relied on secondary data, cultural and traditional practices that could potentially impact adolescent pregnancy were not considered in the analysis because data were unavailable on these practices. Future studies should address the limitations with longitudinal and qualitative studies.

Conclusion

Adolescent pregnancy has risen slowly over the last decade, following a steady prevalence between 1998 and 2008. Regional and urban-rural disparities in the prevalence were substantial, and age patterns showed that adolescent pregnancy increased as adolescents got older. The factors influencing adolescent pregnancy differed across the eras except for literacy level and adolescent age; illiteracy and older adolescent age were associated with a higher likelihood of adolescent pregnancy across the three decades from 1988 to 2019.

Ethics approval and consent to participate: The Ghana Statistical Services and DHS program obtained ethical approval, participant consent, and permission for each primary survey. MEASURE DHS granted permission and access for the secondary use of the survey data for the present analysis. Because the data is available in the public domain with no personal identifiers, ethical approval was not sought for the secondary analysis.

Consent for publication: Not applicable

Data sharing statement: All the datasets analysed are publicly available with the measure DHS program (https://dhsprogram.com) and UNICEF's MICS programme (https://mics.unicef.org/).

Competing interests: None

Funding: This study was supported by the Economic and Social Research Council (ESRC) [grant number: ES/P000592/1]. The ESRC had no role in the study's design, analysis of the data, or manuscript preparation.

Authors' contributions: SM conceived and designed the study, accessed the data, conducted the literature search, performed the statistical analysis, and drafted the manuscript.

For peer review only - http://bmjopen.bmj.com/site/about/guidelines.xhtml

Acknowledgements: I would like to thank measure DHS and UNICEF's MICS programme for prompt approval and access to the datasets for the analysis.

References

1. UNFPA. ADOLESCENT PREGNANCY : A Review of the Evidence [Internet]. New York, USA; 2013. Available from: https://www.unfpa.org/adolescent-pregnancy

2. World Health Organization. Adolescent pregnancy [Internet]. 2022 [cited 2023 Feb 20]. Available from: https://www.who.int/en/news-room/fact-sheets/detail/adolescent-pregnancy

3. Kassa GM, Arowojolu AO, Odukogbe AA, Yalew AW. Prevalence and determinants of adolescent pregnancy in Africa: A systematic review and Meta-analysis 11 Medical and Health Sciences 1117 Public Health and Health Services. Reprod Health. 2018;15(1):1–17.

4. Unicef. Early Childbearing [Internet]. 2021 [cited 2022 Sep 6]. Available from: https://data.unicef.org/topic/child-health/adolescent-health/

5. UNFPA. Girlhood, not motherhood: Preventing adolescent pregnancy [Internet]. United Nations Population Fund. New York, USA; 2015. Available from: https://www.unfpa.org/sites/default/files/pub-pdf/Girlhood_not_motherhood_final_web.pdf

6. Yussif AS, Lassey A, Ganyaglo GYK, Kantelhardt EJ, Kielstein H. The long-term effects of adolescent pregnancies in a community in Northern Ghana on subsequent pregnancies and births of the young mothers. Reprod Health. 2017;14(1):1–7.

7. Tetteh J, Nuertey BD, Dwomoh D, Udofia EA, Mohammed S, Adjei-Mensah E, et al. Teenage pregnancy and experience of physical violence among women aged 15-19 years in five African countries: Analysis of complex survey data. PLoS One [Internet]. 2020;15(10 October):1–18. Available from: http://dx.doi.org/10.1371/journal.pone.0241348

8. Dzotsi HT, Oppong Asante K, Osafo J. Challenges associated with teenage motherhood in Ghana: a qualitative study. Vulnerable Child Youth Stud [Internet]. 2020;15(1):85–96. Available from: https://doi.org/10.1080/17450128.2020.1719251

9. Gbogbo S. Early motherhood: voices from female adolescents in the Hohoe Municipality, Ghana—a qualitative study utilizing Schlossberg's Transition Theory. Int J Qual Stud Health Well-being [Internet]. 2020;15(1). Available from: https://doi.org/10.1080/17482631.2020.1716620

10. UNFPA. Adolescent pregnancy: A matter of human rights [Internet]. 2017 [cited 2022 Jan 2]. Available from: https://www.unfpa.org/adolescent-pregnancy#readmore-expand

11. Yakubu I, Salisu WJ. Determinants of adolescent pregnancy in sub-Saharan Africa: A systematic review. Reprod Health. 2018;15(1).

12. Gunawardena N, Fantaye AW, Yaya S. Predictors of pregnancy among young people in sub-Saharan Africa: A systematic review and narrative synthesis. BMJ Glob Heal. 2019;4(3):1–8.

For peer review only - http://bmjopen.bmj.com/site/about/guidelines.xhtml

13. Asare BYA, Baafi D, Dwumfour-Asare B, Adam AR. Factors associated with adolescent pregnancy in the Sunyani Municipality of Ghana. Int J Africa Nurs Sci. 2019;10(February):87–91.

14. Bedzo JY, Manortey S. Factors Influencing Teenage Pregnancy in the Lower Manya Krobo Municipality in the Eastern Region of Ghana: A Cross-Sectional Study. OALib. 2019;06(12):1–17.

15. The DHS Program. Publications Catalog [Internet]. 2022 [cited 2022 Feb 4]. Available from: https://dhsprogram.com/Publications/Publication-Search.cfm?ctry_id=14&country=Ghana

16. United Nations Population Division. Population Fact Sheet No. 2: Household size and composition around the world [Internet]. 2017. Available from: http://www.un.org/en/development/desa/population/publications/pdf/popfacts/PopFacts_2017-2.pdf

17. Amoateng AY, Ewemooje OS, Biney E. Prevalence and determinants of adolescent pregnancy among women of reproductive age in South Africa. Afr J Reprod Health. 2022;26(1):82–91.

18. Higgins JPT, Thompson SG. Quantifying heterogeneity in a meta-analysis. Stat Med. 2002;21(11):1539–58.

19. Gayatri M, Gayatri M, Permata J, Kusuma HP, Timur J. Socioeconomic determinants of adolescent pregnancy in Indonesia : A population-based survey Key Messages : Ann Trop Med Public Heal. 2021;24(01).

20. Byonanebye J, Brazauskas R, Tumwesigye N, Young S, May T, Cassidy L. Geographic variation and risk factors for teenage pregnancy in Uganda. Afr Health Sci. 2020;20(4):1898–907.

21. Uwizeye D, Muhayiteto R, Kantarama E, Wiehler S, Murangwa Y. Prevalence of teenage pregnancy and the associated contextual correlates in Rwanda. Heliyon [Internet]. 2020;6(10):e05037. Available from: https://doi.org/10.1016/j.heliyon.2020.e05037

22. Jonas K, Crutzen R, Van Den Borne B, Sewpaul R, Reddy P. Teenage pregnancy rates and associations with other health risk behaviours: A three-wave cross-sectional study among South African school-going adolescents. Reprod Health [Internet]. 2016;13(1). Available from: http://dx.doi.org/10.1186/s12978-016-0170-8

23. Morhe ESK, Tagbor HK, Ankobea FK, Danso KA. Reproductive experiences of teenagers in the Ejisu-Juabeng district of Ghana. Int J Gynecol Obstet [Internet]. 2012;118(2):137–40. Available from: http://dx.doi.org/10.1016/j.ijgo.2012.03.035

24. Juma M, Alaii J, Bartholomew LK, Askew I, Van Den Born B. Understanding orphan and non-orphan adolescents' sexual risks in the context of poverty: A qualitative study in Nyanza Province, Kenya. BMC Int Health Hum Rights. 2013;13(32).

25. Alemayehu T, Haider J, Habte D. Determinants of adolescent fertility in Ethiopia [Internet]. Vol. 24, The Ethiopian Journal of Health Development. 2010. p. 30–8. Available from: http://search.ebscohost.com/login.aspx?direct=true&AuthType=cookie,ip,shib&db=awn&AN=ejhd-62942&site=ehost-live

26. UNICEF. Early marriage: child spouses. [Internet]. Innocenti Digest. Florence, Italy Piazza; 2001. Available from: https://www.unicef-irc.org/publications/pdf/digest7e.pdf

27. McCleary-Sills J, Douglas Z, Rwehumbiza A, Hamisi A, Mabala R. Gendered norms, sexual

For peer review only - http://bmjopen.bmj.com/site/about/guidelines.xhtml

exploitation and adolescent pregnancy in rural Tanzania. Reprod Health Matters [Internet]. 2013;21(41):97–105. Available from: http://dx.doi.org/10.1016/S0968-8080(13)41682-8

28. Krugu JK, Mevissen FEF, Prinsen A, Ruiter RAC. Who's that girl? A qualitative analysis of adolescent girls' views on factors associated with teenage pregnancies in Bolgatanga, Ghana. Reprod Health [Internet]. 2016;13(1):1–12. Available from: http://dx.doi.org/10.1186/s12978-016-0161-9

29. Zakayo MO, Lwelamira J. Sexual Behaviours among Adolescents in Community Secondary Schools in Rural Areas of Central Tanzania: A Case of Bahi District in Dodoma Region. Curr Res J Soc Sci. 2011;3(4):374–80.

30. Nabugoomu J, Seruwagi GK, Hanning R. What can be done to reduce the prevalence of teen pregnancy in rural Eastern Uganda?: Multi-stakeholder perceptions. Reprod Health. 2020;17(134):1–12.

Figure Legend/Caption

Figure 1: National prevalence of adolescent pregnancy, 1988 - 2019

Figure 2: Time trends of adolescent pregnancy by (A) survey year (B) household income (C) literacy level (D) adolescent age (E) residence (F) ethnic group

For peer review only

For peer review only

For peer review only - http://bmjopen.bmj.com/site/about/guidelines.xhtml

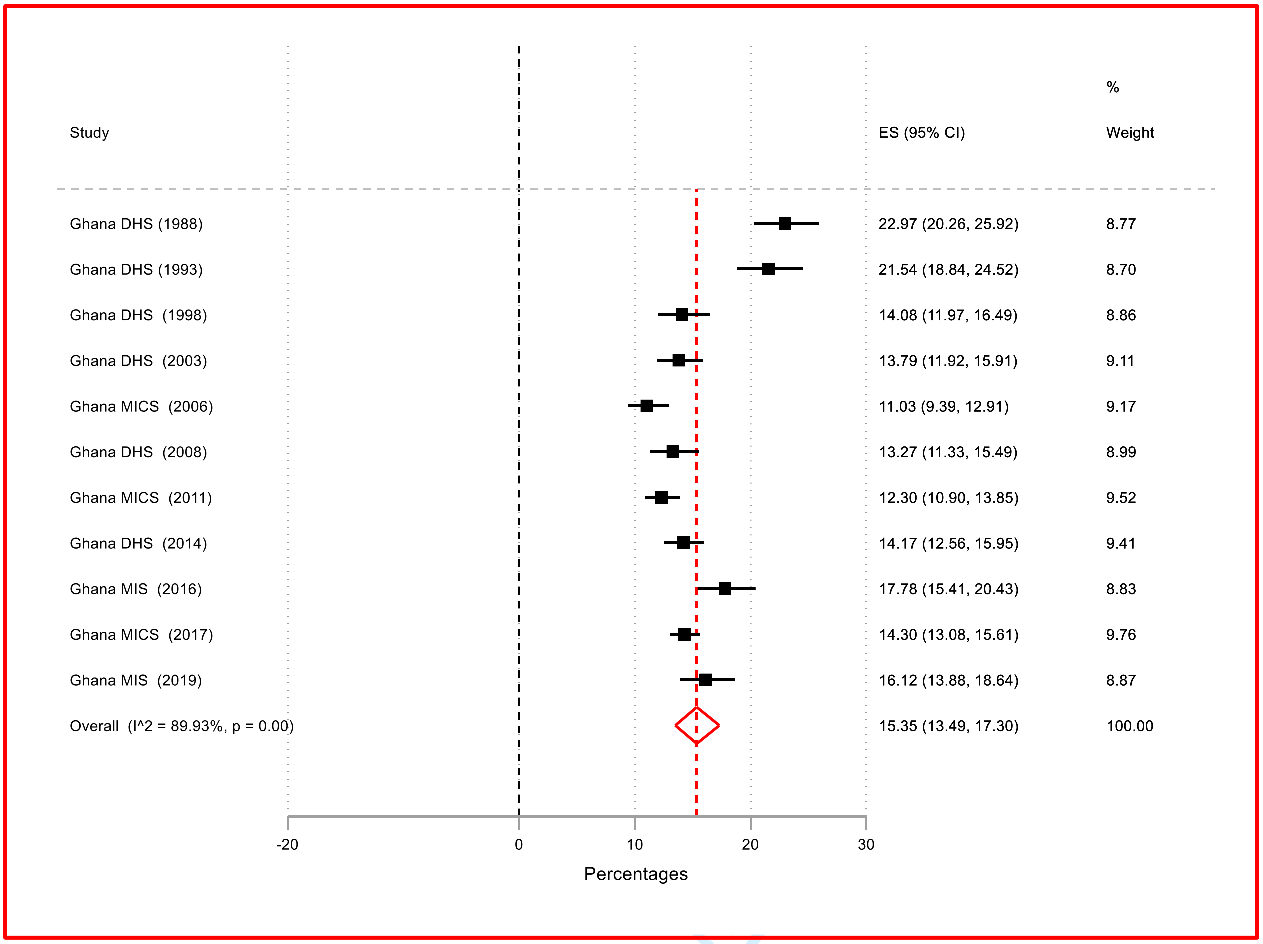

Figure 1: National prevalence of adolescent pregnancy, 1988 - 2019

For peer review only - http://bmjopen.bmj.com/site/about/guidelines.xhtml

For peer review only

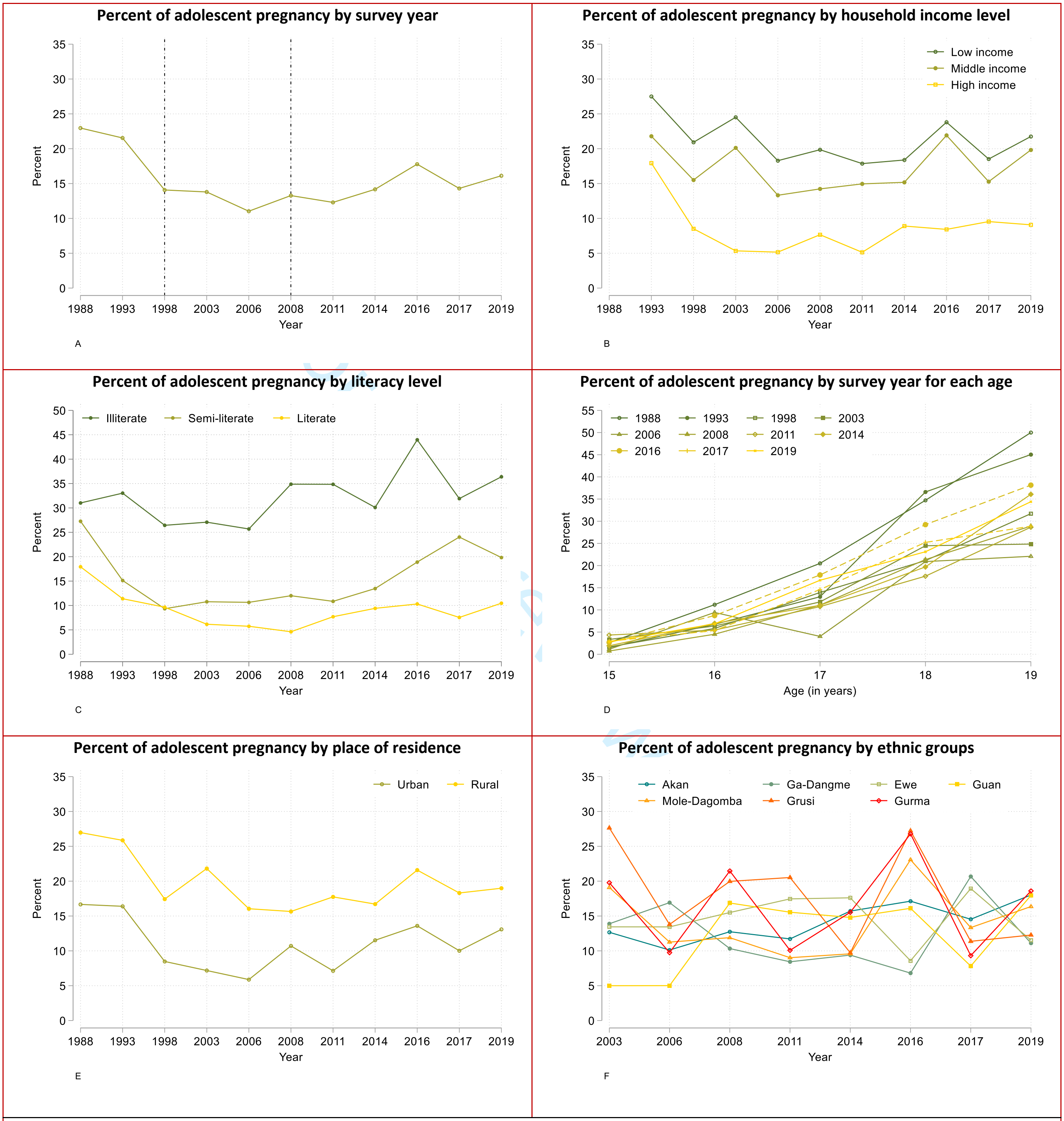

Figure 2: Time trends of adolescent pregnancy by (A) survey year (B) household income (C) literacy level (D) adolescent age (E) residence (F) ethnic group

Supplementary Figure 1. Conceptual model of factors associated with adolescent pregnancy

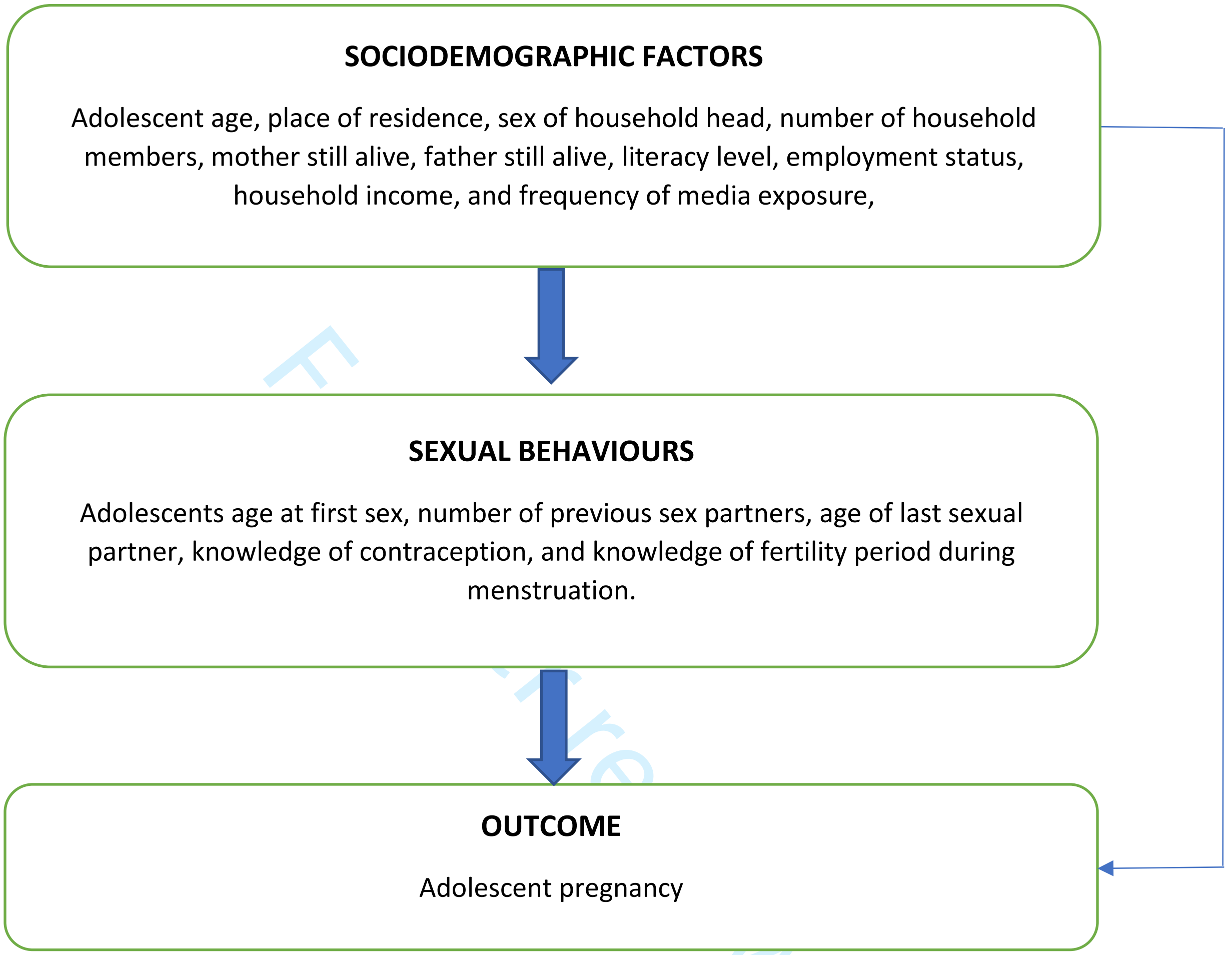

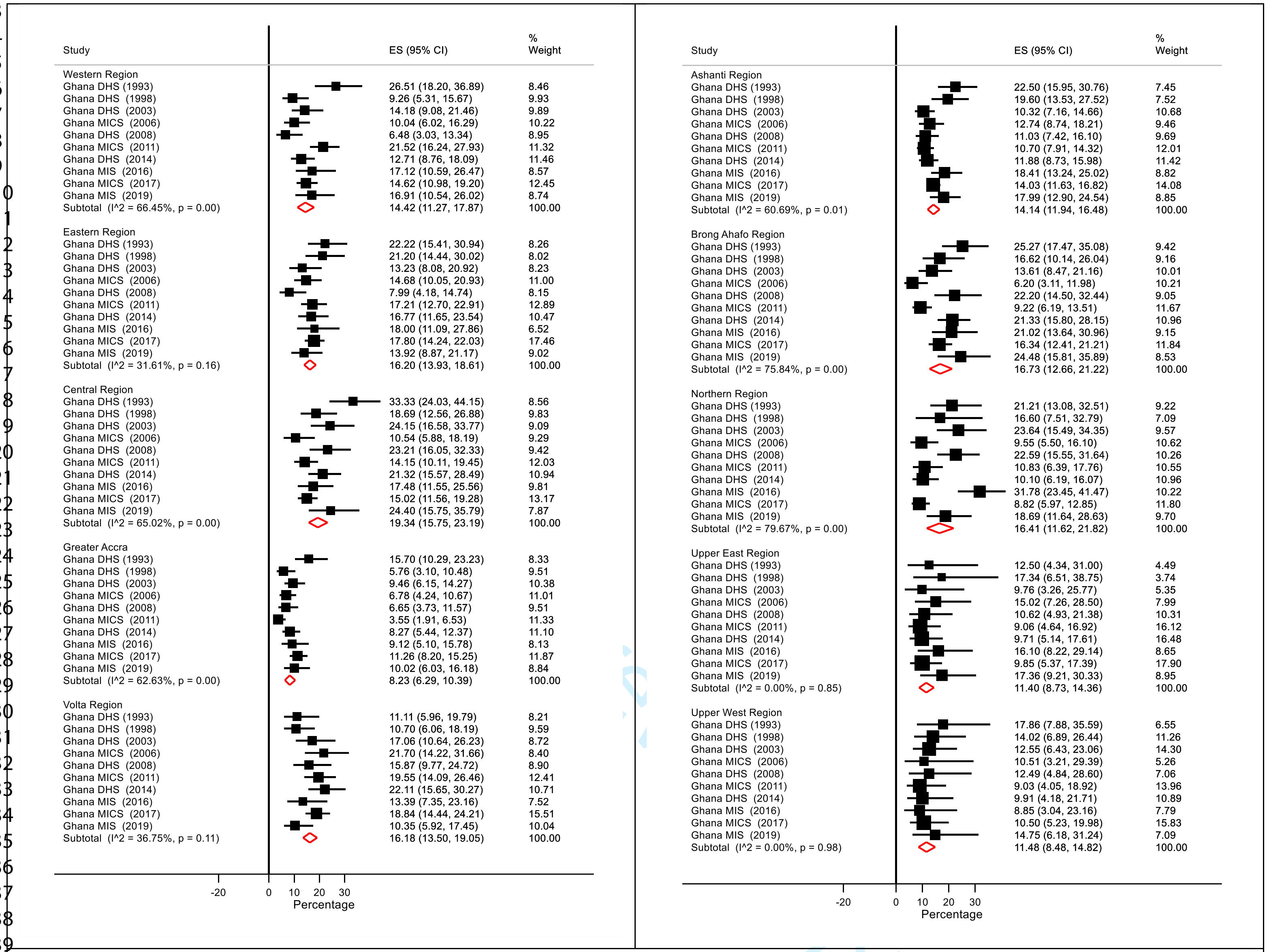

Supplementary Figure 2: Regional prevalence of adolescent pregnancy, 1993 - 2019

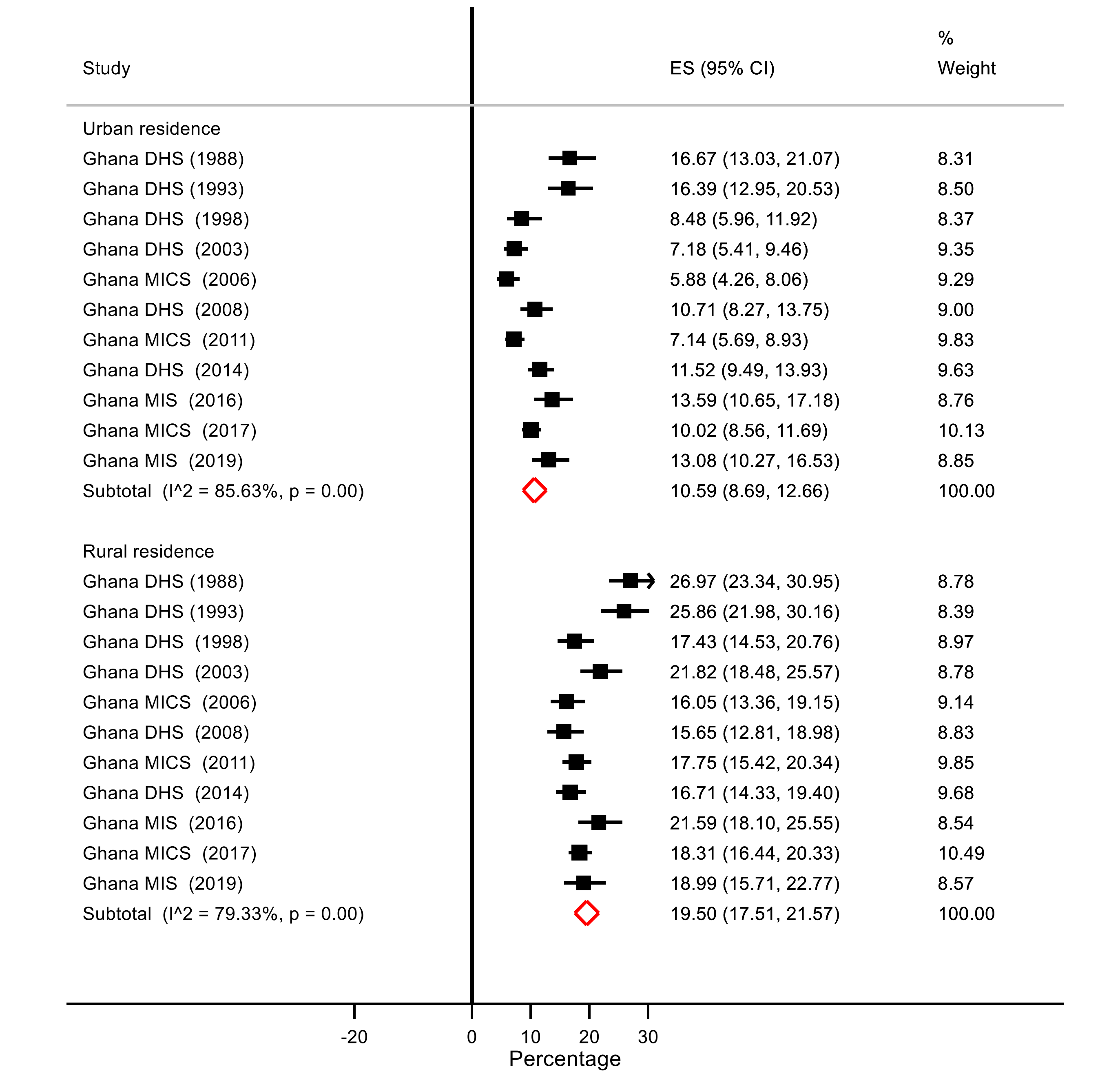

Supplementary Figure 3: Urban-rural prevalence of adolescent pregnancy , 1988 - 2019

Supplementary Table 1 Characteristics of the study population according to number and percent currently pregnant, 1988 to 2019.

Survey years	1988 - 1998		2003 - 2008		2011 - 2019	
Population analysed (unweighted)	2541		8665		8383	
Number (unweighted) currently pregnant	96		117		282	
Percent (weighted) currently pregnant	3.4%		3.7%		3.7%	
Mean age (SD), years	16.9 (0.03)		16.9 (0.03)		16.9 (0.03)	
	n	%	n	%	n	%
**Age**						
15 - 17	27	1.9	40	2.2	87	1.8
18 - 19	69	5.6	77	6.2	195	7.1
**Place of residence**						
Urban	27	2.6	30	2.2	85	2.6
Rural	69	3.9	87	5.3	197	4.8
**Sex of household head**						
Male	35	3.0	66	3.2	191	3.9
Female	26	2.6	51	4.6	91	3.4
**Number of household members**						
≤4 members	54	5.5	61	5.7	102	4.8
>4 members	42	2.5	56	2.8	180	3.2
**Mother still alive**						
Yes	25	12.9	23	2.2	59	1.8
No	4	33.3	0	0.0	3	1.7
**Father still alive**						
Yes	24	14.0	18	2.0	49	1.7
No	5	15.2	4	2.4	13	2.8
**Literacy level**						
Illiterate	62	6.1	81	8.6	119	8.7
Semi-literate	7	2.8	14	3.2	38	4.7
Literate	27	2.0	21	1.7	68	2.1
**Employment**						
Yes	48	5.9	49	7.3	27	5.9
No	48	2.4	37	2.7	33	2.8
**Household income**						
Low income	26	3.9	71	6.6	178	5.1
Middle income	13	2.2	28	4.4	56	3.8
High income	22	2.3	18	1.5	48	2.2
**Frequency of media exposure**						
Not at all	-	-	15	7.0	70	3.6
Less than once a week	-	-	11	7.7	22	2.6
At least once a week	-	-	58	3.4	114	3.6
**Age at first sex**						
≤14 years	19	6.6	17	9.7	48	7.7

For peer review only

≥15 years	64	7.2	81	9.4	140	8.6
**Number of previous sex partners**						
1	-	-	23	8.2	36	8.2
2	-	-	11	14.8	16	9.8
Three or more	-	-	6	24.0	8	11.3
**Age of last sexual partner**						
Below 20 years	-	-	5	9.3	14	4.7
More than 20 years	-	-	2	1.1	54	7.2
**Knowledge of contraception**						
Knows no method	14	2.7	4	2.5	-	-
Knows modern or traditional method	55	4.9	82	4.2	-	-
**knowledge of fertility period during menstruation**						
Just before or during menstrual period	4	8.1	12	5.4	4	1.3
Right after menstrual period or at any time	18	5.9	42	5.1	27	3.9
Halfway between two menstrual periods	13	6.1	12	3	23	6.8

For peer review only

Supplementary Table 2 Results of unadjusted logistic regression analysis of the association between sociodemographic characteristics and adolescent pregnancy, 1988-2019

Survey years	1988 - 1998	2003 - 2008	2011 - 2019	Overall sample (1988-2019)
Population analysed (n)	2541	3350	8665	14556
Number currently pregnant	96	117	282	495
	**Unadjusted odds ratio (95% CI)**	**Unadjusted odds ratio (95% CI)**	**Unadjusted odds ratio (95% CI)**	**Unadjusted odds ratio (95% CI)**
**Age**	P < 0.001	P < 0.001	P < 0.001	P < 0.001
15 - 17	1.00	1.00	1.00	1.00
18 - 19	3.08 (1.87 - 5.08)	2.95 (1.86 - 4.68)	4.23 (2.98 - 6.00)	3.62 (2.83 - 4.64)
**Place of residence**	P = 0.08	P < 0.001	P < 0.001	P < 0.001
Urban	0.64 (0.39 - 1.06)	0.40 (0.24 - 0.66)	0.53 (0.38 - 0.74)	0.52 (0.41 - 0.66)
Rural	1.00	1.00	1.00	1.00
**Sex of household head**	P = 0.68	P = 0.09	P = 0.37	P = 0.89
Male	1.00	1.00	1.00	1.00
Female	0.87 (0.46 - 1.67)	1.45 (0.94 - 2.25)	0.86 (0.61 - 1.20)	0.98 (0.76 - 1.27)
**Number of household members**	P < 0.001	P < 0.001	P = 0.01	P < 0.001
≤4 members	1.00	1.00	1.00	1.00
>4 members	0.43 (0.28 - 0.67)	0.48 (0.32 - 0.71)	0.65 (0.47 - 0.90)	0.56 (0.45 - 0.69)
**Mother still alive**	P = 0.07		P = 0.95	P = 0.53
Yes	0.30 (0.08 - 1.10)	--	1.04 (0.25 - 4.37)	0.76 (0.33 - 1.77)
No	1.00	--	1.00	1.00
**Father still alive**	P = 0.86	P = 0.74	P = 0.15	P = 0.16
Yes	1.00	1.00	1.00	1.00
No	1.10 (0.37 - 3.24)	1.23 (0.36 - 4.19)	1.69 (0.83 - 3.41)	1.47 (0.87 - 2.48)
**Literacy level**	P < 0.001	P < 0.001	P < 0.001	P < 0.001
Illiterate	1.00	1.00	1.00	1.00
Semi-literate	0.44 (0.18 - 1.07)	0.36 (0.19 - 0.68)	0.52 (0.30 - 0.89)	0.48 (0.33 - 0.70)
Literate	0.31 (0.19 - 0.51)	0.18 (0.11 - 0.29)	0.22 (0.15 - 0.34)	0.24 (0.18 - 0.31)
**Employment**	P < 0.001	P < 0.001	P = 0.02	P < 0.001
Yes	1.00	1.00	1.00	1.00
No	0.40 (0.25 - 0.62)	0.35 (0.21 - 0.58)	0.46 (0.24 - 0.88)	0.39 (0.29 - 0.52)
**Household income**	P = 0.13	P < 0.001	P < 0.001	P < 0.001
Low income	1.00	1.00	1.00	1.00
Middle income	0.56 (0.27 - 1.15)	0.65 (0.41 - 1.03)	0.74 (0.50 - 1.09)	0.70 (0.53 - 0.93)
High income	0.57 (0.31 - 1.05)	0.22 (0.13 - 0.38)	0.42 (0.27 - 0.63)	0.37 (0.27 - 0.50)

For peer review only

**Frequency of media exposure**		P = 0.02	P = 0.50	P = 0.73
Not at all	--	2.10 (1.10 - 4.01)	0.99 (0.65 - 1.52)	1.13 (0.79 - 1.61)
Less than once a week	--	2.33 (0.94 - 5.74)	0.72 (0.41 - 1.26)	0.93 (0.58 - 1.49)
At least once a week	--	1.00	1.00	1.00

For peer review only

Supplementary Table 3 Results of unadjusted logistic regression analysis of the association between sexual behaviours and adolescent pregnancy, 1988-2019.

Survey years	1988 - 1998	2003 - 2008	2011 - 2019	Overall sample (1988-2019)
Population analysed (n)	2541	3350	8665	14556
Number currently pregnant	96	117	282	495
	**Unadjusted odds ratio (95% CI)**	**Unadjusted odds ratio (95% CI)**	**Unadjusted odds ratio (95% CI)**	**Unadjusted odds ratio (95% CI)**
**Age at first sex**	P = 0.79	P = 0.0.91	P = 0.56	P = 0.61
≤14 years	0.92 (0.49 - 1.71)	1.04 (0.53 - 2.03)	0.88 (0.57 - 1.36)	0.92 (0.68 - 1.26)
≥15 years	1.00	1.00	1.00	1.00
**Number of previous sex partners**		P = 0.06	P = 0.72	P = 0.08
1	-	0.28 (0.10 - 0.83)	0.70 (0.28 - 1.75)	0.46 (0.23 - 0.93)
2	-	0.55 (0.20 - 1.53)	0.85 (0.31 - 2.31)	0.69 (0.34 - 1.41)
Three or more	-	1.00	1.00	1.00
**Age of last sexual partner**		P = 0.02	P = 0.26	P = 0.53
Below 20 years	-	1.00	1.00	1.00
More than 20 years	-	0.11 (0.02 - 0.65)	1.56 (0.72 - 3.37)	1.24 (0.63 - 2.45)
**Knowledge of contraception**	P = 0.09	P = 0.34		P = 0.06
Knows no method	0.55 (0.27 - 1.10)	0.57 (0.18 - 1.79)	-	0.57 (0.32 - 1.03)
Knows modern or traditional method	1.00	1.00	-	1.00
**knowledge of fertility period during menstruation**	P = 0.88	P = 0.23		P = 0.78
Just before or during menstrual period	1.35 (0.36 - 5.13)	1.85 (0.74 - 4.61)	-	0.81 (0.44 - 1.52)
Right after menstrual period or at any time	0.97 (0.44 - 2.16)	1.75 (0.90 - 3.39)	-	1.00 (0.67 - 1.49)
Halfway between two menstrual periods	1.00	1.00	-	1.00

For peer review only

STROBE Statement—Checklist of items that should be included in reports of ***cross-sectional studies***

	Item No	Recommendation	Page No
**Title and abstract**	1	(*a*) Indicate the study's design with a commonly used term in the title or the abstract	1
		(*b*) Provide in the abstract an informative and balanced summary of what was done and what was found	2
**Introduction**			
Background/rationale	2	Explain the scientific background and rationale for the investigation being reported	3
Objectives	3	State specific objectives, including any prespecified hypotheses	3
**Methods**			
Study design	4	Present key elements of study design early in the paper	3
Setting	5	Describe the setting, locations, and relevant dates, including periods of recruitment, exposure, follow-up, and data collection	4
Participants	6	(*a*) Give the eligibility criteria, and the sources and methods of selection of participants	4
Variables	7	Clearly define all outcomes, exposures, predictors, potential confounders, and effect modifiers. Give diagnostic criteria, if applicable	4
Data sources/ measurement	8*	For each variable of interest, give sources of data and details of methods of assessment (measurement). Describe comparability of assessment methods if there is more than one group	*4*
Bias	9	Describe any efforts to address potential sources of bias	4
Study size	10	Explain how the study size was arrived at	4
Quantitative variables	11	Explain how quantitative variables were handled in the analyses. If applicable, describe which groupings were chosen and why	4
Statistical methods	12	(*a*) Describe all statistical methods, including those used to control for confounding	4
		(*b*) Describe any methods used to examine subgroups and interactions	4
		(*c*) Explain how missing data were addressed	4
		(*d*) If applicable, describe analytical methods taking account of sampling strategy	4
		(*e*) Describe any sensitivity analyses	NA
**Results**			
Participants	13*	(a) Report numbers of individuals at each stage of study—eg numbers potentially eligible, examined for eligibility, confirmed eligible, included in the study, completing follow-up, and analysed	5
		(b) Give reasons for non-participation at each stage	
		(c) Consider use of a flow diagram	
Descriptive data	14*	(a) Give characteristics of study participants (eg demographic, clinical, social) and information on exposures and potential confounders	5
		(b) Indicate number of participants with missing data for each variable of interest	
Outcome data	15*	Report numbers of outcome events or summary measures	5
Main results	16	(*a*) Give unadjusted estimates and, if applicable, confounder-adjusted estimates and their precision (eg, 95% confidence interval). Make clear which confounders were adjusted for and why they were included	5-6

For peer review only - http://bmjopen.bmj.com/site/about/guidelines.xhtml

		(*b*) Report category boundaries when continuous variables were categorized	NA
		(*c*) If relevant, consider translating estimates of relative risk into absolute risk for a meaningful time period	
Other analyses	17	Report other analyses done—eg analyses of subgroups and interactions, and sensitivity analyses	NA
**Discussion**			
Key results	18	Summarise key results with reference to study objectives	6
Limitations	19	Discuss limitations of the study, taking into account sources of potential bias or imprecision. Discuss both direction and magnitude of any potential bias	8-9
Interpretation	20	Give a cautious overall interpretation of results considering objectives, limitations, multiplicity of analyses, results from similar studies, and other relevant evidence	7
Generalisability	21	Discuss the generalisability (external validity) of the study results	8-9
**Other information**			
Funding	22	Give the source of funding and the role of the funders for the present study and, if applicable, for the original study on which the present article is based	10

*Give information separately for exposed and unexposed groups.

Note: An Explanation and Elaboration article discusses each checklist item and gives methodological background and published examples of transparent reporting. The STROBE checklist is best used in conjunction with this article (freely available on the Web sites of PLoS Medicine at http://www.plosmedicine.org/, Annals of Internal Medicine at http://www.annals.org/, and Epidemiology at http://www.epidem.com/). Information on the STROBE Initiative is available at www.strobe-statement.org.

For peer review only

For peer review only - http://bmjopen.bmj.com/site/about/guidelines.xhtml
